# Synthesis of 2-Arylphenols via Formal Bismuth(V)-Mediated C–O Arylation of Guaiacols

**DOI:** 10.1021/acs.orglett.5c00593

**Published:** 2025-03-06

**Authors:** Natasha F. M. Ansarian, Liam T. Ball

**Affiliations:** School of Chemistry, University of Nottingham, Nottingham NG7 2RD, U.K.

## Abstract

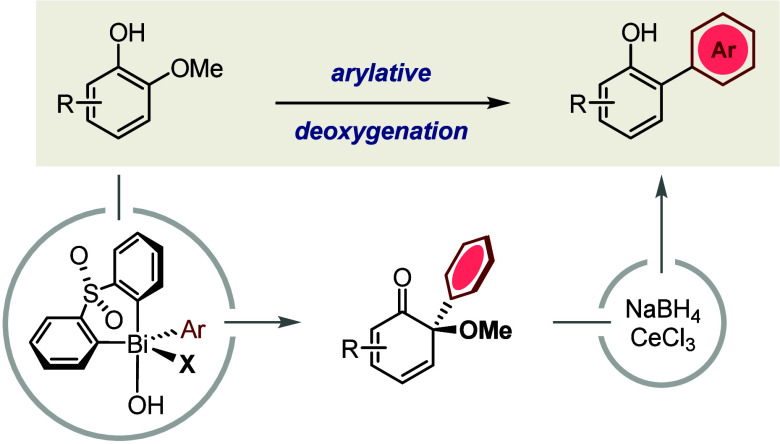

We report a new C–O arylation strategy for the synthesis of 2-arylphenols from bio-abundant guaiacols. Functionalization of the strong C–O bond is achieved through a sequence of arylative dearomatization, mediated selectively by an electrophilic bismuth(V) arylating agent, followed by 1,2-reduction and deoxygenative rearomatization. This methodology represents a rare example of complexity-generating deoxygenation applied to lignin-derived biorefinery feedstocks and provides independence from the regioselectivity rules established for the bismuth(V)-mediated arylation of simple phenols.

The guaiacol motif is a core unit of bio-abundant lignins^1^ and is present in a huge array of small-molecule natural products.^2−4^ Lignin derivatives are, however, often considered too highly oxygenated to be directly useful as green platform chemicals, and much effort has therefore been dedicated to their selective deoxygenation.^5−10^ In the context of fine chemical synthesis,^11^ deoxygenative functionalization of the guaiacol motif is achieved most easily for the hydroxyl moiety due to the propensity with which it can be preactivated toward oxidative addition (e.g., as a sulfonate ester).^12−14^ In contrast, metal-mediated methods for functionalization of the guaiacyl C_Ar_–OMe bond are rare; indeed, activation of any C_Ar_–OMe bond remains challenging.^15^ We anticipated that an alternative activation mode would provide a powerful complement to conventional metal-mediated hydrogenolysis methods, especially if it enabled selective C_Ar_–OMe activation in a way that added, rather than ablated, complexity.

As part of our ongoing program in Bi(V)-mediated electrophilic arylation,^16−21^ we recently reported on the arylative dearomatization of *o*-alkyl phenols (Scheme 1A).^22^ In that work, key arylating agent **3** was accessed from bench-stable bismacycle tosylate **1-OTs** by a modular sequence of B-to-Bi transmetalation and oxidation. We observed that the regioselectivity of subsequent phenol arylation was determined predominantly by the relative electronic properties of positions 2 and 6, rather than the steric profiles of their substituents.^22^ On this basis, we hypothesized that the reaction of guaiacols with Bi(V) reagents of type **3** would result in selective arylation at the more electron-rich position 2 (*ipso* to OMe), rather than at position 6 (*ipso* to H), and that this would provide a unique opportunity to activate the otherwise intransigent guaiacyl C_Ar_–OMe linkage (Scheme 1B).

**Scheme 1:**
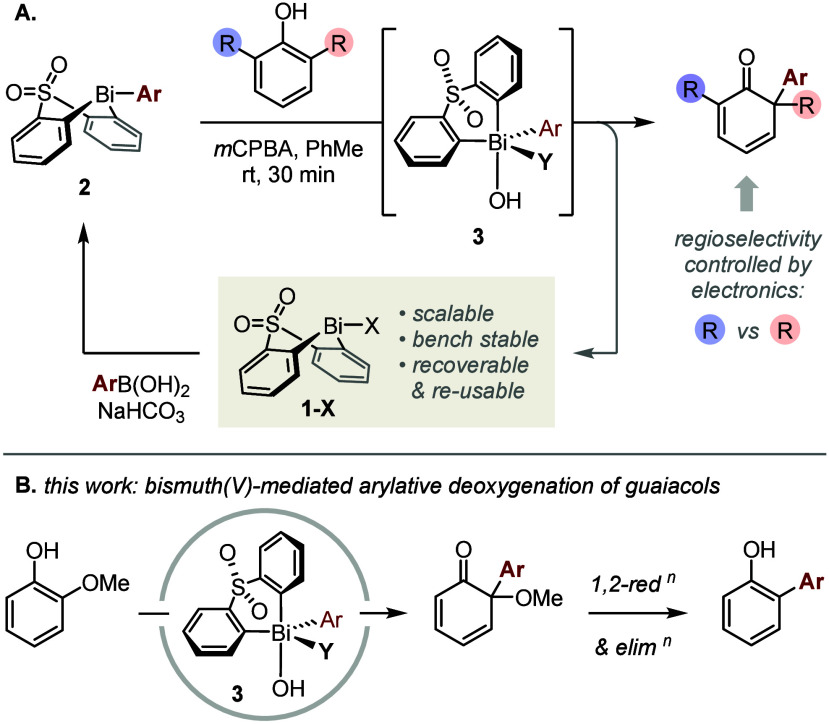
Bismuth-Mediated Dearomatization of Phenols

Furthermore, we anticipated that preferential arylation at a C–O bond rather than at a C–H bond would provide a level of substrate-independent regiocontrol that is not possible for the Bi(V)-mediated arylation of simple phenols. Specifically, where selection between the two C_*ortho*_–H positions of a phenol is determined by its C_*meta*_ substituents,^16^ discrimination between the inherently dissimilar C_*ortho*_–H and C_*ortho*_–OMe positions of a guaiacol would be dominated by the ether moiety and therefore would be largely independent of other substituents on the ring.

In this Letter, we show that the dealkoxylative arylation of guaiacols can be achieved through sequential Bi(V)-mediated electrophilic arylation and subsequent 1,2-reduction (Scheme 1B). This formal C_Ar_–OMe cross-coupling process is performed without exclusion of air or moisture and employs a bench-stable bismacycle that is accessible on scale^21^ and can be recovered for reuse. It provides a useful complement to the regioselectivity rules established previously for Bi(V)-mediated arylations of phenols and, ultimately, represents the first example of a guaiacol deoxygenation that generates complexity.

We initiated our studies of the arylative dearomatization of guaiacol **4** using preformed arylbismacycle **2a** (Table 1). Upon addition of *m*CPBA to guaiacol **4** and bismacycle **2a** in toluene, arylation is high yielding and fast (entry 1) and occurs with 5.9/1 regioselectivity in favor of position 2 (*ipso* to OMe). Monoaryl cyclohexadienone **5a** proved to be stable on silica gel for at least 1 h and, in the absence of light, as a room-temperature solution in cyclohexane (>90% remaining after 18 h, as determined by ^19^F NMR spectroscopic analysis vs an internal standard). However, this key intermediate decomposes to a complex mixture upon being heated to 80 °C (<15% remaining after 18 h) and is converted into the corresponding bicyclo[3.1.0]hexenone by irradiation with white light-emitting diodes for 1 h.^23,24^

**Table 1:**
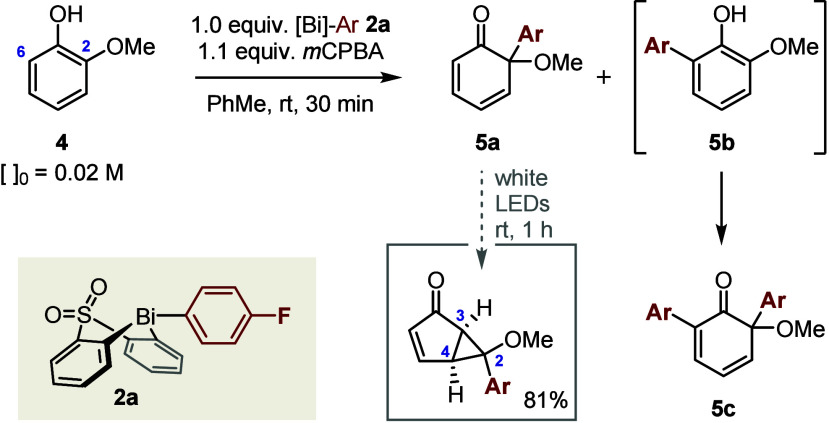
Optimization of the Arylative Dearomatization^a^

		yield (%)		
entry	variation from above	**5a**	**5c**	**5a**/**5c**
1	none	77	13	5.9
2	THF as the solvent	68	10	6.8
3	EtOAc as the solvent	63	10	6.3
4	PhCF_3_ as the solvent	59	10	5.9
5	DMF as the solvent	50	8	6.3
6	[**4**]_0_ = 0.04 M	68	10	6.8
7	[**4**]_0_ = 0.3 M	65	14	4.6
8	[**4**]_0_ = 0.4 M	50	8	6.3
9	1.5 equiv of **2a**, 1.65 equiv of *m*CPBA	75	10	7.5
10	1.5 equiv of **4**	75	10	7.5

a Yields determined by ^19^F NMR spectroscopy vs an internal standard (4,4′-bis(trifluoromethyl)biphenyl) and calculated relative to the guaiacol starting material.

Minor regioisomer **5b**, resulting from arylation at position 6 (*ipso* to H), is not observed but instead undergoes a second, rapid arylation at position 2 to give diaryl cyclohexadienone **5c**. The regioselectivity of the initial arylation is therefore represented by the ratio of monoaryl **5a** to diaryl **5c** and proved to be largely insensitive to the reaction solvent (Table 1, entries 2–5), concentration (entries 6–8), or reagent stoichiometry (entries 9 and 10).

We next investigated the 1,2-reduction/MeOH elimination that converts cyclohexadienone **5a** into 2-arylphenol **5** and completes the C_Ar_–OMe activation sequence (Table 2). To this end, passage of crude cyclohexadienone **5a** through basic alumina followed by Luche-type reduction^25^ afforded arylphenol **5** in excellent yield (entry 1). In contrast, omitting the CeCl_3_ additive gave only a complex mixture (entry 2), whereas using crude cyclohexadienone **5a** directly led to reduced, and highly variable, yields (entry 3). Additive studies (entry 4) revealed that this irreproducibility was ultimately caused by residual bismacycle *m*-chlorobenzoate (**1-OmCB**), the co-product of the arylation.

**Table 2:**
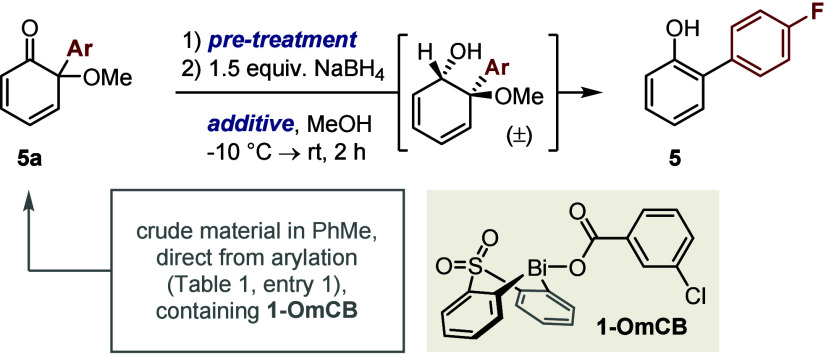
Optimization of 1,2-Reduction/Elimination^a^

entry	pretreatment	additive(s) (equiv)	yield of **5** (%)
1	basic alumina	CeCl_3_·H_2_O (1.5)	>99
2	basic alumina	none	<5
3	none	CeCl_3_·H_2_O (1.5)	20–50
4	basic alumina	CeCl_3_·H_2_O (1.5), **1-OmCB**(1.0)	<5

a Yields determined by ^19^F NMR spectroscopy vs an internal standard (4,4′-bis(trifluoromethyl)biphenyl) and calculated relative to the guaiacol starting material. The relative stereochemistry of the dienol intermediate is assigned by analogy to isolated compound **12e** (Scheme 2A). “Basic alumina” indicates that the starting material was passed through a pad of basic alumina, eluting with EtOAc, before use.

The combined, optimized procedure for the arylative dealkoxylation of guaiacols is shown in Scheme 2A. The initial arylation was compatible with both electron-rich (**6** and **7** (Scheme 2B)) and electron-poor (**5** and **8**–**12**) boronic acids, providing rapid access to a diverse array of cyclohexadienones in good yields. The 1-naphthyl moiety was installed in modest yield (**13**), but as observed in our previous work,^22^*ortho*-substituted aryl moieties were not tolerated, presumably due to developing steric strain at the incipient quaternary α-center. Attempts to extend the methodology to heterocyclic boronic acids were hindered by the instability of the corresponding cyclohexadienones to basic alumina (**14**) or their propensity toward Diels–Alder dimerization (**15**; 40% dimer formed by the end of the arylation step).

**Scheme 2:**
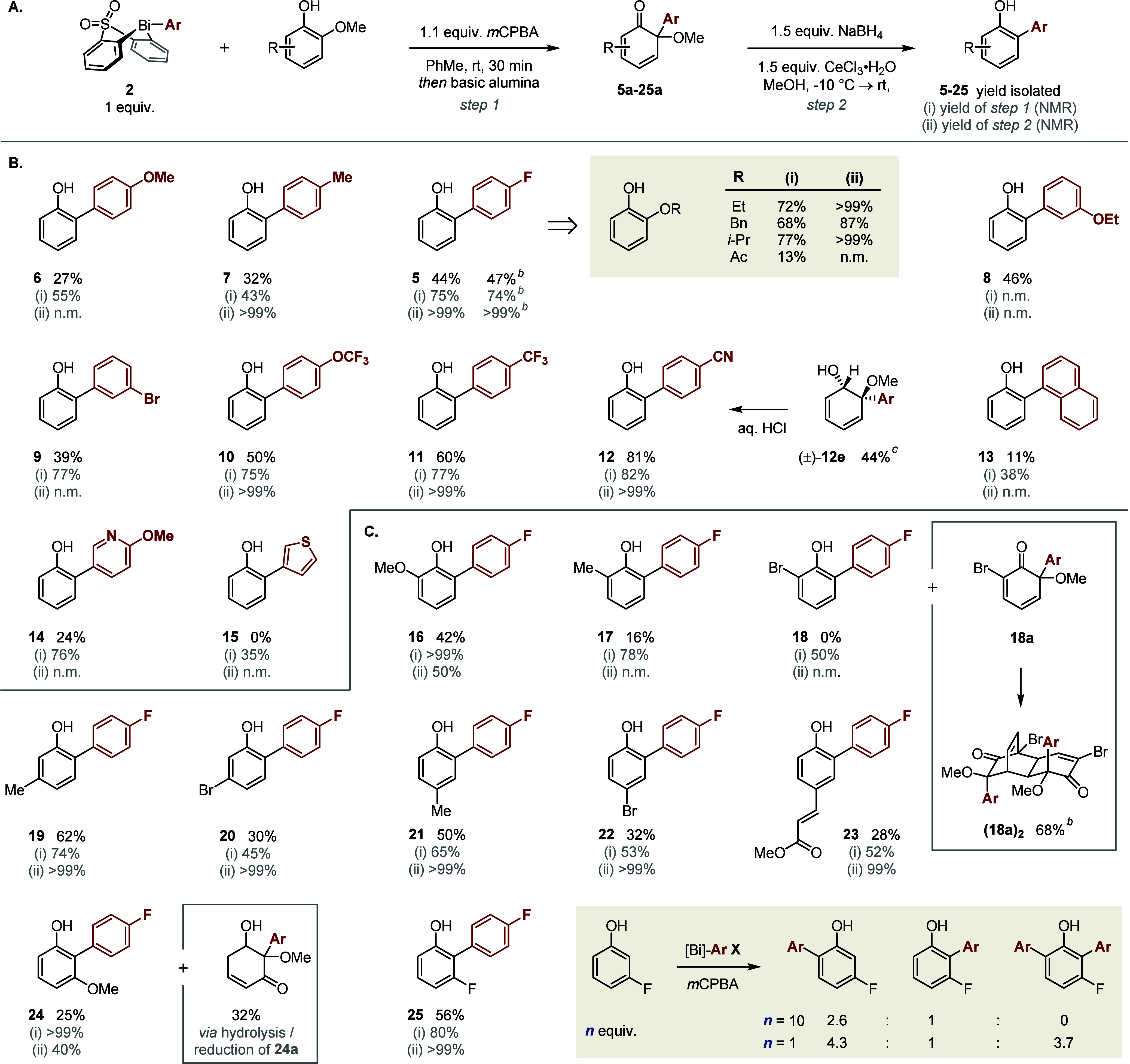
Scope of the Bismuth(V)-Mediated Arylative Dealkoxylation of Guaiacols Yields of arylphenols refer to material isolated following purification and are calculated across both steps relative to the guaiacol starting material. Yields for step 1 are determined by NMR spectroscopic analysis vs an internal standard and refer to the yield of cyclohexadienone intermediate calculated relative to the guaiacol starting material. Yields for step 2 are determined by NMR spectroscopic analysis vs an internal standard and refer to the yield of the arylphenol product calculated relative to the cyclohexadienone intermediate. Reaction performed using 1.0 mmol of guaiacol **4**. Yield determined by NMR spectroscopic analysis. n.m. = not measured; the yield could not be measured reliably due to insufficient resolution of NMR signals or the instability of the intermediate cyclohexadienone.

Notably, 2,4-cyclohexadienones of type **5a**–**25a** have found widespread application in synthesis^26^ but are typically prepared via oxidative alkoxylation of an *ortho*-substituted phenol.^27−29^ By achieving dearomatization through installation of an aryl moiety, rather than an alkoxy moiety, our strategy provides a much more modular disconnection, and one that has previously been demonstrated in only a single example (48% yield, using a stoichiometric aryllead(IV) reagent).^30^

The subsequent Luche reduction/elimination sequence proved to be particularly robust, ultimately enabling the net conversion of biorefinery feedstock **4** into a diverse range of value-added 2-arylphenols bearing synthetically useful functional groups (e.g., **9** and **12**). Rearomatization typically occurred under the reduction conditions, such that, for the majority of substrates, the corresponding cyclohexadienol intermediate could not be observed. However, the elimination of MeOH from dienols bearing very electron deficient aryl moieties (**11** and **12**) proved to be slow and required addition of a Brønsted acid. This relative stability allowed for characterization of cyano-substituted dienol **12e** as a single diastereoisomer, arising from the expected^31^ addition of hydride *syn* to the methoxy substituent. The observed effects of electronics and added acid on elimination rate, as well as stereochemical considerations,^32^ suggest that the rearomatization process occurs via an E1-type mechanism.

Alkoxy substituents other than methoxy were very well tolerated in both steps (Scheme 2B, inset), hinting at the broader compatibility of our deoxygenative arylation with more complex lignin derivatives. As anticipated, 2-acetoxy phenol reacted to give the corresponding 2,6-diaryl cyclohexadienone as the major product (62%, as determined by ^19^F NMR spectroscopic analysis), presumably due to the net electron-withdrawing character of the acyloxy substituent^33^ that favors initial arylation at position 6.

Extension of our methodology to substituted guaiacols (Scheme 2C) was more nuanced, primarily due to the variable stability of the intermediate cyclohexadienones. For example, while syringol (6-methoxyguaiacol) reacted cleanly in both steps to give arylphenol **16**, arylation of 6-methyl or 6-bromo guaiacols was followed by rapid Diels–Alder dimerization that precluded the synthesis of **17** or **18** in useful yields. The propensity of 6-methyl dienones toward dimerization has been noted previously,^34^ whereas the observed reactivity of the 6-bromo dienone is consistent with the Danishefsky–Houk halide effect.^35−38^

In contrast, substitution at positions 5 and 4 was generally well tolerated (**19**–**23**). This suggests a greater stability of the intermediate dieonones, as has been reported for analogous masked *o*-benzoquinones.^39^

Our arylation/deoxygenation sequence proved to be compatible with guaiacols bearing both electron-donating and electron-withdrawing substituents at position 3 (**24** and **25**). The ability to access 2,3-disubstituted phenols in this way provides a useful complement to other, better established phenol arylation strategies. For example, the synthesis of 2-halo-3-substituted phenols, required as precursors in cross-coupling approaches, is generally not possible via direct electrophilic halogenation^40,41^ and typically requires multistep sequences based on directed *ortho*-metalation.^42^ Similarly, application of our previously reported method for Bi(V)-mediated C–H arylation to 3-methoxyphenol gives the 6-arylated product in >20/1 selectivity.^16^ In contrast, and as demonstrated in this work, using the guaiacyl methoxy substituent as a traceless activator reverses the regioselectivity of arylation and allows access to a substitution pattern that otherwise cannot be accessed by using organobismuth chemistry. This complementarity is perhaps even more stark when comparing the arylation of 3-fluoroguaiacol and 3-fluorophenol. The latter arylates primarily at position 6 (2.6/1)^16^ and must be used in significant excess to outcompete yield-attenuating diarylation (Scheme 2C, inset). In contrast, the method reported here not only gives preferential arylation at position 2, leading to the conventionally “disfavored” regioisomer, but also prevents overarylation: arylation *ipso* to the alkoxy substituent prevents further reaction, a benefit that is general to all guaiacol substrates.

Finally, the whole process, from the initial B-to-Bi transmetalation to the final elimination of MeOH, can be performed as a single telescoped operation without the need for full chromatographic purification of the reaction intermediates (Scheme 3). Importantly, bismacycle *m*-chlorobenzoate **1-OmCB** can be recovered in excellent yield following precipitation and filtration; this material can be converted back to the corresponding tosylate or bromide or indeed can be reused directly in transmetalation without further purification.^22^ It is thus possible not only to access the bismacycle conveniently on scale^21^ but also, in this way, to minimize its losses in the arylation process and ultimately improve atom economy.

**Scheme 3:**
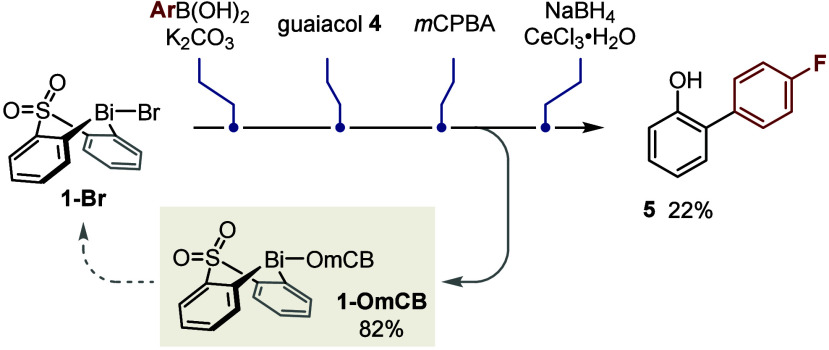
Telescoped Arylation and Bismacycle Recovery Yields refer to isolated material.

In conclusion, we have developed the first guaiacol C–O arylation to employ the C_Ar_–OMe ether linkage as a formal coupling partner. Our methodology relies on the selectivity of Bi(V)-mediated electrophilic arylation for more substituted position 2 of the guaiacol rather than unsubstituted position 6. This arylative dearomatization provides highly modular access to 2,4-cyclohexadienones that have previously been prepared by oxygenation and that, as we show, can serve as activated intermediates en route to 2-arylphenols. The deoxygenation is completed by 1,2-reduction and elimination, with the latter being posited to occur through an E1-type mechanism. Despite the highly variable stability of substituted 2,4-cyclohexadienones, this strategy allows for the overall deoxygenative upgrading of the guaiacol motif, which is common to biomass-derived feedstocks and valuable natural products alike. The formal C–O arylation process has two additional benefits: (1) dearomatization prevents further arylation, thereby preventing over-arylation and allowing the use of more economic stoichiometries of reagents, and (2) it overcomes the innate regioselectivity expected for a given phenolic core and therefore can be used to access 2-arylphenols that are otherwise not accessible using methods based on electrophilic substitution, such as Bi(V)-mediated C–H arylation or halogenation/cross-coupling.

## Data Availability

The data underlying this study are available in the published article and its Supporting Information.

## Supporting Information Available

Experimental procedures, spectra, and optimization data (PDF)
