# Supplementary material for: Synthesis of 2-Arylphenols via Formal Bismuth(V)-Mediated C–O Arylation of Guaiacols
Source: Org Lett. 2025 Mar 6;27(11):2769–74. doi: 10.1021/acs.orglett.5c00593 (PMC11934140; doi:10.1021/acs.orglett.5c00593)
Supplement: Supplementary file 1 — ol5c00593_si_001.pdf [file ol5c00593_si_001.pdf]

SUPPORTING INFORMATION

Synthesis of 2-Arylphenols *via* Formal Bismuth(V)-Mediated C-O Arylation of Guaiacols

Natasha F. M. Ansarian and Liam T. Ball*

*Email: liam.ball@nottingham.ac.uk

orcid: N.F.M.A., 0009-0003-2862-2637; L.T.B., 0000-0003-3849-9006.

School of Chemistry, University of Nottingham, Nottingham NG7 2RD, U.K.

Table of Contents

1. General Experimental Information 3

2. Additional Optimisation Experiments 6

2.1 Optimisation of Arylative Dearomatisation 6

a. Effect of Concentration 6

b. Effect of Solvent 7

c. Effect of Reagent Stoichiometry 8

d. Identification of Photodecomposition Product 9

2.2 Optimisation of Luche Reduction / Aromatisation 10

3. Synthesis and Characterization of Organobismuth Compounds 12

3.1 Synthesis of Bismacycle Precursors 12

4. Synthesis and Characterisation of Guaiacol Substrates 16

5. Synthesis and Characterisation of *ortho*-Arylated Phenols 19

5.1 General Procedure for Dealkoxylative Arylation (GP-3) 19

5.2 Characterisation Data for *ortho*-Aryl Phenols 20

5.3 Procedure for Dealkoxylative Arylation on a 1.0 mmol Scale 48

6. Telescoped Transmetallation, Arylation and Bismacycle Recovery 49

7. Synthesis and Characterisation of Authentic Compounds 51

8. NMR Spectra 52

9. References 124

1. General Experimental Information

Procedures employing oxygen- and/or moisture-sensitive materials were performed with anhydrous solvents using standard inert-atmosphere techniques (atmosphere of anhydrous dinitrogen). Analytical thin-layer chromatography was performed on precoated aluminium-backed plates (Silica Gel 60 F254; Merck), and visualized using a combination of UV light (254 nm) and acidic ethanolic vanillin, aqueous basic potassium permanganate, methanolic iron chloride or iodine stains. Manual flash column chromatography was performed using Scharlab 60 silica gel (35-70 mesh); automated flash column chromatography was performed on disposable columns pre-packed with 50 µm spherical silica gel using a Büchi C-850 or C-815 equipped with a UV-vis DAD (200-800 nm) and an ELSD.

NMR spectra were recorded at 25 °C on Bruker Avance 500 or 400 spectrometers (1H, 500 / 400 MHz; $^{13}C\{^{1}H\}$, 125 / 100 MHz; ^{19}F NMR, 471 / 376 MHz). Chemical shifts are reported in ppm; coupling constants, *J*, are reported in Hz and are uncorrected for digitization. The following abbreviations (and their combinations) are used to label the multiplicities: s (singlet), d (doublet), t (triplet), q (quartet), quint (quintet), sept (septet), m (multiplet) and br (broad), and app (apparent). Structural assignments were made with the assistance of COSY, HSQC, HMBC and NOESY.

^{1}H and $^{13}C\{^{1}H\}$ chemical shifts are reported relative to tetramethylsilane, and are referenced to the appropriate residual solvent peaks:

- $CDCl_3$: δ_H = 7.26 ppm, δ_C = 77.16 ppm
- DMSO-d_6: δ_H = 2.50 ppm, δ_C = 39.52 ppm

^{19}F chemical shifts are reported relative to $CFCl_3$.

Infrared spectra of neat compounds were recorded over the range 4000-600 cm^{-1} using either a PerkinElmerSpectrum 1000 Series FTIR spectrometer with an ATR diamond

cell, or a Bruker Alpha FTIR spectrometer fitted with a Bruker Platinum ATR QuicksnapTM diamond cell. Melting points were measured using Stuart SMP10 or Gallenkamp melting point apparatus in open capillaries. High resolution electrospray ionization mass spectra (HRMS) were recorded using a Bruker ESI-TOF MicroTOF II spectrometer.

Reagent grade solvents (Fisher Technical) were employed. CH_2Cl_2 was dried over activated 3Å molecular sieves. THF was dried using an Inert PureSolv Grubbs-type system (activated alumina columns, argon atmosphere). All reagents were obtained from commercial sources and, with the following exceptions, were used as received:

- $BiBr_3$ (99.9%; Acros) was dried by stirring under vacuum (*ca* 10^{-2} mbar) at 40 °C for 1 h before use.
- *n*-Butyllithium (*ca* 2.5 M in hexanes; Sigma-Aldrich) was titrated against *N*-benzyl benzamide before use.
- *m*CPBA (*ca* 70%; Sigma-Aldrich) was titrated against triphenylphosphine before use.
- Bismacycle bromide **1-Br** was prepared according to literature procedure.[1]
- Aryl bismacycles were prepared according to the following literature procedures:

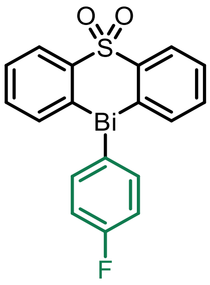

Reference [2]

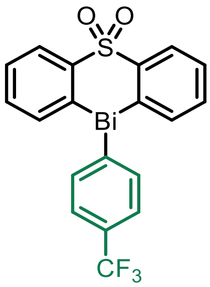

Reference [2]

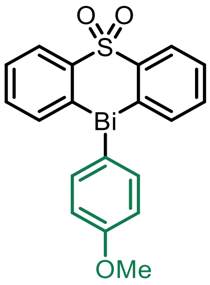

Reference [2]

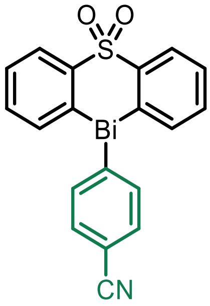

Reference [2]

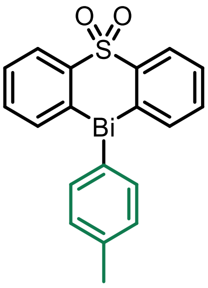

Reference [2]

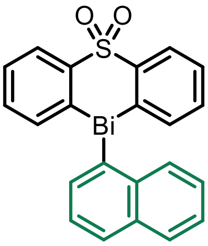

Reference [2]

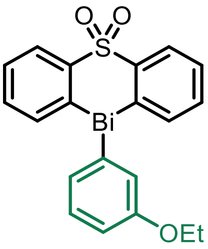

Reference [1]

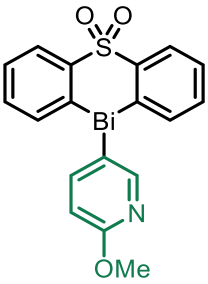

Reference [1]

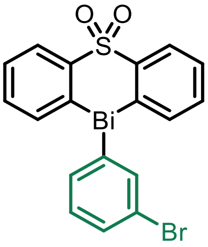

Reference [1]

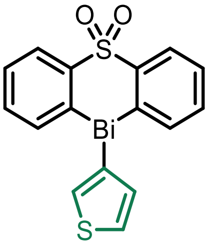

Reference [1]

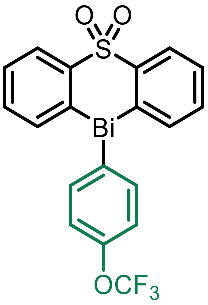

Reference [1]

2. Additional Optimisation Experiments

2.1 Optimisation of Arylative Dearomatisation

a. Effect of Concentration

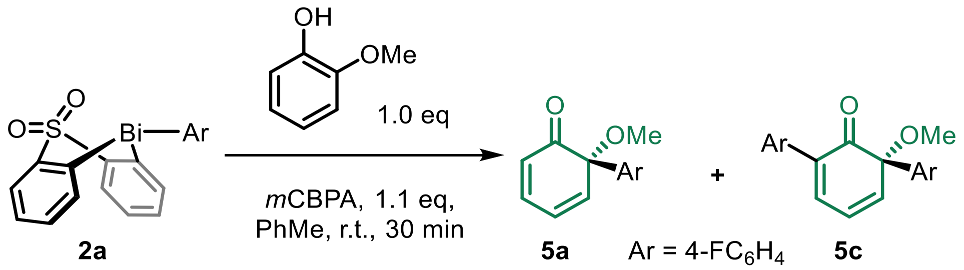

entry	$[2a]_0$ /M	% 5a	% 5c	5a : 5c
1	0.02	77	13	5.9
2	0.04	68	10	6.8
3	0.3	65	14	4.6
4	0.4	50	8	6.3

Table S1: Effect of varying concentration of arylative dearomatisation. Yields determined by ^{19}F NMR spectroscopy vs internal standard (4,4'-bis(trifluoromethyl)biphenyl).

Aryl bismacycle **2a** (104 mg, 0.2 mmol) and guaiacol (24.8 mg, 0.2 mmol) were suspended in PhMe in the presence of internal standard (4,4'-bis(trifluoromethyl)-1,1'-biphenyl; 5.0 mg, 0.02 mmol) at rt. *m*CPBA (44 mg, 0.22 mmol, 85% purity) was added in 10 equal portions at 30 second intervals while stirring, and the reaction mixture was left for 30 min. The resulting orange solution was quenched with sat. aq. $Na_2S_2O_5$ (0.01 mL), left to stir for 5 min and concentrated *in vacuo*. The residue was dissolved in $CDCl_3$ and analysed by ^{1}H NMR spectroscopy and quantitative ^{19}F NMR spectroscopy.

b. Effect of Solvent

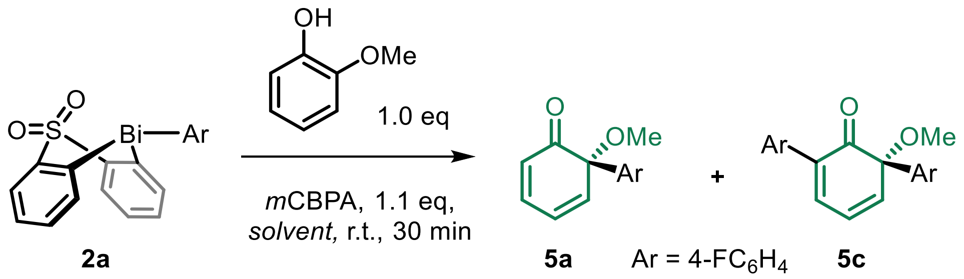

entry	solvent	% 5a	% 5c	5a : 5c
1	PhMe	77	13	5.9
2	THF	68	10	6.8
3	EtOAc	63	10	6.3
4	$PhCF_3$	59	10	5.9
5	DMF	50	8	6.3

Table S2: Effect of varying solvent on the arylative dearomatisation. Yields determined by ^{19}F NMR spectroscopy vs internal standard (4,4'-bis(trifluoromethyl)biphenyl).

Aryl bismacycle **2a** (51 mg, 0.1 mmol) and guaiacol (12.8 mg, 0.1 mmol) were suspended in the solvent stated for each entry in the presence of internal standard (4,4'-bis(trifluoromethyl)-1,1'-biphenyl; 5.0 mg, 0.02 mmol) at rt. *m*CPBA (27 mg, 0.11 mmol, 70% purity) was added in 10 equal portions at 30 second intervals while stirring, and the reaction mixture was left for 30 min. The resulting orange solution was quenched with sat. aq. $Na_2S_2O_5$ (0.01 mL), left to stir for 5 min and concentrated *in vacuo*. The residue was dissolved in $CDCl_3$ and analysed by ^1H NMR spectroscopy and quantitative ^{19}F NMR spectroscopy.

c. Effect of Reagent Stoichiometry

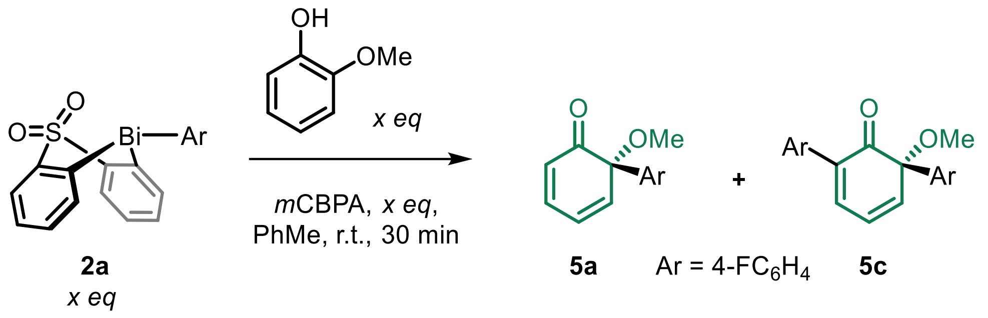

entry	equiv. 2a	equiv. guaiacol	equiv. *m*CPBA	% 5a	% 5c	5a : 5c-
1	1	1	1.1	77	13	5.9
2	1.5	1	1.65	75	10	7.5
3	1	1.5	1.1	75	10	7.5

Table S3: Effect of varying equivalents of cpd-x and cpd-x on the arylative dearomatisation. Yields determined by ^{19}F NMR spectroscopy vs internal standard (4,4'-bis(trifluoromethyl)biphenyl).

Aryl bismacycle **2a** and guaiacol substrate (limiting reagent = 0.1 mmol) were suspended in PhMe (5 mL) in the presence of internal standard (4,4'-bis(trifluoromethyl)-1,1'-biphenyl; 5.0 mg, 0.02 mmol) at rt. *m*CPBA was added in 10 equal portions at 30 second intervals while stirring, and the reaction mixture was left for 30 min. The resulting orange solution was quenched with sat. aq. $Na_2S_2O_5$ (0.01 mL), left to stir for 5 min and concentrated *in vacuo*. The residue was dissolved in $CDCl_3$ and analysed by ^1H NMR spectroscopy and quantitative ^{19}F NMR spectroscopy.

d. Identification of Photodecomposition Product

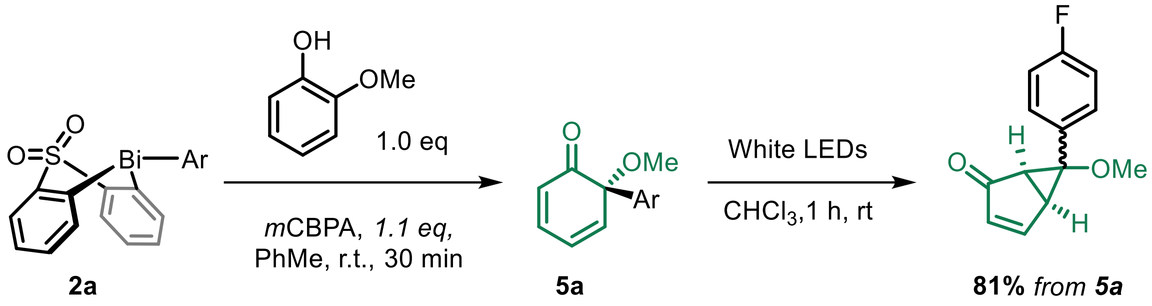

*Figure S1: Photodecomposition of **5a**. Yield determined by ^{19}F NMR spectroscopy vs internal standard (4,4'-bis(trifluoromethyl)biphenyl).*

Aryl bismacycle **2a** (364 mg, 0.7 mmol) and guaiacol (86.8 mg, 0.7 mmol) were suspended in PhMe in the presence of internal standard (4,4'-bis(trifluoromethyl)-1,1'-biphenyl; 0.02 mmol) at rt. *m*CPBA (192 mg, 0.77 mmol, 70% purity) was added in 10 equal portions at 30 second intervals while stirring, and the reaction mixture was left for 30 min. The resulting orange solution was quenched with sat. aq. $Na_2S_2O_5$ (0.01 mL), left to stir for 5 min, then passed through a plug of basic alumina (*ca* 21 mL). The intermediate cyclohexadienone was eluted with EtOAc (70 mL) and concentrated *in vacuo* to afford cyclohexadienone **5a**. The residue was dissolved in $CHCl_3$ (*ca* 5 mL) and irradiated with white LEDs for 1 h at rt under an aerobic atmosphere. The reaction mixture was then concentrated *in vacuo* and analysed by ^{1}H NMR spectroscopy and quantitative ^{19}F NMR spectroscopy (81% yield of a single diastereoisomer, determined by NMR spectroscopy). The bicyclo[3.1.0]hexenone structure was confirmed by 2D NMR spectroscopy; the relative stereochemistry could not be determined unambiguously.

^{1}H NMR (500 MHz, C_6D_6): δ_H 7.04 (2H, dd, J = 8.6, 5.4 Hz), 6.64 (2H, app. t, J = 8.6 Hz), 6.40 (1H, ddd, J = 5.6, 2.8, 0.9 Hz), 5.31 (1H, dd, J = 5.6, 1.0 Hz), 2.69 (3H, s), 2.58 (1H, dd, J = 5.4, 2.7 Hz), 2.42 (1H, app. dt, J = 5.4, 1.0 Hz).

^{13}C{^{1}H} NMR (126 MHz, C_6D_6) δ_C 201.7, 163.1 (d, J = 247.6 Hz), 156.7, 133.7 (2 × C, determined by HSQC, including: d, J = 8.9 Hz), 127.7 (quaternary carbon obscured by C_6D_6 peak, determined by HMBC), 115.3 (d, J = 21.7 Hz), 86.9, 54.3, 36.6, 35.9.

^{19}F NMR (377 MHz, $CDCl_3$) δ_F -113.5 (tt, J = 8.6, 5.4 Hz).

HRMS (ESI$^+$): m/z calcd. for $C_{13}H_{11}FO_2$ [M+H]$^+$: 219.0821. Found: 219.0815.

2.2 Optimisation of Luche Reduction / Aromatisation

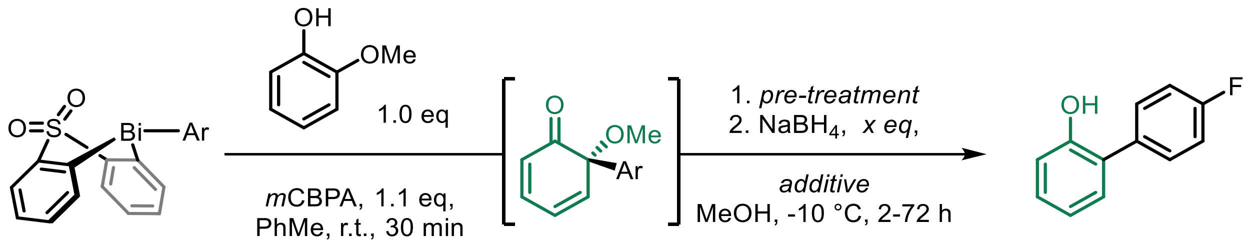

entry	pre-treatment	additive (equiv.)	$NaBH_4$ (equiv.)	% 5
1	solvent swap	$CeCl_3$•H_2O (1.0)	1.0	20-50
2	basic alumina, then solvent swap	None	1.0	<5
3	basic alumina, then solvent swap	$CeCl_3$•H_2O (1.5)	1.5	>99
4	basic alumina, then solvent swap	$CeCl_3$•H_2O (1.5)<br>**1-OmCB** (1.0)	1.5	<5

Supplementary Table 4: Variation from standard Luche reduction- rearomatisation conditions. Yields determined by ^{19}F NMR spectroscopy vs internal standard (4,4'-bis(trifluoromethyl)biphenyl).

Entry 1: Aryl bismacycle **2a** (103 mg, 0.2 mmol) and guaiacol (24.8 mg, 0.2 mmol) were suspended in PhMe (2 mL) in the presence of internal standard (4,4'-bis(trifluoromethyl)-1,1'-biphenyl; 0.117 mmol) at rt. *m*CPBA (54 mg, 0.22 mmol, 69% purity) was added in 10 equal portions at 30 second intervals while stirring, and the reaction mixture was left for 30 min. The resulting orange solution was quenched with sat. aq. $Na_2S_2O_5$ (0.01 mL), then concentrated into a microwave vial containing cerium chloride hydrate (75.5 mg, 0.2 mmol). This was diluted with MeOH (1.0 mL) and cooled to -10 °C, after which time sodium borohydride (7.56 mg, 0.2 mmol) was added in 10 equal portions at 40 second intervals while stirring. The reaction was left to warm to rt over 2 h and quenched with aq. HCl (1 M, 1.0 mL), left to stir for 5 min, then concentrated *in vacuo*. The residue was dissolved in $CDCl_3$ and analysed by ^1H NMR spectroscopy and quantitative ^{19}F NMR spectroscopy.

Entry 2: In an amberised RBF, aryl bismacycle **2a** (130 mg, 0.25 mmol) and guaiacol (30 mg, 0.25 mmol) were suspended in PhMe (13 mL) in the presence of internal standard (4,4'-bis(trifluoromethyl)-1,1'-biphenyl; 5.0 mg 0.02 mmol) at rt. *m*CPBA (69 mg, 0.275 mmol, 69%

purity) was added in 10 equal portions at 30 second intervals while stirring, and the reaction mixture was left for 30 min. The resulting orange solution was quenched with sat. aq. $Na_2S_2O_5$ (0.01 mL), then concentrated *in vacuo.* This was diluted with MeOH (2.5 mL) and cooled to -10 °C, after which time sodium borohydride (14.2 mg, 0.375 mmol) was added in 10 equal portions at 40 second intervals while stirring. The reaction was left to warm to rt over 2 h and quenched with aq. HCl (4 M, 1.0 mL), left to stir for 5 min, then concentrated *in vacuo.* The residue was dissolved in $CDCl_3$ and analysed by ^{1}H NMR spectroscopy and quantitative ^{19}F NMR spectroscopy.

<u>Entry 3:</u> *Performed according to GP-3, with yield determined by ^{1}H NMR spectroscopy and quantitative ^{19}F NMR spectroscopy.*

<u>Entry 4:</u> In an amberised RBF, aryl bismacycle **2a** (104 mg, 0.2 mmol) and guaiacol (25 mg, 0.2 mmol) were suspended in PhMe (10 mL) in the presence of internal standard (4,4'-bis(trifluoromethyl)-1,1'-biphenyl; 5.0 mg, 0.02 mmol) at rt. *m*CPBA (54 mg, 0.22 mmol, 69% purity) was added in 10 equal portions at 30 second intervals while stirring, and the reaction mixture was left for 30 min. The resulting orange solution was quenched with sat. aq. $Na_2S_2O_5$ (0.01 mL), left to stir for 5 min, then passed through a plug of basic alumina (*ca* 6 mL). The intermediate cyclohexadienone was eluted with EtOAc (10 mL) and concentrated into a flask containing cerium chloride hydrate (111 mg, 0.3 mmol) and **1-OmCB** (116 mg, 0.2 mmol). This was diluted with MeOH (2 mL) and cooled to -10 °C, after which time sodium borohydride (11.34 mg, 0.3 mmol) was added in 10 equal portions at 40 second intervals while stirring. The reaction was left to warm to rt over 2 h. The reaction mixture was then quenched with aq. HCl (4 M, 1 mL), left to stir for 5 min, then concentrated *in vacuo.* The residue was dissolved in $CDCl_3$ and analysed by ^{1}H NMR spectroscopy and quantitative ^{19}F NMR spectroscopy.

3. Synthesis and Characterization of Organobismuth Compounds

3.1 Synthesis of Bismacycle Precursors

Tri(4-fluorophenyl)bismuth

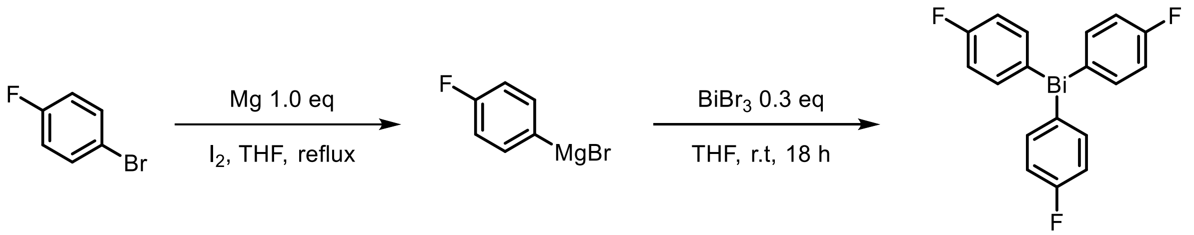

A flame dried flask was evacuated and back-filled three times with anhydrous dinitrogen before magnesium turnings (8.05 g, 330 mmol) and 2-3 crystals of iodine was added under a flow of nitrogen. Anhydrous THF (150 mL) was added *via* cannula on stirring and the suspension was cooled to 0 °C, before the dropwise addition of 4-bromofluorobenzene (36.3 mL, 330 mmol) *via* syringe over 20 min to generate the Grignard reagent. The reaction mixture was heated at reflux (aluminium heating block) for 1 h.

A second flame dried flask containing bismuth tribromide (44.8 g, 100 mmol) was evacuated and back-filled three times with anhydrous dinitrogen and dissolved in a minimum volume of anhydrous THF. The solution of Grignard reagent was added dropwise *via* cannula and the reaction mixture was left to stir for 18 h at rt. The reaction was quenched with methanol (30 mL), filtered through a pad of silica gel (5 mL) and eluted with Et_2O (2 × 50 mL). The resultant solution was stirred with water (50 mL) for 5 min and the aqueous phase was separated and extracted with Et_2O (3 × 10 mL). The collected organic portions were dried over $MgSO_4$, filtered through a pad of silica gel (5 mL) once more, eluted with Et_2O (2 × 50 mL) and the solvents removed in *vacuo* to obtain the crude triaryl bismuth. The solids were recrystallised from the minimum volume of hot ethanol to afford the title compound as an off white powder (29.4 g, 59.5 mmol, 60%). Characterisation data were consistent with literature values: ^{1}H, $^{13}C\{^{1}H\}$, ^{19}F NMR and MP.[3]

^{1}H NMR (400 MHz, $CDCl_3$): δ_H 7.64 (6H, dd, *J* = 8.5, 6.2 Hz), 7.08 (6H, app. t, *J* = 9.4).

^{13}C{^{1}H} NMR (101 MHz, $CDCl_3$): δ_C 163.0 (d, *J* = 247.3), 149.7, 139.3 (d, *J* = 7.0), 118.1 (d, *J* = 19.8).

^{19}F NMR (376 MHz, $CDCl_3$): δ_F -112.8 (tt, *J* = 9.4, 6.0 Hz).

IR (ATR-IR, neat) υ_{max} /cm^{-1}: 2980, 1895, 1710, 1638, 1572, 1482, 1442, 1406, 1382, 1310, 1268, 1209, 1156, 1084, 1043, 1013, 981.

HRMS (ESI$^-$): *m/z* calcd. for $C_{12}H_8BiF_2$ [M-Ar^F]$^-$: 399.0403. Found: 399.0403.

MP: 95-96 °C.

5,5-Dioxido-10H-dibenzo[b,e][1,4]thiabismin-10-yl 4-methylbenzenesulfonate (1-OTs)

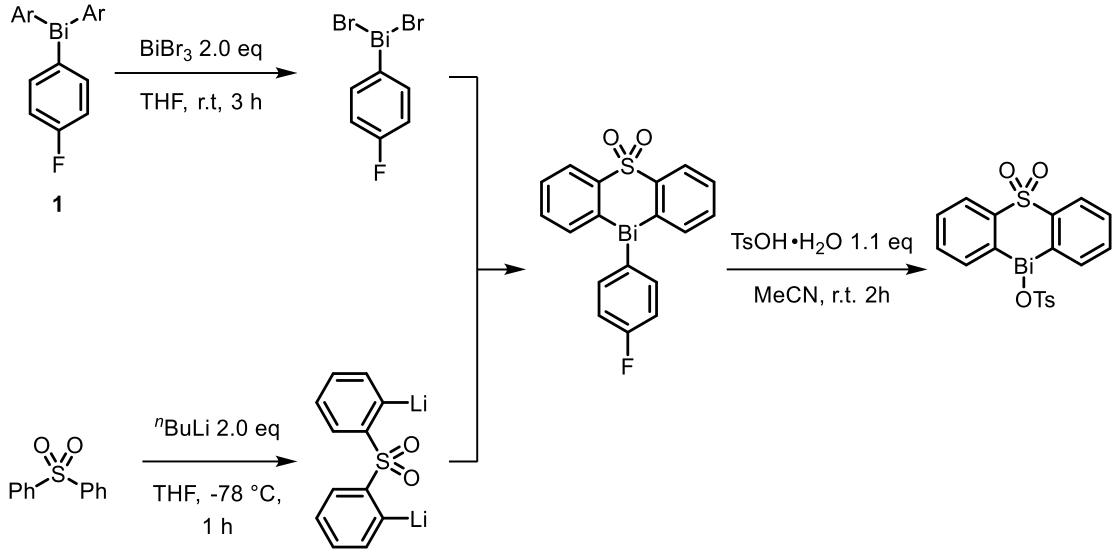

A flame dried flask containing diphenylsulfone (6.55 g, 30.0 mmol) was evacuated and back-filled three times with anhydrous dinitrogen before anhydrous THF was added (60 mL). The solution was cooled to -78 °C before *n*BuLi (2.5 M in hexanes; 24 mL, 60.0 mmol) was added dropwise *via* syringe over 20 min and left to stir for 1 h at -78 °C, forming a cream suspension.

A flame dried flask containing tri(4-fluorophenyl)bismuthane (4.94 g, 10.0 mmol) and $BiBr_3$ (9.02 g, 20.0 mmol) was evacuated and backfilled three times with anhydrous dinitrogen before anhydrous THF (60 mL) was added at -78 °C. The resulting yellow suspension was stirred for 3 h at rt. This was then added dropwise into the previously prepared suspension of dilithiodiphenylsulfone *via* cannula, and the resulting orange suspension was left to stir at rt over 18 h.

MeOH (30 mL) was added to the suspension and stirred for 5 min before it was concentrated *in vacuo*. The remaining solids were dissolved in DCM (100 mL) and filtered through a pad of silica gel (5 mL) and eluted with DCM (2 × 100 mL) to afford a yellow solution which was then vigorously stirred with aqueous saturated $NaHCO_3$ (100 mL) for 30 min. The aqueous portions were separated and extracted with DCM (3 × 20 mL), and the combined organic portions were dried over $MgSO_4$, filtered through a pad of silica gel (5 mL) once again, eluted with DCM (2 × 100 mL) then concentrated *in vacuo* to afford the intermediate aryl bismacycle **2a** as an off-white foam.

The crude aryl bismacycle **2a** was dissolved in MeCN (60 mL) at rt., *p*-toluenesulfonic acid monohydrate (6.27 g, 33.0 mmol) was added and left to stir. After 2 h, the white suspension was

filtered under reduced pressure through glass fibre filter paper and the resulting solid was washed with cold MeCN (3 × 20 mL), then Et_2O (20 mL) to afford **1-OTs** as a colourless solid (11.4 g, 21.9 mmol, 73% over 2 steps from tri(4-fluorophenyl)bismuth. Characterisation data were consistent with literature values: ^{1}H, and MP. [2]

^{1}H NMR (400 MHz, $CDCl_3$): δ_H 8.93 (2H, dd, *J* = 7.4, 0.6 Hz), 8.40 (2H, dd, *J* = 7.7, 0.7 Hz), 7.87-7.77 (4H, m; including app. td at 7.79, *J* = 7.5, 1.3 Hz) 7.50 (2H, app. td, *J* = 7.6, 1.1), 7.29 (2H, *J* = 8.2), 2.42 (3H, s).

^{1}H NMR (400 MHz, DMSO-*d*$_6$): δ_H 8.82 (2H, dd, *J* = 7.3, 1.2 Hz), 8.46 (2H, dd, *J* = 7.7, 1.2 Hz), 7.88 (2H, app. td, *J* = 7.4, 1.2 Hz), 7.55 (2H, app. td, *J* = 7.7, 1.2 Hz), 7.48 (2H, d, *J* = 8.1 Hz), 7.11 (2H, d, *J* = 7.9), 2.29 (3H, s)

^{13}C{^{1}H} NMR (101 MHz, DMSO-*d*$_6$): δ_C 201.7, 145.8, 141.9, 137.6, 136.2, 135.3, 128.8, 128.0, 127.2, 125.5, 20.8.

IR (ATR-IR, neat) υ_{max}/cm^{-1}: 3293, 3071, 2914, 1598, 1467, 1440, 1377, 1294,1242, 1199, 1158, 1100, 1039, 1011, 980, 907.

HRMS (ESI$^+$): *m/z* calcd. for $C_{19}H_{15}BiO_5S_2Na$ $[M+Na]^+$: 619.0057. Found: 619.0056.

MP: 189-190 °C.

4. Synthesis and Characterisation of Guaiacol Substrates

2-Hydroxyphenyl acetate

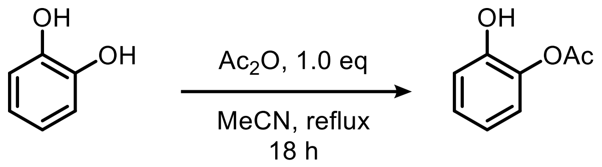

To a stirring solution of catechol (603 mg, 5.48 mmol) in MeCN (4.7 mL) was added Ac_2O (520 µL, 5.48 mmol). The reaction was heated to reflux (aluminium heating block) for 18 h. The reaction was then cooled to rt, quenched with sat. aq. $NaHCO_3$ (5.0 mL), and the organics were extracted with EtOAc (3 × 5 mL). The collected organic portions were dried over sodium sulfate and concentrated *in vacuo*. The crude material was purified *via* automated silica gel column chromatography (0 to 20% Et_2O in cyclohexane) to yield the title compound as a grey crystalline solid (608 mg, 0.71 mmol, 13%). Characterisation data were consistent with literature values: ^{1}H, ^{13}C{^{1}H} NMR.[4]

^{1}H NMR (400 MHz, $CDCl_3$): δ_H 7.14 – 7.05 (2H, m), 6.96 (1H, dd, *J* = 8.1, 1.5 Hz), 6.91 (1H, ddd, *J* = 8.1, 7.3, 1.5 Hz), 5.75 (1H, s), 2.33 (3H, s).

^{13}C{^{1}H} NMR (101 MHz, $CDCl_3$): δ_C 169.8, 147.2, 138.7, 127.2, 122.6, 121.1, 117.8, 21.0.

IR(ATR-IR, neat) υ_{max}/cm^{-1}: 3388, 3020, 2927, 1743, 1596, 1496, 1461, 1370, 1285, 1264, 1214, 1174, 1151, 1097, 1032, 1012, 936, 911.

HRMS (ESI$^+$): *m/z* calcd. for $C_8H_8NaO_3$ $[M+Na]^+$: 175.0371. Found: 175.0361.

MP: 62-64 °C.

Methyl (*E*)-3-(4-hydroxy-3-methoxyphenyl)acrylate

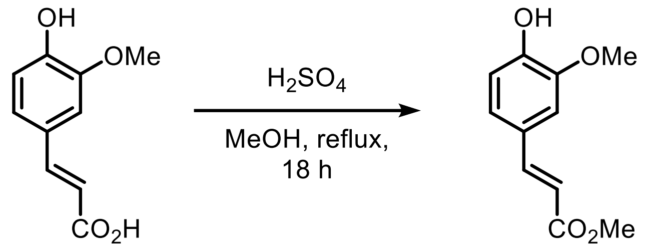

To a solution of (*E*)-ferulic acid (292 mg, 1.50 mmol) in MeOH (1.9 mL) was added conc. H_2SO_4 (0.1 mL), and the reaction was heated to reflux (oil bath) for 18 h. The reaction mixture was then cooled to rt, diluted with DCM (2 mL), washed with sat. aq. $NaHCO_3$ (2 × 2 mL), dried over Na_2SO_4 and concentrated *in vacuo* to afford the crude phenol; this was purified *via* automated silica gel column chromatography (10 to 30% EtOAc in cyclohexane) to yield the title compound as colourless crystals (296 mg, 1.42 mmol, 95%). Characterisation data were consistent with literature values: ^{1}H, ^{13}C{^{1}H} NMR.[5]

^{1}H NMR (400 MHz, $CDCl_3$): δ_H 7.61 (1H, d, *J* = 15.9 Hz), 7.05 (1H, dd, *J* = 8.1, 2.0 Hz), 7.00 (1H, d, *J* = 2.0 Hz), 6.90 (1H, d, *J* = 8.1 Hz), 6.28 (1H, d, *J* = 15.9 Hz), 6.11 (1H, s), 3.89 (3H, s), 3.78 (3H, s).

^{13}C{^{1}H} NMR (101 MHz, $CDCl_3$): δ_C 167.9, 148.1, 146.9, 145.1, 127.0, 123.1, 115.1, 114.9, 109.5, 56.0, 51.7.

IR(ATR-IR, neat) υ_{max}/cm^{-1}: 3391, 3011, 2950, 2843, 1695, 1633, 1590, 1511, 1433, 1376, 1324, 1264, 1156, 1122, 1031, 979, 919.

HRMS (ESI$^-$): *m/z* calcd. for $C_{11}H_{11}O_4$ [M-H]$^-$: 207.0657. Found: 207.0670.

MP: 61-63 °C.

2,3-Dimethoxyphenol

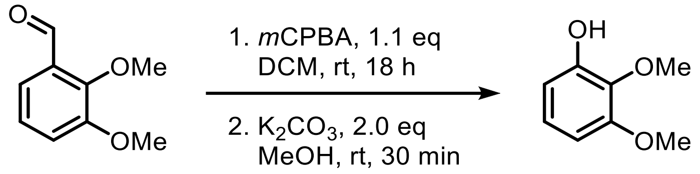

2,3-Dimethoxybenzaldehyde (359 mg, 2.17 mmol) was dissolved in DCM (6 mL) at rt, then *m*CPBA (593 mg, 2.38 mmol, 70% purity) was added in 5 equal portions at one-minute intervals while stirring. The reaction mixture was left to stir at rt for 18 h. The reaction was then quenched with sat. aq. $Na_2S_2O_3$ (3 mL), diluted with DCM (10 mL) and washed subsequently with sat. aq. Na_2SO_3 (1 × 20 mL) and brine (1 × 20 mL). The organic portion was then dried with Na_2SO_4 and concentrated *in vacuo*.

The resultant residue was diluted with MeOH (10 mL), to which K_2CO_3 (598 mg, 4.34 mmol) was added on stirring at rt. After 30 min, the reaction mixture was concentrated *in vacuo*, and suspended in EtOAc (15 mL). The organic portion was washed with H_2O (2 × 10 mL), dried with Na_2SO_4 and concentrated *in vacuo* to afford the crude phenol. This was purified *via* automated silica gel column chromatography (0 to 40% EtOAc in cyclohexane) to yield the title compound as a yellow oil (241 mg, 1.56 mmol, 72%). Characterisation data were consistent with literature values: ^{1}H, ^{13}C{^{1}H} NMR. [6]

^{1}H NMR (400 MHz, $CDCl_3$): δ_H 6.93 (1H, app. t, *J* = 8.3 Hz), 6.62 (1H, dd, *J* = 8.3, 1.4 Hz), 6.48 (1H, dd, *J* = 8.3, 1.4 Hz), 6.04 (1H, s), 3.89 (3H, s), 3.85 (3H, s).

^{13}C{^{1}H} NMR (101 MHz, $CDCl_3$): δ_C 152.6, 149.6, 135.7, 124.1, 108.3, 104.2, 60.9, 55.8.

IR (ATR-IR, neat) υ_{max}/cm^{-1}: 3413, 2941, 2838, 1595, 1497, 1479, 1437, 1344, 1310, 1267, 1232, 1203, 1156, 1086, 996, 946.

HRMS (ESI$^+$): *m*/*z* calcd. for $C_8H_{10}NaO_3$ [M+Na]$^+$: 177.0528. Found: 177.0534.

5. Synthesis and Characterisation of *ortho*-Arylated Phenols

5.1 General Procedure for Dealkoxylative Arylation (GP-3)

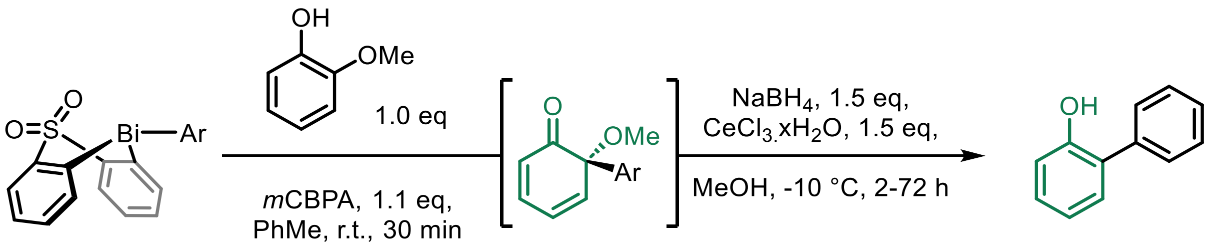

In an amberised RBF, the aryl bismacycle (0.7 mmol) and phenol substrate (0.7 mmol) were suspended in PhMe (35 mL) in the presence of internal standard (diphenyl methane (1.05 mmol) *or* 4,4'-bis(trifluoromethyl)-1,1'-biphenyl (0.117 mmol)) at rt. *m*CPBA (192 mg, 0.77 mmol, 69% purity) was added in 10 equal portions at 30 second intervals while stirring, and the reaction mixture was left for 30 min. The resultant orange solution was quenched with sat. aq. $Na_2S_2O_5$ (0.01 mL), left to stir for 5 min, then passed through a plug of basic alumina (ca. 21 mL). The intermediate cyclohexadienone was eluted with EtOAc (70 mL) and concentrated into a flask containing cerium chloride hydrate (392 mg, 1.05 mmol). This was diluted with MeOH (7.0 mL) and cooled to -10 °C, after which time sodium borohydride (40 mg, 1.05 mmol) was added in 10 equal portions at 40 second intervals while stirring. The reaction was left to warm to rt until no intermediate cyclohexadienone could be observed by TLC. The reaction mixture was then quenched with HCl in dioxane (4 M, 2 mL), left to stir for 5 min, and concentrated *in vacuo* to afford the crude arylated phenol, which was purified by automated silica gel column chromatography using the eluent system described for each compound entry.

5.2 Characterisation Data for *ortho*-Aryl Phenols

4'-Fluoro-[1,1'-biphenyl]-2-ol (5)

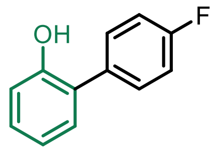

Synthesised according to **GP-3** on a 0.7 mmol scale using guaiacol and 10-(4-fluorophenyl)-10H-dibenzo[b,e][1,4]thiabismine 5,5-dioxide. The crude compound was purified by automated silica gel column chromatography (5-10% Et_2O in pentane) to yield the title compound as a colourless oil (57.3 mg, 0.3 mmol, 44%). Characterisation data were consistent with literature values: ^{1}H, $^{13}C\{^{1}H\}$, ^{19}F NMR and MP.[7]

^{1}H NMR (400 MHz, $CDCl_3$): δ_H 7.50 – 7.40 (2H, m), 7.30 – 7.24 (1H, m), 7.22 (1H, dd, *J* = 7.6, 1.6 Hz), 7.18 (2H, app. tt, *J* = 8.7, 3.1 Hz), 7.00 (1H, dd, *J* = 7.5, 1.1 Hz), 6.97 (1H, dd, *J* = 8.0, 1.1 Hz), 5.01 (1H, s).

^{13}C{^{1}H} NMR (101 MHz, $CDCl_3$): δ_C 162.5 (d, *J* = 247.4 Hz), 152.4, 133.1 (d, *J* = 3.5 Hz), 130.9 (d, *J* = 8.0 Hz), 130.4, 129.3, 127.2, 121.0, 116.1 (d, *J* = 21.4 Hz), 115.9.

^{19}F NMR (377 MHz, $CDCl_3$): δ_F -114.2 (dd, J = 8.7, 5.4 Hz).

IR (ATR-IR, neat) υ_{max}/cm^{-1}: 3541, 3416, 3041, 1901, 1603, 1580, 1514, 1493, 1482, 1452, 1402, 1332, 1286, 1267, 1224, 1181, 1158, 1106, 1094, 1044, 1009, 937.

HRMS (ESI$^-$): *m/z* calcd. for $C_{12}H_8FO$ $[M-H]^-$: 187.0559. Found: 187.0575.

MP: 47-49 °C (lit. 44-45 °C).[7]

4'-Methoxy-[1,1'-biphenyl]-2-ol (6)

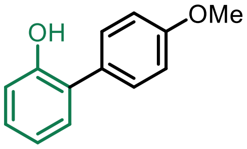

Synthesised according to **GP-3** on a 0.56 mmol scale using guaiacol and 10-(4-methoxyphenyl)-10H-dibenzo[b,e][1,4]thiabismine 5,5-dioxide. The crude compound was purified by automated silica gel column chromatography (5-10% Et_2O in pentane) to yield the title compound as a colourless oil (29.5 mg, 0.15 mmol, 27%). Characterisation data were consistent with literature values: ^{1}H, $^{13}C\{^{1}H\}$.[8]

^{1}H NMR (400 MHz, $CDCl_3$): 7.46 – 7.41 (2H, m), 7.30 – 7.23 (2H, m), 7.08 – 6.98 (4H, m), 5.28 (1H, s), 3.89 (3H, s).

^{13}C{^{1}H} NMR (101 MHz, $CDCl_3$): δ_C 159.3, 152.5, 130.40, 130.38, 129.2, 128.8, 127.9, 120.8, 115.7, 114.7, 55.4.

IR (ATR-IR, neat) υ_{max}/cm^{-1}: 3369, 2924, 2852, 1652, 1607, 1516, 1495, 1483, 1452, 1410, 1336, 1297, 1274, 1246, 1178, 1104, 1034, 1017, 1002.

HRMS (ESI$^-$): *m/z* calcd. for $C_{13}H_{11}O_2$ [M-H]$^-$: 199.0759. Found: 199.0762.

4'-Methyl-[1,1'-biphenyl]-2-ol (7) and 4,4''-dimethyl-[1,1':3',1''-terphenyl]-2'-ol

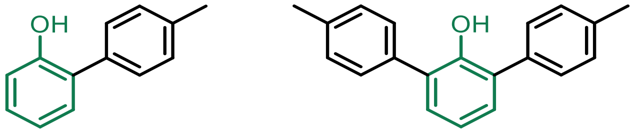

Synthesised according to **GP-3** on a 0.78 mmol scale using guaiacol and 10-(*p*-tolyl)-10H-dibenzo[b,e][1,4]thiabismine 5,5-dioxide. The crude compound was purified by automated silica gel column chromatography (0-10% Et_2O in pentane) to yield 2-(*para*-tolyl)phenol **7** as a yellow oil (46.4 mg, 0.25 mmol, 32%), and 2,6-di(*para*-tolyl)phenol as a colourless solid (6.4 mg, 23.4 µmol, 3%).

4'-Methyl-[1,1'-biphenyl]-2-ol (7)

Characterisation data were consistent with literature values: ^{1}H, ^{13}C{^{1}H} and HRMS.[7]

^{1}H NMR (400 MHz, $CDCl_3$): 7.38 (2H, d, *J* = 8.0 Hz), 7.32 (2H, d, *J* = 8.0 Hz), 7.29 – 7.22 (2H, m), 7.04 – 6.96 (2H, m), 5.29 (1H, s), 2.44 (3H, s).

^{13}C{^{1}H} NMR (MHz, $CDCl_3$): δ_C 152.6, 137.8, 134.2, 130.3, 130.1, 129.1 (2 × C, determined by HSQC), 128.2, 120.9, 115.8, 21.3.

IR (ATR-IR, neat) υ_{max}/cm^{-1}: 3529, 3433, 3024, 2920, 2858, 1703, 1608, 1580, 1517, 1481, 1448, 1404, 1333, 1284, 1267, 1222, 1180, 1152, 1104, 1043, 1024, 1007, 937.

HRMS (ESI$^-$): *m*/*z* calcd. for $C_{13}H_{11}O$ [M-H]$^-$: 183.0815. Found: 183.0824.

4,4''-Dimethyl-[1,1':3',1''-terphenyl]-2'-ol

Characterisation data were consistent with literature values: ^{1}H, ^{13}C{^{1}H}.[9]

^{1}H NMR (500 MHz, $CDCl_3$): δ_H 7.44 (4H, app. dt, *J* = 8.1, 1.8 Hz), 7.28 (4H, d, *J* = 8.1 Hz), 7.25 (2H, d, *J* = 7.6 Hz), 7.03 (1H, t, *J* = 7.6 Hz), 5.41 (1H, s), 2.41 (6H, s).

^{13}C{^{1}H} NMR (126 MHz, $CDCl_3$): δ_C 149.6, 137.5, 134.8, 129.9, 129.7, 129.3, 128.8, 120.7, 21.4.

IR (ATR-IR, neat) υ_{max}/cm^{-1}: 3543, 3024, 2953, 2922, 2852, 1739, 1588, 1515, 1445, 1397, 1377, 1326, 1246, 1224, 1184, 1166, 1097, 1077, 1021.

HRMS (ESI$^-$): *m*/*z* calcd. for $C_{20}H_{17}O$ [M-H]$^-$: 273.1284. Found: 273.1295.

MP: 78-81 °C.

3'-Ethoxy-[1,1'-biphenyl]-2-ol (8)

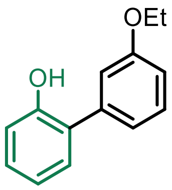

Synthesised according to **GP-3** on a 0.7 mmol scale using guaiacol and 10-(3-ethoxyphenyl)-10*H*-dibenzo[*b*,*e*][1,4]thiabismine 5,5-dioxide. The crude compound was purified by automated silica gel column chromatography (0 to 4% TBME in pentane) to yield the title compound an orange oil (77.7 mg, 0.32 mmol, 46%).

^{1}H NMR ($CDCl_3$, 500 MHz): δ_H 7.40 (1H, app. t, *J* = 7.9 Hz), 7.31 – 7.23 (2H, m), 7.0 (1H, dd, *J* = 7.6), 7.01 – 6.97 (3H, m), 6.94 (1H, ddd, *J* = 8.3, 2.6, 1.0 Hz), 5.38 (1H, s), 4.08 (2H, q, *J* = 7.0 Hz), 1.45 (3H, t, *J* = 7.0 Hz)

^{13}C{^{1}H} NMR (126 MHz, $CDCl_3$): δ_C 159.7, 152.5, 138.4, 130.4, 130.1, 129.2, 128.0, 121.1, 120.8, 115.8, 115.1, 114.2, 63.6, 14.9.

IR (ATR-IR, neat) υ_{max}/cm^{-1}: 3018, 1599, 1474, 1412, 1214, 1025, 933.

HRMS (ESI$^-$): *m/z* calcd. for $C_{14}H_{13}O_2$ [M-H]$^-$: 213.0916. Found: 213.0923.

3'-Bromo-[1,1'-biphenyl]-2-ol (9)

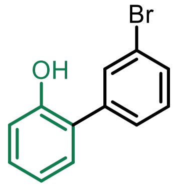

Synthesised according to **GP-3** on a 0.7 mmol scale using guaiacol and 10-(3-bromophenyl)-10H-dibenzo[b,e][1,4]thiabismine 5,5-dioxide. The crude compound was purified by automated silica gel column chromatography (0 to 5% Et_2O in pentane) to yield the title compound as colourless solid (68.4 mg, 0.27 mmol, 39%).

^{1}H NMR ($CDCl_3$, 400 MHz): δ_H 7.67 (1H, app. t, *J* = 1.8 Hz), 7.53 (1H, ddd, *J* = 7.9, 1.8, 1.5 Hz), 7.43 (1H, app. dt, *J* = 7.9, 1.3 Hz), 7.34 (1H, app. t, *J* = 7.9 Hz), 7.28 (1H, app. td, *J* = 8.2, 1.8 Hz), 7.24 (1H, dd, *J* = 7.6, 1.8 Hz), 7.01 (1H, app. td, *J* = 7.6, 1.2 Hz), 6.96 (1H, dd, *J* = 8.2, 1.2 Hz), 5.15 (1H, s).

^{13}C{^{1}H} NMR (101 MHz, $CDCl_3$): δ_C 152.4, 139.5, 132.3, 130.9, 130.6, 130.4, 129.7, 127.8, 126.9, 123.2, 121.2, 116.2.

IR (ATR-IR, neat) υ_{max}/cm^{-1}: 3018, 1724, 1593, 1515, 1409, 1214, 1091.

HRMS (ESI$^-$): *m/z* calcd. for $C_{12}H_8BrO$ [M-H]$^-$: 246.9759. Found: 246.9766.

MP: 75-78 °C.

4'-(Trifluoromethoxy)-[1,1'-biphenyl]-2-ol (10)

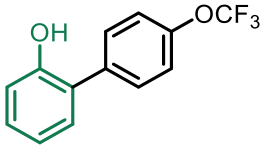

Synthesised according to **GP-3** on a 0.7 mmol scale using guaiacol and 10-(4-(trifluoromethoxy)phenyl)-10*H*-dibenzo[*b*,*e*][1,4]thiabismine 5,5-dioxide. The crude compound was purified by automated silica gel column chromatography (0 to 3% TBME in pentane) to yield the title compound as a colourless solid (88.0 mg, 0.35 mmol, 50%). Characterisation data were consistent with literature values: ^{1}H, ^{13}C{^{1}H} and HRMS.[7]

^{1}H NMR ($CDCl_3$, 500 MHz): δ_H 7.52 (2H, d, *J* = 8.3 Hz), 7.33 (2H, d, *J* = 8.3 Hz), 7.28 (1H, dd, *J* = 7.8, 1.7 Hz), 7.24 (1H, dd, *J* = 7.8, 1.7 Hz), 7.01 (1H, app. td, *J* = 7.8, 1.2 Hz), 6.97 (1H, dd, *J* = 7.8, 1.2 Hz), 5.00 (1H, s)

^{13}C{^{1}H} NMR (126 MHz, $CDCl_3$): δ_C 152.5, 148.9 (q, *J* = 1.70 Hz), 136.1, 130.8, 130.5, 129.6, 127.0, 121.6, 121.3, 120.7 (q, *J* = 257.4 Hz), 116.2

^{19}F NMR (377 MHz, $CDCl_3$): δ_F -57.76 (s)

IR (ATR-IR, neat) υ_{max}/cm^{-1}: 3525, 3432, 2925, 2855, 1703, 1603, 1592, 1512, 1493, 1452, 1405, 1254, 1207, 1159, 1101, 1044, 1021, 1007, 939, 920.

HRMS (ESI^{-}): *m/z* calcd. for $C_{13}H_8F_3O_2$ $[M-H]^-$: 253.0476. Found: 253.0488.

MP: 64-67 °C.

4'-(Trifluoromethyl)-[1,1'-biphenyl]-2-ol (11)

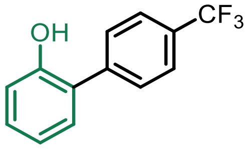

Synthesised according to **GP-3** on a 0.70 mmol scale using guaiacol and 10-(4-(trifluoromethyl)phenyl)-10H-dibenzo[b,e][1,4]thiabismine 5,5-dioxide. The crude compound was purified by automated silica gel column chromatography (5-10% Et_2O in pentane) to yield the title compound as a colourless solid (100 mg, 0.42 mmol, 60%). Characterisation data were consistent with literature values: ^{1}H, ^{13}C{^{1}H}, ^{19}F NMR, HRMS and MP.[7]

^{1}H NMR (400 MHz, $CDCl_3$): δ_H 7.77 – 7.70 (2H, m), 7.67 – 7.61 (2H, m), 7.34 – 7.26 (2H, m), 7.04 (1H, app. td, *J* = 7.5, 1.2 Hz), 6.97 (1H, ddd, *J* = 8.0, 1.2, 0.3 Hz), 5.07 (1H, s)

^{13}C{^{1}H} NMR (126 MHz, $CDCl_3$): δ_C 152.4, 141.2, 130.4, 129.8, 129.7 (q, *J* = 32.0 Hz), 129.6, 126.9, 125.9 (q, *J* = 3.8 Hz), 124.2 (q, *J* = 272.0 Hz), 121.3, 116.2.

^{19}F NMR (376 MHz, $CDCl_3$): δ_F -62.5.

IR (ATR-IR, neat) υ_{max}/cm^{-1}: 3522, 3033, 2923, 2852, 1615, 1605, 1588, 1523, 1495, 1450, 1404, 1326, 1296, 1273, 1163, 1129, 1112, 1100, 1069, 1044, 1021, 1008, 955, 945.

HRMS (ESI$^-$): *m/z* calcd. for $C_{13}H_8F_3O$ [M-H]$^-$: 237.0533. Found: 237.0534.

MP: 110-112 °C.

2'-Hydroxy-[1,1'-biphenyl]-4-carbonitrile (12) and dienol (12e)

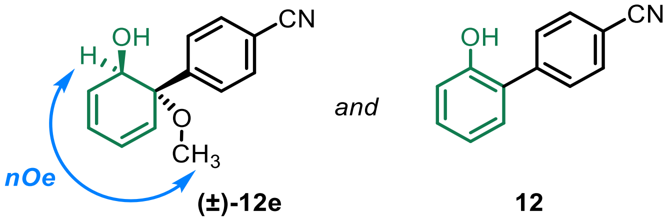

Synthesised according to a modified version of **GP-3** on a 0.716 mmol scale using guaiacol and 4-(5,5-dioxido-10H-dibenzo[b,e][1,4]thiabismin-10-yl)benzonitrile. The modification follows from the Luche reduction, as detailed below. Dienol **12e** was formed in *ca* 44% yield, as determined by ^{1}H NMR spectroscopic analysis (*vs* 1,3,5-trimethoxybenzene).

The reaction mixture was then quenched with aq. HCl (2 M, 7.0 mL), left to stir for 5 min, then extracted with Et_2O (3 × 10 mL). The combined organic portions were washed once with brine (10 mL), dried with anhydrous sodium sulfate, and concentrated *in vacuo* to afford the crude arylated phenol, which was purified by automated silica gel column chromatography (5-10% Et_2O in pentane) to yield title compound **12** as off-white crystals (36.6 mg, 0.19 mmol, 26%). Characterisation data were consistent with literature values: ^{1}H, ^{13}C{^{1}H}.[10]

The column was flushed with isopropanol and the eluent was concentrated under reduced pressure. Crude dienol **12e** was obtained from the resulting residue in sufficient purity for structure determination following preparative thin layer chromatography (10 % Et_2O in a 1% solution of Et_3N in pentane); full purification could not be achieved due to the modest stability of dienol **12e**; its relative stereochemistry was determined by NOESY.

Characterisation data for **12e**:

^{1}H NMR (400 MHz, $CDCl_3$): δ_H 7.67 (2H, d, *J* = 8.2 Hz), 7.61 (2H, d, *J* = 8.2 Hz), 6.38 (1H, dd, *J* = 9.9, 5.1 Hz), 6.07 (1H, dd, *J* = 9.7, 5.1 Hz), 6.01 (1H, d, *J* = 9.9 Hz), 5.96 (1H, dd, *J* = 9.7, 3.8 Hz), 4.59-4.42 (1H, m), 3.25 (3H, s), 1.39 (1H, d, *J* = 6.7 Hz).

^{13}C{^{1}H} NMR (101 MHz, $CDCl_3$): δ_C 144.0, 132.0, 130.4, 128.6, 128.1 (2 × C, determined by HSQC), 123.8, 119.0, 111.9, 81.8, 72.1, 52.4.

NMR Spectra for dienol 12e:

^{1}H NMR (400 MHz, $CDCl_3$):

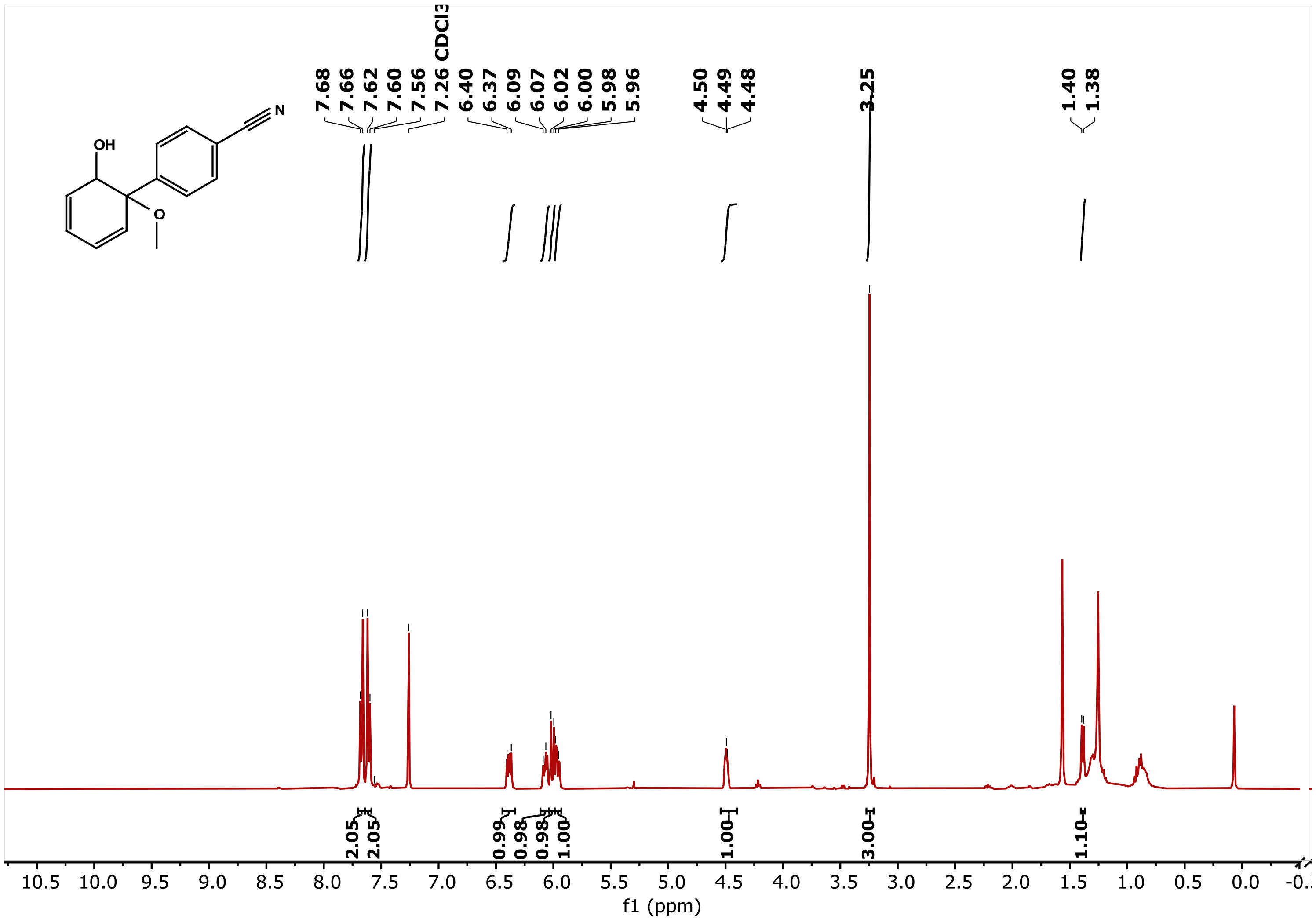

^{13}C{^{1}H} NMR (101 MHz, $CDCl_3$):

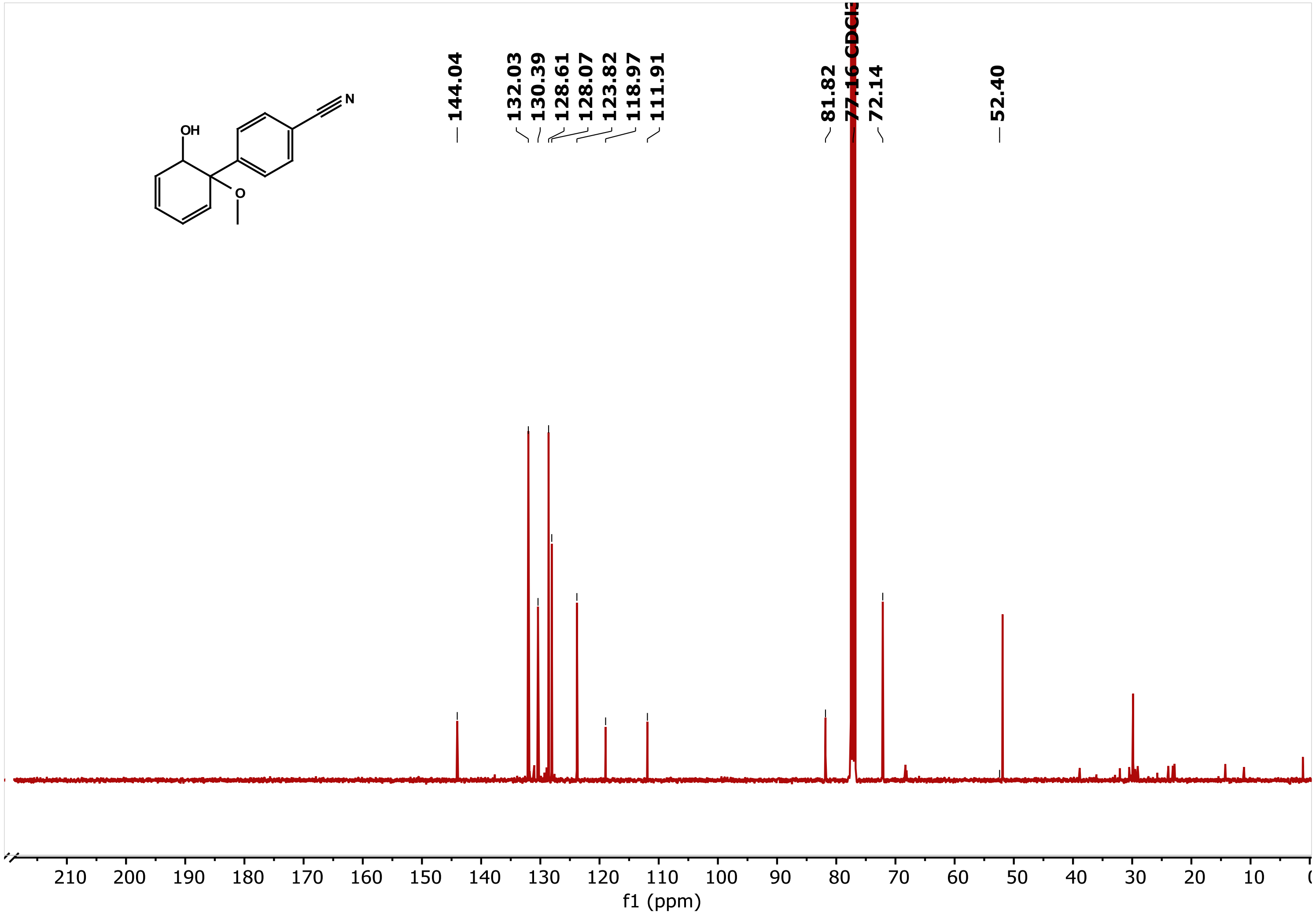

Aryl phenol **12** was also synthesised according to **GP-3** on a 0.55 mmol scale using guaiacol and 4-(5,5-dioxido-10H-dibenzo[b,e][1,4]thiabismin-10-yl)benzonitrile. The crude compound was purified by automated silica gel column chromatography (0-20% Et_2O in pentane) to yield the title compound (87 mg, 0.44 mmol, 81%).

^{1}H NMR (400 MHz, $CDCl_3$): δ_H 7.73 (2H d, *J* = 8.3 Hz), 7.66 (2H, d, *J* = 8.3 Hz), 7.34 – 7.32 (2H, m), 7.04 (1H dd, *J* = 7.6 Hz, 1.1 Hz), 6.94 (1H, d, *J* = 8.0 Hz), 5.24 (1H, s).

^{13}C{^{1}H} NMR (101 MHz, $CDCl_3$): δ_C 152.6, 142.8, 132.6, 130.6, 130.2, 130.1, 126.7, 121.5, 119.0, 116.5, 111.1.

IR (ATR-IR, neat) υ_{max}/cm^{-1}: 3355, 2231, 1604, 1492, 1450, 1401, 1356, 1294, 1274, 1205, 1180, 1106, 909.

HRMS (ESI$^-$): *m/z* calcd. for $C_{13}H_9NO$ [M-H]$^-$: 194.0611. Found: 194.0612.

MP: 116-119 °C.

2-(Naphthalen-1-yl)phenol (13)

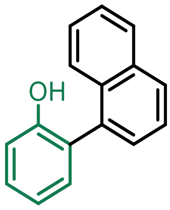

Synthesised according to **GP-3** on a 0.53 mmol scale using guaiacol and 10-(naphthalen-1-yl)-10H-dibenzo[b,e][1,4]thiabismine 5,5-dioxide. The crude compound was purified by automated silica gel column chromatography (0-10% Et_2O in pentane) to yield the title compound as a colourless oil (13 mg, 0.06 mmol, 11%). Characterisation data were consistent with literature values: ^1H, $^{13}C\{^1H\}$.[11]

^{1}H NMR (400 MHz, $CDCl_3$): δ_H 8.00-7.93 (2H, m), 7.67 (1H, d, *J* = 8.6 Hz), 7.58 (1H, dd, *J* = 8.3 Hz), 7.56 – 7.44 (3H, m), 7.41 – 7.35 (1H, m), 7.31 – 7.25 (1H, m), 7.11 – 7.01 (2H, m), 4.80 (1H, s).

$^{13}C\{^1H\}$ NMR (101 MHz, $CDCl_3$): δ_C 153.3, (134.1 2 × C, determined by HSQC), 132.0, 131.4, 129.7, 129.0, 128.6, 128.4, 126.9, 126.5, 126.4, 125.9, 125.8, 120.7, 115.7.

IR (ATR-IR, neat) υ_{max}/cm^{-1}: 3019, 2324, 2036, 1944, 1562, 1524, 1499, 1438, 1303, 1286, 1252, 1214, 1150, 1123, 1087, 1073, 1012, 915.

HRMS (ESI$^-$): *m/z* calcd. for $C_{16}H_{11}O$ [M-H]$^-$: 219.0815. Found: 219.0817.

2-(6-Methoxypyridin-3-yl)phenol (14)

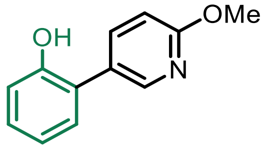

Synthesised according to a **GP-3** on a 0.7 mmol scale using guaiacol and 10-(6-methoxypyridin-3-yl)-10*H*-dibenzo[*b*,*e*][1,4]thiabismine 5,5-dioxide. Synthesis features an alternative work-up following the Luche reduction: The reaction mixture was quenched with HCl (4.0 M; 7 mL) and stirred for 10 min. Following neutralisation with K_2CO_3, the phenol was extracted with Et_2O (3 × 10 mL) and the collected organic portions were dried over sodium sulfate before the solvent was removed *in vacuo*. The resultant crude compound was purified by automated silica gel column chromatography (3 % Et_2O in pentane) to yield the title compound as a white solid (32.8 mg, 0.16 mmol, 24%).

^{1}H NMR (DMSO, 400 MHz): δ_H 9.64 (1H, s), 8.33 (1H, d, *J* = 2.4 Hz), 7.90 (1H, ddd, *J* = 8.5, 2.4, 1.1 Hz), 7.27 (1H, d, *J* = 7.5 Hz), 7.22 – 7.11 (1H, m), 7.96 (1H, d, *J* = 8.2 Hz), 6.87 (1H, d, *J* = 7.5 Hz), 6.85 (1H, d, *J* = 8.5 Hz), 3.88 (3H, d, *J* = 1.1 Hz).

^{13}C{^{1}H} NMR (DMSO, 101 MHz): δ_C 162.3, 154.4, 146.4, 139.8, 130.0, 128.7, 127.7, 124.3, 119.6, 116.0, 109.6, 53.1.

IR (ATR-IR, neat) υ_{max}/cm^{-1}: 3018, 1603, 1514, 1487, 1409, 1289, 1214, 1023.

HRMS (ESI$^-$): *m/z* calcd. for $C_{12}H_{10}NO_2$ [M-H]$^-$: 200.0712. Found: 200.0721.

MP: decomp. at 240 °C.

4'-Fluoro-3-methoxy-[1,1'-biphenyl]-2-ol (16)

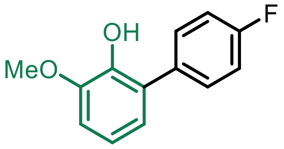

Synthesised according to **GP-3** on a 0.7 mmol scale using 2,6-dimethoxyphenol and 10-(4-fluorophenyl)-10H-dibenzo[b,e][1,4]thiabismine 5,5-dioxide. The crude compound was purified by automated silica gel column chromatography (5-10% Et_2O in pentane) to yield the title compound as a yellow powder (64 mg, 0.29 mmol, 42%).

^{1}H NMR (400 MHz, $CDCl_3$): δ_H 7.61 (2H, dd, J = 8.8, 5.5 Hz), 7.13 (2H, appt. tt, J = 8.8, 2.9 Hz), 7.00 – 6.84 (3H, m), 5.92 (1H, s), 3.95 (3H, s).

^{13}C{^{1}H} NMR (101 MHz, $CDCl_3$): δ_C 162.1 (d, J = 245.9 Hz), 146.9, 142.8, 133.7 (d, J = 3.3 Hz), 130.8 (d, J = 7.9 Hz), 126.7, 122.6, 119.8, 115.1 (d, J = 21.4 Hz), 109.7, 56.2.

^{19}F NMR (377 MHz, $CDCl_3$): δ_F -115.6 (tt, J = 8.8, 5.5 Hz).

IR (ATR-IR, neat) υ_{max}/cm^{-1}: 3520, 3015, 2937, 2847, 1601, 1584, 1512, 1472, 1441, 1401, 1361, 1296, 1267, 1216, 1186, 1158, 1109, 1077, 1026, 1014, 946.

HRMS (ESI$^-$): *m/z* calcd. for $C_{13}H_{10}FO_2$ $[M-H]^-$: 217.0665. Found: 217.0674.

MP: 88-90 °C.

4'-Fluoro-3-methyl-[1,1'-biphenyl]-2-ol (17)

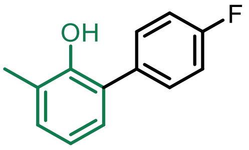

Synthesised according to **GP-3** on a 0.7 mmol scale using 2-methoxy-6-methylphenol and 10-(4-fluorophenyl)-10H-dibenzo[b,e][1,4]thiabismine 5,5-dioxide. The crude compound was purified by automated silica gel column chromatography (0 to 20% Et_2O in pentane) to yield the title compound as a colourless oil (23 mg, 0.11 mmol, 16%).

^{1}H NMR (400 MHz, $CDCl_3$): δ_H 7.44 (2H, dd, *J* = 8.8, 5.4 Hz), 7.11 – 7.01 (3H, m), 7.06 (1H, ddd, *J* = 7.6, 1.8, 0.6 Hz), 6.91 (1H, d, *J* = 7.6 Hz), 5.09 (1H, s), 2.32 (3H, s).

^{13}C{^{1}H} NMR ($CDCl_3$, 101 MHz) δ_C 162.6 (d, *J* = 247.3 Hz), 150.7, 133.4 (d, *J* = 3.5 Hz), 131.1 (d, *J* = 8.0 Hz), 130.7, 128.0, 126.9, 124.8, 120.5, 116.3 (d, *J* = 21.4 Hz), 16.3.

^{19}F NMR (376 MHz, $CDCl_3$): δ_F -114.2 (tt, *J* = 8.6, 5.4 Hz).

IR (ATR-IR, neat) υ_{max}/cm^{-1}: 3502, 3488, 3385, 3336, 3301, 2981, 2925, 1597, 1510, 1473, 1420, 1401, 1215, 1159, 1086, 1024, 935.

HRMS (ESI$^-$): *m/z* calcd. for $C_{13}H_{10}FO$ [M-H]$^-$: 201.0716. Found: 201.0724.

1,7-Dibromo-5,9-bis(4-fluorophenyl)-5,9-dimethoxy-1,4a,5,8a-tetrahydro-1,4-ethanonaphthalene-6,10(4H)-dione (18a)₂

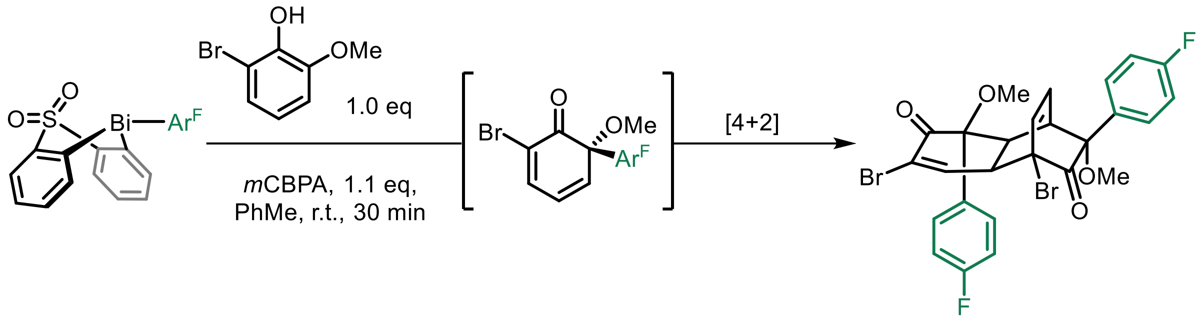

In an amberised RBF, 10-(4-fluorophenyl)-10H-dibenzo[b,e][1,4]thiabismine 5,5-dioxide (83.0 mg, 0.16 mmol) **2a** and 6- bromo guaiacol (33.1 mg, 0.16 mmol) were suspended in PhMe (8 mL) in the presence of internal standard 4,4'-bis(trifluoromethyl)-1,1'-biphenyl (5.00 mg, 0.02 mmol) at rt. *m*CPBA (44 mg, 0.18 mmol, 69% purity) was added in 10 equal portions at 30 second intervals while stirring, and the reaction mixture was left for 30 min. The resultant orange solution was quenched with sat. aq. $Na_2S_2O_5$ (0.01 mL), left to stir for 5 min, then passed through a plug of basic alumina (*ca* 21 mL). The solution was eluted with EtOAc (70 mL) and concentrated *in vacuo*. Analysis of the crude material by ^{19}F NMR spectroscopy indicated that none of the expected cyclohexadienone remained, and that the corresponding dimer was formed in 68% yield. The crude material was purified by automated silica gel column chromatography (0 to 5% EtOAc in cyclohexane) to yield the title compound as a yellow solid (10 mg, 16.8 μmol, 21%).

Observed as single diastereoisomer; relative stereochemistry could not be determined unambiguously.

^{1}H NMR (400 MHz, $CDCl_3$): δ_H 7.31 – 7.26 (2H, m), 7.20 (2H, dd, *J* = 8.5, 5.1 Hz), 7.11 (2H, app. t, *J* = 8.3 Hz), 7.03 (2H, app. t, *J* = 8.4 Hz), 6.90 (1H, d, *J* = 4.3 Hz), 5.98 (1H, s), 5.96 (1H, d, *J* = 1.8 Hz), 4.33 (1H, d, *J* = 8.3 Hz), 3.82 – 3.76 (1H, m), 3.54 (1H, dd, *J* = 8.3, 4.3 Hz), 3.24 (3H, s), 3.08 (3H, s).

^{13}C{^{1}H} NMR ($CDCl_3$, 101 MHz) δ_C 199.6, 191.5, 164.2 (d, *J* = 24.0 Hz), 161.7 (d, *J* = 23.0 Hz), 141.9, 135.3, 134.3, 133.3 (d, *J* = 3.4 Hz), 133.3 (d, *J* = 3.5 Hz), 130.0 (d, *J* = 8.2 Hz), 129.4 (d, *J* = 8.3 Hz), 124.0, 116.4 (d, *J* = 21.7 Hz), 115.7 (d, *J* = 21.6 Hz), 83.1, 79.8, 71.7, 53.4, 52.1, 49.6, 43.9, 39.7.

^{19}F NMR (376 MHz, $CDCl_3$): δ_F -111.4 (tt, *J* = 8.4, 5.1 Hz), -112.3 (tt, *J* = 8.4, 5.1 Hz).

IR (ATR-IR, neat) υ_{max}/cm^{-1}: 3068, 2935, 2835, 1717, 1508, 1236, 1163, 1106, 1090.

HRMS (ESI$^+$): *m/z* calcd. for $C_{26}H_{20}Br_2F_2NaO_4$ [M+Na]$^+$: 616.9574. Found: 616.9582.

MP: 189-193 °C.

4'-Fluoro-4-methyl-[1,1'-biphenyl]-2-ol (19)

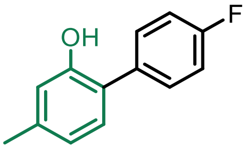

Synthesised according to **GP-3** on a 0.7 mmol scale using 2-methoxy-5-methylphenol and 10-(4-fluorophenyl)-10H-dibenzo[b,e][1,4]thiabismine 5,5-dioxide. The crude compound was purified by automated silica gel column chromatography (5-10% Et_2O in pentane) to yield the title compound as colourless solid (87.6 mg, 0.43 mmol, 62%). Characterisation data were consistent with literature values: ^1H, $^{13}C\{^1H\}$.[12]

^{1}H NMR (400 MHz, $CDCl_3$): δ_H 7.44 (2H, dd, J = 8.6, 5.6 Hz), 7.16 (2H, app. t, J = 8.6 Hz), 7.12 (1H, d, J = 7.8 Hz), 6.82 (1H, d, J = 7.8 Hz), 6.80 (1H, s), 5.00 (1H, s), 2.36 (3H, s).

^{13}C{^{1}H} NMR (126 MHz, $CDCl_3$): δ_C 162.5 (d, J = 247.1 Hz), 152.3, 139.6, 133.3 (d, J = 3.2 Hz), 131.0 (d, J = 7.8 Hz), 130.2, 124.5, 121.9, 116.6, 116.2 (d, J = 21.4 Hz), 21.3.

^{19}F NMR (377 MHz, $CDCl_3$): δ_F -114.6 (tt, J = 8.6, 5.6 Hz).

IR (ATR-IR, neat) υ_{max}/cm^{-1}: 3534, 3426, 3036, 2921, 2858, 1703, 1620, 1602, 1569, 1517, 1496, 1452, 1423, 1397, 1287, 1222, 1189, 1157, 1121, 1095, 1038, 1009, 943, 908.

HRMS (ESI$^-$): *m/z* calcd. for $C_{13}H_{10}FO$ [M-H]$^-$: 201.0721. Found: 201.0722.

MP: 54-60 °C.

4-Bromo-4'-fluoro-[1,1'-biphenyl]-2-ol (20)

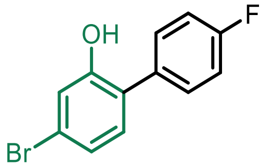

Synthesised according to **GP-3** on a 0.7 mmol scale using methyl 5-bromo guaiacol and 10-(4-fluorophenyl)-10H-dibenzo[b,e][1,4]thiabismine 5,5-dioxide. The crude compound was purified by automated silica gel column chromatography (5 to 10 % Et_2O in cyclohexane) to yield the title compound as colourless oil (55.0 mg, 0.21 mmol, 30%). ^1H, $^{13}C\{^1H\}$,^{19}F NMR and HRMS.[2]

^{1}H NMR (500 MHz, $CDCl_3$): δ_H 7.41 (2H, dd, J = 8.5, 5.2 Hz), 7.18 (2H, app. t, J = 8.5 Hz), 7.15 – 7.12 (2H, m), 7.08 (1H, d, J = 8.0 Hz), 5.14 (1H, s).

^{13}C{^{1}H} NMR ($CDCl_3$, 126 MHz) δ_C 162.7 (d, J = 248.1 Hz), 153.2, 132.1 (d, J = 3.3 Hz), 131.5, 130.9 (d, J = 8.1 Hz), 126.4, 124.3, 122.3, 119.3, 116.5 (d, J = 21.5 Hz).

^{19}F NMR (377 MHz, $CDCl_3$): δ_F -113.36 (tt, J = 8.5, 5.2 Hz).

IR (ATR-IR, neat) υ_{max}/cm^{-1}: 3064, 2196, 1842, 1596, 1514, 1490, 1417, 1322, 1221, 1145.

HRMS (ESI$^-$): *m/z* calcd. for $C_{12}H_8BrFO$ [M-H]$^-$: 264.9670. Found: 264.9674.

4'-Fluoro-5-methyl-[1,1'-biphenyl]-2-ol (21)

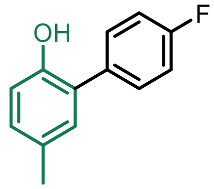

Synthesised according to **GP-3** on a 0.7 mmol scale using 2-methoxy-4-methylphenol and 10-(4-fluorophenyl)-10H-dibenzo[b,e][1,4]thiabismine 5,5-dioxide. The crude compound was purified by automated silica gel column chromatography (5-10% Et_2O in pentane) to yield the title compound as a brown oil (70 mg, 0.35 mmol, 50%).

^{1}H NMR (400 MHz, $CDCl_3$): δ_H 7.51 – 7.34 (2H, m), 7.17 (2H, app. t, *J* = 8.7 Hz), 7.07 (1H, dd, *J* = 8.1, 2.1 Hz), 7.04 (1H, d, *J* = 2.1 Hz), 6.87 (1H, d, *J* = 8.1 Hz), 4.96 (1H, s), 2.33 (3H, s)

^{13}C{^{1}H} NMR (101 MHz, $CDCl_3$): δ_C 162.5 (d, *J* = 247.1 Hz), 150.2, 133.4 (d, *J* = 3.3 Hz), 131.0, 130.9, 130.3, 129.8, 127.1, 116.1 (d, *J* = 21.4 Hz), 115.9, 20.6.

^{19}F NMR (377 MHz, $CDCl_3$): δ_F -114.4 (tt, *J* = 8.7, 5.4 Hz).

IR (ATR-IR, neat) υ_{max}/cm^{-1}: 3539, 3416, 3019, 2921, 2861, 1603, 1494, 1463, 1394, 1329, 1299, 1267, 1222, 1158, 1127, 1094, 1038, 1015, 942, 909.

HRMS (ESI$^-$): *m/z* calcd. for $C_{13}H_{10}FO$ [M-H]$^-$: 201.0721. Found: 201.0727.

5-Bromo-4'-fluoro-[1,1'-biphenyl]-2-ol (22)

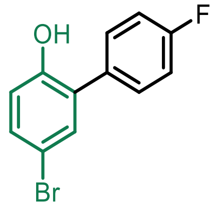

Synthesised according to **GP-3** on a 0.7 mmol scale using 4-bromo-2-methoxyphenol and 10-(4-fluorophenyl)-10H-dibenzo[b,e][1,4]thiabismine 5,5-dioxide. The crude compound was purified by automated silica gel column chromatography (0 to 5% Et_2O in pentane) to yield the title compound as colourless oil (60 mg, 0.23 mmol, 32%). ^{1}H, ^{13}C{^{1}H},^{19}F NMR and HRMS.[2]

^{1}H NMR (400 MHz, $CDCl_3$): δ_H 7.42 (2H, dd, *J* = 8.7, 5.3 Hz), 7.36 – 7.31 (2H, m), 7.18 (2H, app t, *J* = 8.7 Hz), 6.88 – 6.81 (1H, m), 5.12 (1H, s).

^{13}C{^{1}H} NMR ($CDCl_3$, 101 MHz) δ_C 162.8 (d, *J* = 248.5 Hz), 151.7, 132.9, 132.0, 131.9 (d, *J* = 3.4 Hz), 131.0 (d, *J* = 8.1 Hz), 129.3, 117.9, 116.4 (d, *J* = 21.6 Hz), 113.0.

^{19}F NMR (376 MHz, $CDCl_3$): δ_F -113.10 (tt, *J* = 8.7, 5.3 Hz).

IR (ATR-IR, neat) υ_{max}/cm^{-1}: 3536, 3406, 2925, 1599, 1511, 1487, 1477, 1419, 1388, 1324, 1301, 1259, 1224, 1158, 1123, 1095, 1023, 1014.

HRMS (ESI$^-$): *m/z* calcd. for $C_{12}H_7BrFO$ [M-H]$^-$: 264.9664. Found: 264.9674.

Methyl (*E*)-3-(4'-fluoro-6-hydroxy-[1,1'-biphenyl]-3-yl)acrylate (23)

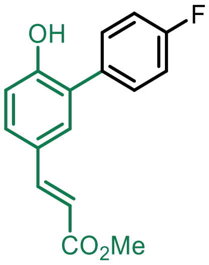

Synthesised according to **GP-3** on a 0.6 mmol scale using methyl (*E*)-3-(4-hydroxy-3-methoxyphenyl)acrylate and 10-(4-fluorophenyl)-10H-dibenzo[b,e][1,4]thiabismine 5,5-dioxide. The crude compound was purified by automated silica gel column chromatography (0 to 20 % Et_2O in pentane) to yield the title compound as colourless solid (45.0 mg, 0.17 mmol, 28%).

^{1}H NMR (400 MHz, $CDCl_3$): δ_H 7.66 (1H, d, *J* = 16.0 Hz), 7.49 – 7.37 (4H, m), 7.23 – 7.14 (2H, m), 6.99 (1H, d, *J* = 8.5 Hz), 6.33 (1H, d, *J* = 16.0 Hz), 5.91 (1H, s), 3.80 (3H, s).

^{13}C{^{1}H} NMR ($CDCl_3$, 101 MHz) δ_C 168.1, 162.7 (d, *J* = 247.9 Hz), 154.9, 144.7, 132.5 (d, *J* = 3.4 Hz), 131.0 (d, *J* = 8.1 Hz), 130.7, 129.3, 128.0, 127.5, 116.7, 116.2 (d, *J* = 21.4 Hz), 115.6, 51.9.

^{19}F NMR (376 MHz, $CDCl_3$): δ_F -113.58 (tt, *J* = 8.5, 5.3 Hz).

IR (ATR-IR, neat) υ_{max}/cm^{-1}: 3018, 2925, 1599, 1475, 1420, 1214, 1025, 934.

HRMS (ESI$^-$): *m/z* calcd. for $C_{16}H_{12}FO_3$ [M-H]$^-$: 271.0770. Found: 271.0776.

MP: 98-90 °C.

4'-Fluoro-6-methoxy-[1,1'-biphenyl]-2-ol (24) and '' enone side product

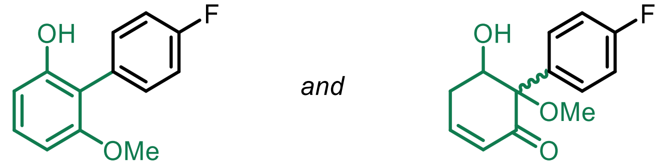

24 *side product*

Synthesised according to **GP-3** on a 0.7 mmol scale using 2,3-dimethoxyphenol and 10-(4-fluorophenyl)-10H-dibenzo[b,e][1,4]thiabismine 5,5-dioxide. The crude compound was purified by automated silica gel column chromatography (5 % Et_2O in pentane) to yield aryl phenol **24** as an off white solid (38 mg, 0.17 mmol, 25%) and the side product as a yellow oil (32% yield, determined by ^{19}F NMR spectroscopic analysis prior to purification).

4'-Fluoro-6-methoxy-[1,1'-biphenyl]-2-ol (**24**):

^{1}H NMR (400 MHz, $CDCl_3$): δ_H 7.35 (2H, dd, *J* = 8.6, 5.4 Hz), 7.28 – 7.14 (3H, m), 6.66 (1H, dd, *J* = 8.3, 1.0 Hz), 6.58 (1H, dd, *J* = 8.3, 0.9 Hz), 4.93 (1H, s), 3.74 (3H, s).

^{13}C{^{1}H} NMR ($CDCl_3$, 101 MHz) δ_C 162.6 (d, *J* = 247.2 Hz), 157.6, 153.8, 132.7 (d, *J* = 8.0 Hz), 129.4, 128.3 (d, *J* = 3.3 Hz), 116.3 (d, *J* = 21.4 Hz), 116.3, 108.6, 103.3, 55.9.

^{19}F NMR (377 MHz, $CDCl_3$): δ_F -113.79 (tt, *J* = 8.6, 5.4 Hz).

IR (ATR-IR, neat) υ_{max}/cm^{-1}: 3550, 3432, 3049, 3010, 2937, 2838, 1597, 1584, 1513, 1466, 1438, 1401, 1323, 1306, 1253, 1221, 1197, 1170, 1156, 1077, 1008, 94.

HRMS (ESI$^-$): *m/z* calcd. for $C_{13}H_{10}FO_2$ [M-H]$^-$: 217.0665. Found: 217.0673.

MP: 106-109 °C.

Enone side-product:

^{1}H NMR (400 MHz, $CDCl_3$): δ_H 7.46 (2H, dd, *J* = 8.6, 5.4 Hz), 7.10 (2H, d, *J* = 8.6 Hz), 6.87 (1H, dt, *J* = 10.2, 4.2 Hz), 6.20 (1H, dt, *J* = 10.2, 2.0 Hz), 4.23 (1H, dd, *J* = 6.5, 4.2 Hz), 3.30 (3H, s), 2.86 (1H, dtd, *J* = 18.8, 4.2, 2.0 Hz), 2.29 (1H, ddd, *J* = 18.8, 6.5, 2.0 Hz).

^{13}C{^{1}H} NMR ($CDCl_3$, 101 MHz) δ_C 198.1, 162.9 (d, *J* = 248.1 Hz), 146.5, 131.0 (d, *J* = 8.1 Hz), 130.3 (d, *J* = 3.4 Hz), 129.6, 115.3 (d, *J* = 21.3 Hz), 85.2, 73.8, 53.9, 31.9

^{19}F NMR (376 MHz, $CDCl_3$): δ_F -113.18 (tt, *J* = 8.6, 5.4 Hz).

Enone side-product:

¹H NMR (400 MHz, $CDCl_3$):

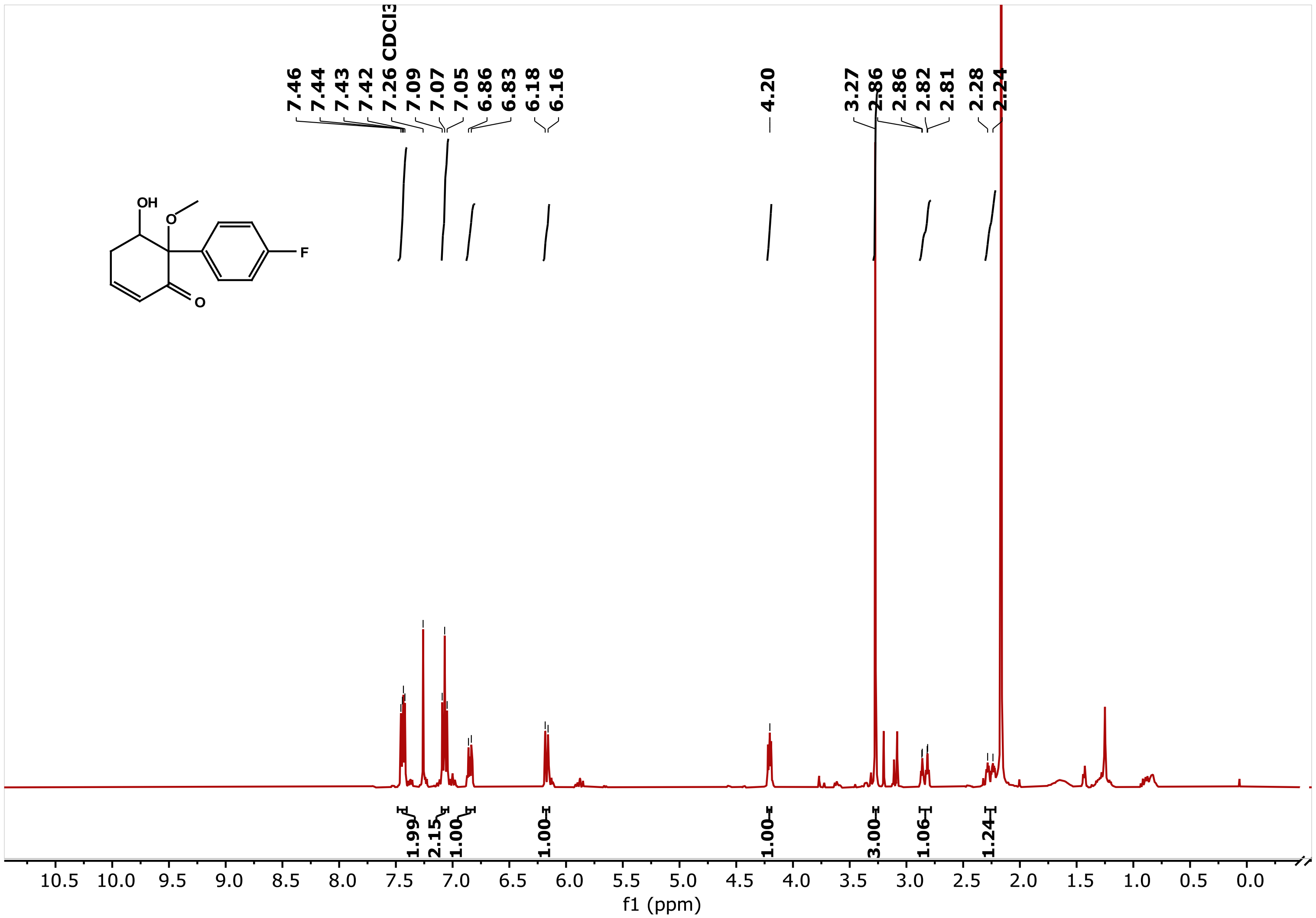

$^{13}C\{^{1}H\}$ NMR ($CDCl_3$, 101 MHz)

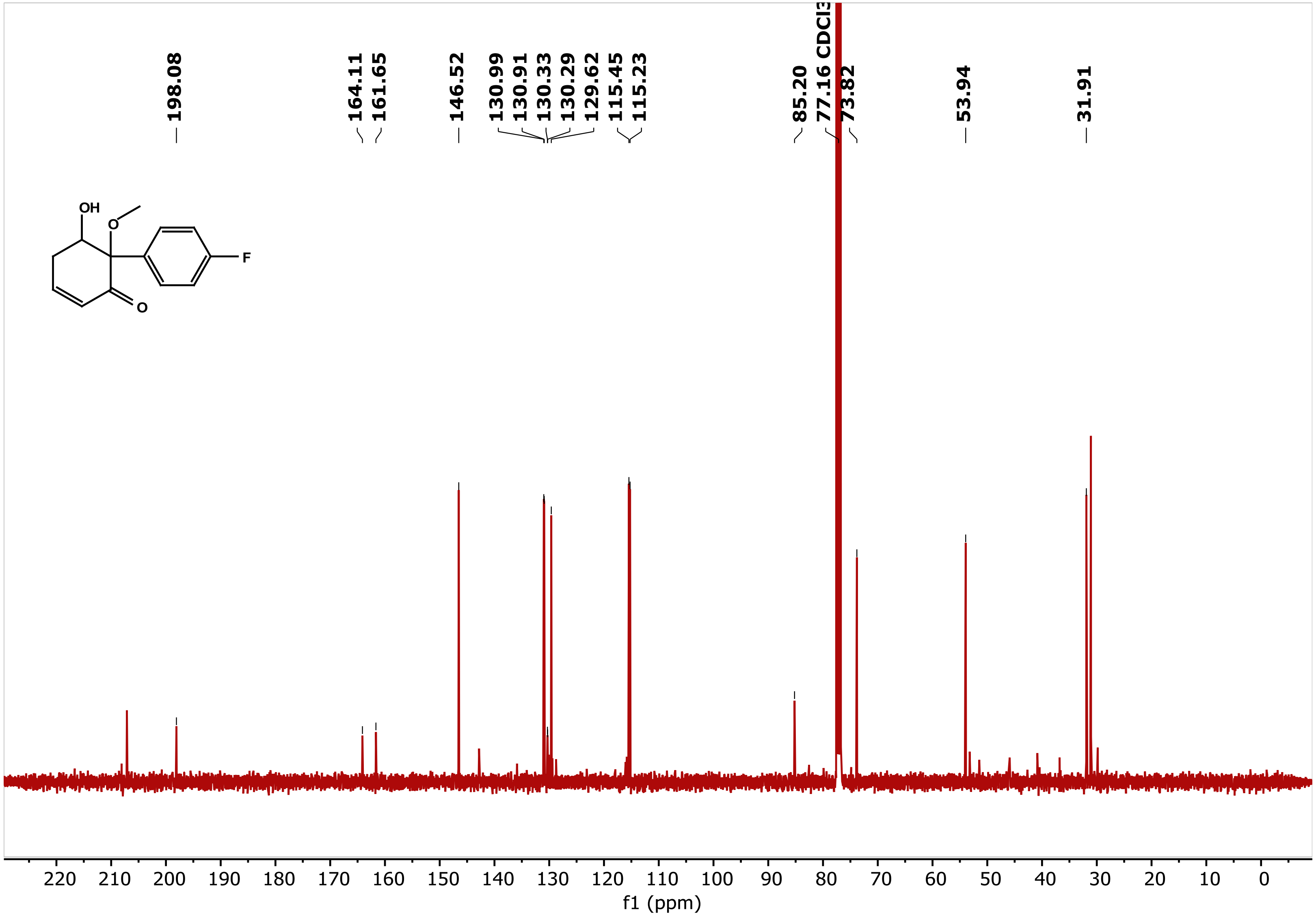

^{19}F NMR (376 MHz, $CDCl_3$):

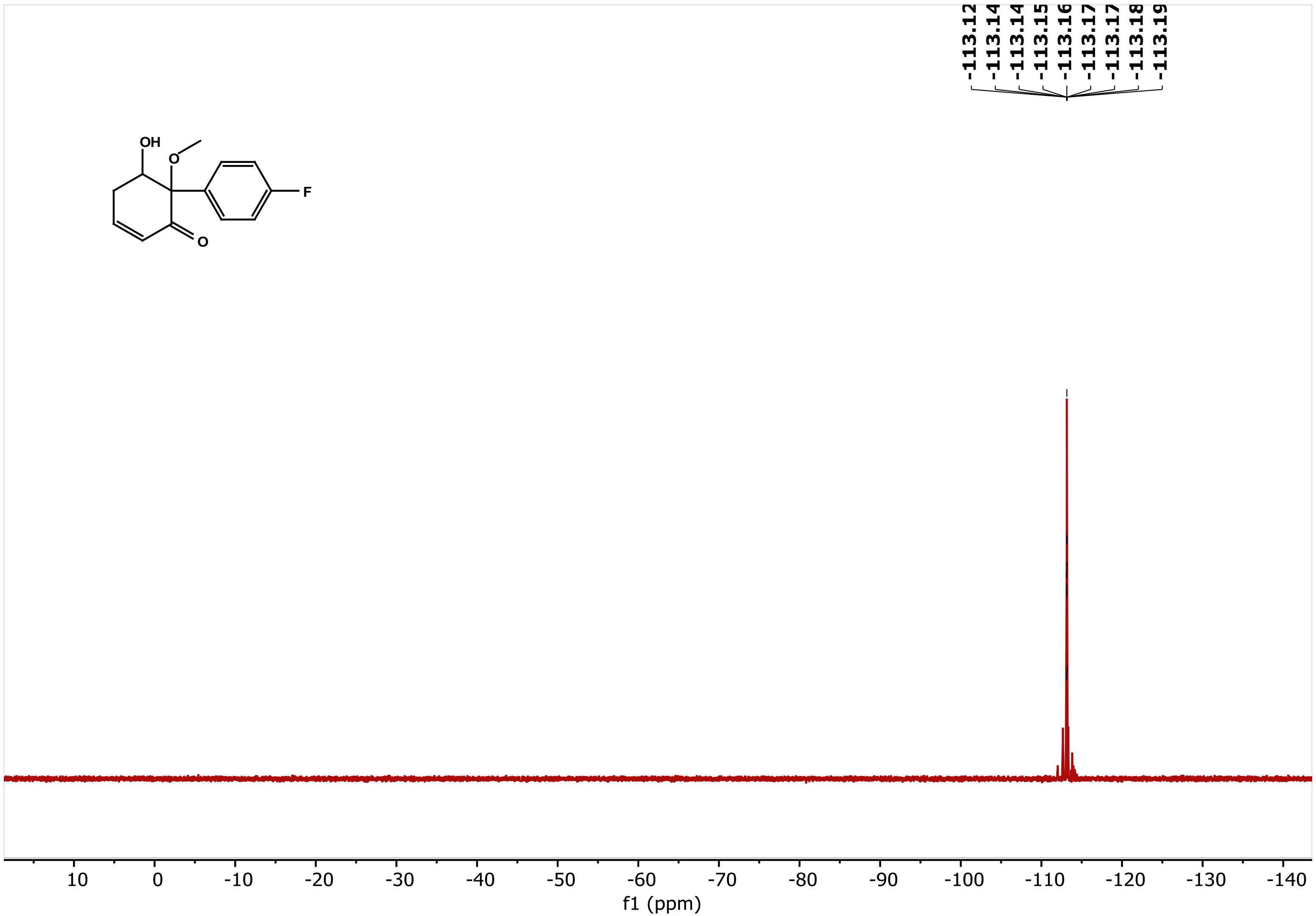

4',6-Difluoro-[1,1'-biphenyl]-2-ol (25)

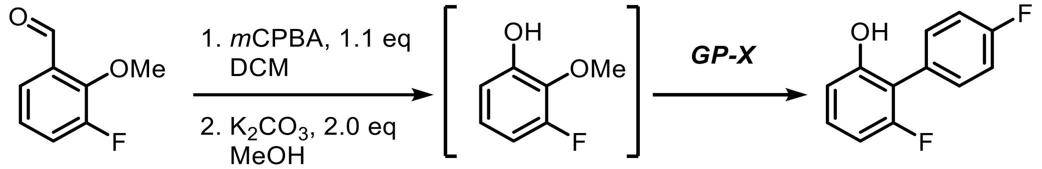

*The synthesis of aryl phenol **25** was performed in a telescoped fashion from 3-fluoro-2-methoxybenzaldehyde, with **GP-3** resuming after the Baeyer-Villiger oxidation / methanolysis to form 3-fluoroguaiacol.*

3-Fluoro-2-methoxybenzaldehyde (539 mg, 3.5 mmol) was dissolved in DCM (9 mL) at rt, and *m*CPBA (959 mg, 3.85 mmol, 70% purity) was added in 5 equal portions at one-minute intervals while stirring. The reaction mixture was left to stir at r.t. for 18 h. The reaction was then quenched with sat. aq. $Na_2S_2O_3$ (3 mL), diluted with DCM (20 mL) and washed sequentially with sat. aq. Na_2SO_3 (1 × 20 mL), brine (1 × 20 mL) and $NaHSO_3$ (3 × 50 mL). The organic portion was then dried with Na_2SO_4 and concentrated *in vacuo*.

The resultant residue was diluted with MeOH (9 mL), to which K_2CO_3 (966 mg, 7.00 mmol) was added on stirring at r.t. After 30 min, the reaction mixture was concentrated *in vacuo*, and suspended in Et_2O (15 mL). The mixture was washed with H_2O (2 × 10 mL), dried with Na_2SO_4 and concentrated *in vacuo* to afford the crude phenol; this was purified via automated silica gel column chromatography (0 to 5% Et_2O in pentane) to yield 3-fluoroguaiacol as a colourless oil (160 mg, 1.13 mmol, 38%). This compound is volatile; as such its yield was calculated by dissolving the product in PhMe and adding a known amount of internal standard.

Aryl phenol **25** was synthesised according to **GP-3** on a 0.13 mmol scale using 3-fluoroguaiacol and 10-(4-fluorophenyl)-10H-dibenzo[b,e][1,4]thiabismine 5,5-dioxide **2a**. The crude compound was purified by automated silica gel column chromatography (0 to 10 % Et_2O in pentane) to yield the title compound as an off white solid (135 mg, 0.66 mmol, 56%). Characterisation data were consistent with literature values: ^1H, $^{13}C\{^1H\}$, ^{19}F, HRMS and MP.[2]

^{1}H NMR (400 MHz, $CDCl_3$): δ_H 7.40 (2H, dd, *J* = 8.9, 5.3, Hz), 7.27 – 7.16 (3H, m), 6.80 (1H, app. dt, *J* = 8.3, 1.1 Hz), 6.75 (1H, ddd, *J* = 9.3, 8.3, 1.1 Hz), 5.03 (1H, s).

$^{13}C\{^1H\}$ NMR ($CDCl_3$, 101 MHz) δ_C 162.8 (d, *J* = 248.5 Hz), 160.2 (d, *J* = 245.6 Hz), 153.9 (d, *J* = 5.6 Hz), 132.3 (d, *J* = 7.8 Hz), 129.4 (d, *J* = 10.5 Hz), 125.9 (d, *J* = 3.4 Hz), 116.4 (d, *J* = 21.6 Hz), 115.7 (d, *J* = 18.9 Hz), 111.4 (d, *J* = 3.1 Hz), 107.8 (d, *J* = 22.8 Hz).

^{19}F NMR (377 MHz, $CDCl_3$): δ_F -112.69 (1F, tt, *J* = 8.5, 5.3 Hz), -115.32 (1F, dd, *J* = 9.3, 6.7 Hz).

IR (ATR-IR, neat) υ_{max}/cm^{-1}: 3547, 3019, 2926, 1622, 1601, 1517, 1464, 1404, 1330, 1294, 1214, 1176, 1159, 1094, 1001.

HRMS (ESI$^-$): *m/z* calcd. for $C_{12}H_7F_2O$ $[M-H]^-$: 205.0465. Found: 205.0474.

MP: 73-76 °C.

5.3 Procedure for Dealkoxylative Arylation on a 1.0 mmol Scale

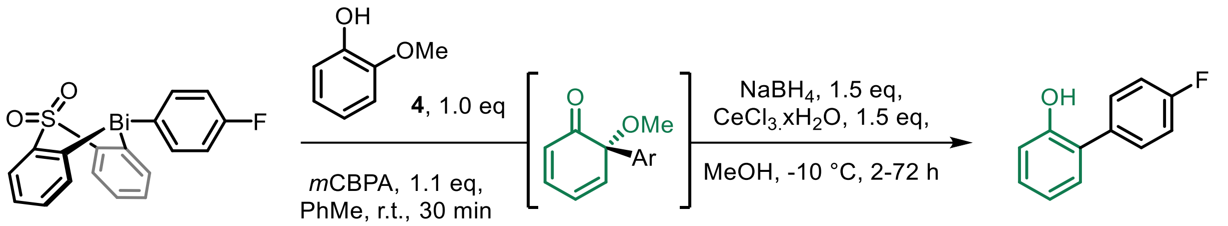

2a **5**

In an amberised round-bottomed flask, aryl bismacycle **2a** (520 mmol, 1.0 mmol) and guaiacol **4** (124 mg, 1.0 mmol) were suspended in PhMe (50 mL) in the presence of internal standard 4,4'-bis(trifluoromethyl)-1,1'-biphenyl (8.71 mg, 0.03 mmol) at rt. *m*CPBA (270 mg, 1.1 mmol, 70% purity) was added in 10 equal portions at 30 second intervals while stirring, and the reaction mixture was left for 30 min. The resultant orange solution was quenched with sat. aq. Na2S2O5 (0.01 mL), left to stir for 5 min, then passed through a plug of basic alumina (*ca* 30 mL). The intermediate cyclohexadienone was eluted with EtOAc (100 mL) and concentrated into a flask containing cerium chloride hydrate (560 mg, 1.50 mmol). This was diluted with MeOH (10 mL) and cooled to -10 °C, after which time sodium borohydride (56.8 mg, 1.05 mmol) was added in 10 equal portions at 40 second intervals while stirring. The reaction was left to warm to rt until no intermediate cyclohexadienone could be observed by ^{1}H NMR spectroscopy. The reaction mixture was then quenched with HCl (4 M in dioxane; 8 mmol, 2 mL), left to stir for 5 min, and concentrated *in vacuo* to afford the crude arylated phenol, which was purified by automated silica gel column chromatography (5-10% Et_2O in cyclohexane) to yield 4'-fluoro-[1,1'-biphenyl]-2-ol **5** as a yellow oil (90.0 mg, 0.48 mmol, 48%).

Characterisation data for 2-arylphenol **5** are listed in Section 5.1.

6. Telescoped Transmetallation, Arylation and Bismacycle Recovery

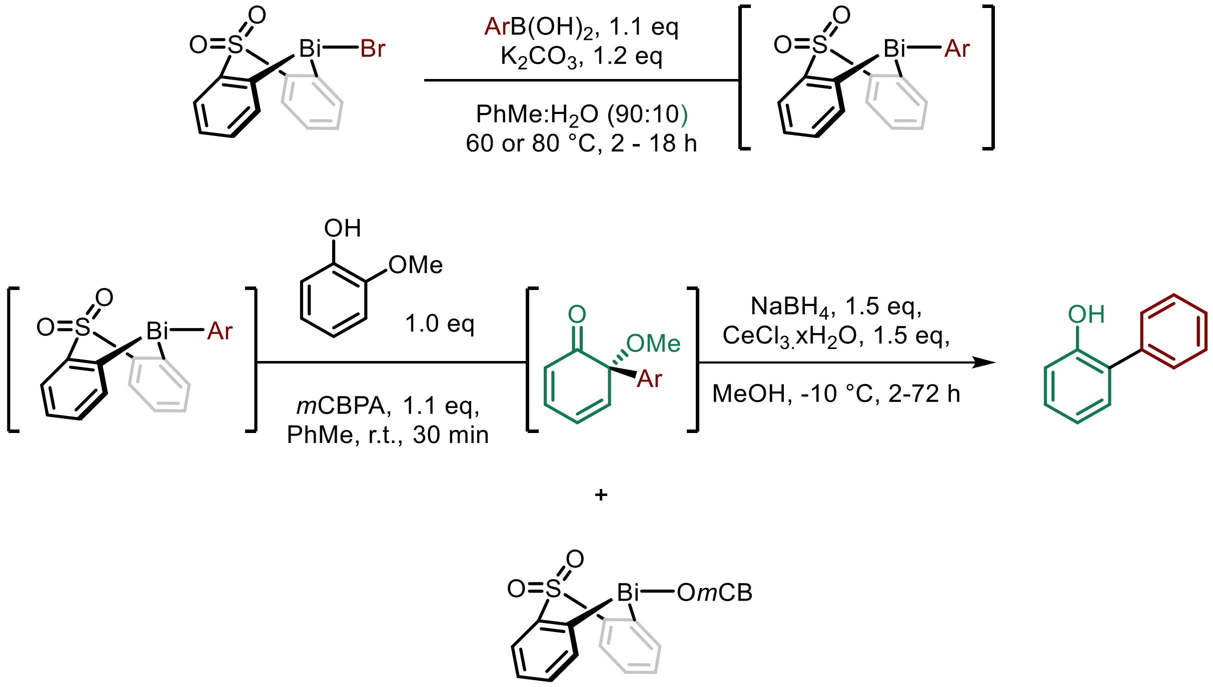

10-Bromo-10H-dibenzo[b,e][1,4]thiabismine 5,5-dioxide **1-Br** (759 mmol, 1.5 mmol), K_2CO_3 (182 mg, 1.32 mmol), (4-fluorophenyl)boronic acid (231 mg, 1.65 mmol) and 4,4'-bis(trifluoromethyl)-1,1'-biphenyl (internal standard; 13.6 mg, 0.04 mmol) were suspended in a mixture of toluene (15 mL) and water (1.5 mL) and stirred at 60 °C (aluminium heating block) until no starting material was observed by ^{1}H NMR spectroscopy. Once cooled, aqueous NaOH (2.0 M; 50 mL) was added to the reaction mixture, which was then diluted with 50 mL EtOAc. The aqueous phase was separated and the organic portion was washed with water (3 × 50 mL) and brine (3 × 50 mL). The combined organic portions were dried over $MgSO_4$, filtered and concentrated *in vacuo*. To the residue was added guaiacol (185 mg, 1.5 mmol) and toluene (75 mL). *m*CPBA (410 mg, 1.65 mmol, 70% purity) was added in 10 equal portions at 30-second intervals while stirring. The reaction mixture was left for 30 min.

The resultant orange solution was then quenched with sat. aq. $Na_2S_2O_5$ (0.01 mL), left to stir for 5 min, then concentrated *in vacuo*. The residue was suspended in TBME (10 mL) and stirred for 5 min, then pentane (90 mL) was added. The suspension was left stirring at 0 °C for 5 min, and the resultant solids were filtered under reduced pressure and collected as 5,5-dioxido-10H-dibenzo[b,e][1,4] thiabismin-10-yl 3-chlorobenzoate (716 mg, 1.23 mmol, 82%) as a colourless solid. The filtrate was then passed through a plug of basified alumina (25 mL) and the

intermediate cyclohexadienone was eluted with EtOAc (70 mL) and concentrated into a flask containing cerium chloride hydrate (392 mg, 1.05 mmol) and 1-bromo-4-fluoronaphthalene (internal standard; 59.67 mg, 0.26 mmol). This was diluted with MeOH (7.0 mL) and cooled to -10 °C, after which time sodium borohydride (40 mg, 1.05 mmol) was added in 10 equal portions at 40 second intervals while stirring. The reaction was left to warm to rt until no intermediate cyclohexadienone could be observed by TLC. The reaction mixture was then quenched with HCl (4 M, 7.0 mL), left to stir for 5 min, and the organics extracted with Et_2O (3 × 10 mL). The combined organics were washed once with brine (10 mL), dried with anhydrous sodium sulfate, and concentrated *in vacuo* to afford the crude arylated phenol, which was purified by automated silica gel column chromatography (5-10 % Et_2O in pentane) to yield 4'-fluoro-[1,1'-biphenyl]-2-ol (60 mg, 0.32 mmol, 22%).

5,5-Dioxido-10H-dibenzo[b,e][1,4] thiabismin-10-yl 3-chlorobenzoate (1-OmCB)

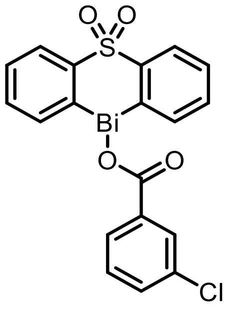

Characterisation data were consistent with literature values: ^{1}H, ^{13}C{^{1}H}, ^{19}F, HRMS and MP.[2]

^{1}H NMR (400 MHz, $CDCl_3$): δ_H 8.79 (2H, d, *J* = 7.4 Hz), 8.39 (2H, d, *J* = 7.7 Hz), 8.07 (1H, app. t, *J* = 1.1 Hz), 7.99 (1H, app. dt, J 7.7, 1.1 Hz), 7.72 (2H, app. td, *J* = 7.4, 1.0), 7.54 - 7.50 (1H, m), 7.47 (2H, app. td, *J* = 7.7, 1.0 Hz), 7.39 (1H, app. t, *J* = 7.9 Hz).

^{13}C{^{1}H} NMR (101 MHz, $CDCl_3$): δ_C 185.0, 172.7, 141.1, 136.1, 135.5, 134.5, 134.2, 132.7, 130.5, 129.7, 129.0, 128.8, 128.5.

IR (ATR-IR, neat) υ_{max}/cm^{-1}: 2978, 1709, 1638, 1607, 1569, 1490, 1433, 1406, 1366 ,1339, 1307, 1270, 1253, 1221, 1202, 1161, 1141, 1110, 1086, 1068, 1035, 1012, 979.

HRMS (ESI$^+$): *m/z* calcd. for $C_{12}H_8BiO_2S$ [M-$C_7H_4ClO_2$]$^+$: 425.0049. Found: 425.0040.

MP: 197-200 °C.

7. Synthesis and Characterisation of Authentic Compounds

4,4''-Difluoro-[1,1':3',1''-terphenyl]-2'-ol

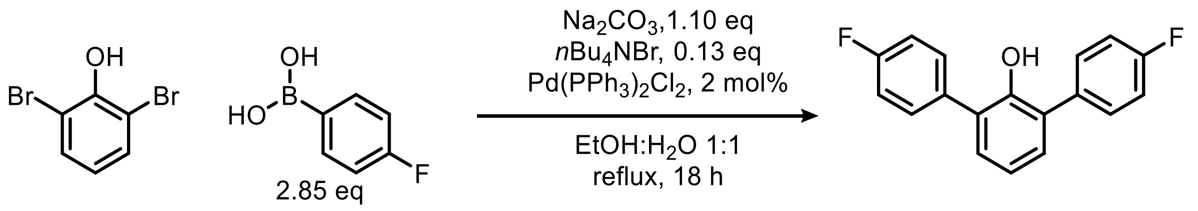

Prepared according to literature procedure:[13] 2,6-Dibromophenol (202 mg, 0.80 mmol), (4-fluorophenyl)boronic acid (318 mg, 2.28 mmol), Na_2CO_3 (93.0 mg, 0.88 mmol), tetrabutylammonium bromide (33.0 mg, 0.10 mmol) and bis(triphenylphosphine)palladium(II) dichloride (12.0 mg, 17.0 µmol) were suspended in a 1:1 mixture of EtOH:H_2O (86 mL) and heated to reflux (aluminium heating block) for 18 h. The reaction mixture was cooled to r.t. and the EtOH was removed *in vacuo*. The reaction was extracted with DCM (3 × 15 mL), and the combined organic potions were dried over sodium sulfate and concentrated *in vacuo*. The crude material was purified *via* automated silica gel column chromatography (1% Et_2O in pentane) to yield the title compound as off-white crystals (14.0 mg, 0.05 mmol, 6%). Characterisation data were consistent with literature values: ^{1}H, ^{13}C{^{1}H},^{19}F NMR.[9]

^{1}H NMR (400 MHz, $CDCl_3$): δ_H 7.54 (4H, dd, *J* = 8.7, 5.4 Hz), 7.27 (2H, d, *J* = 7.6 Hz), 7.19 (4H, app. t, *J* = 8.7 Hz), 7.08 (1H, t, *J* = 7.6 Hz), 5.26 (1H, s).

^{13}C{^{1}H} NMR (101 MHz, $CDCl_3$): δ_C 162.5 (d, *J* = 247.2 Hz), 149.4, 133.5 (d, *J* = 3.3 Hz), 131.2 (d, *J* = 8.1 Hz), 130.2, 128.0, 121.0, 115.9 (d, *J* = 21.6 Hz).

^{19}F NMR (376 MHz, $CDCl_3$): δ_F -114.26 (tt, *J* = 8.7, 5.4 Hz).

IR (ATR-IR, neat) υ_{max}/cm^{-1}: 3542, 2923, 2855, 2252, 1892, 1666, 1506, 1468, 1445, 1327, 1271, 1228, 1165, 1091, 1018, 666.

HRMS (ESI$^-$): *m/z* calcd. for $C_{18}H_{11}F_2O$ [M-H]$^-$: 281.0778. Found: 281.0784.

MP: 76-78 °C.

8. NMR Spectra

(4-Fluorophenyl)bismuth

^{1}H NMR (400 MHz, $CDCl_3$):

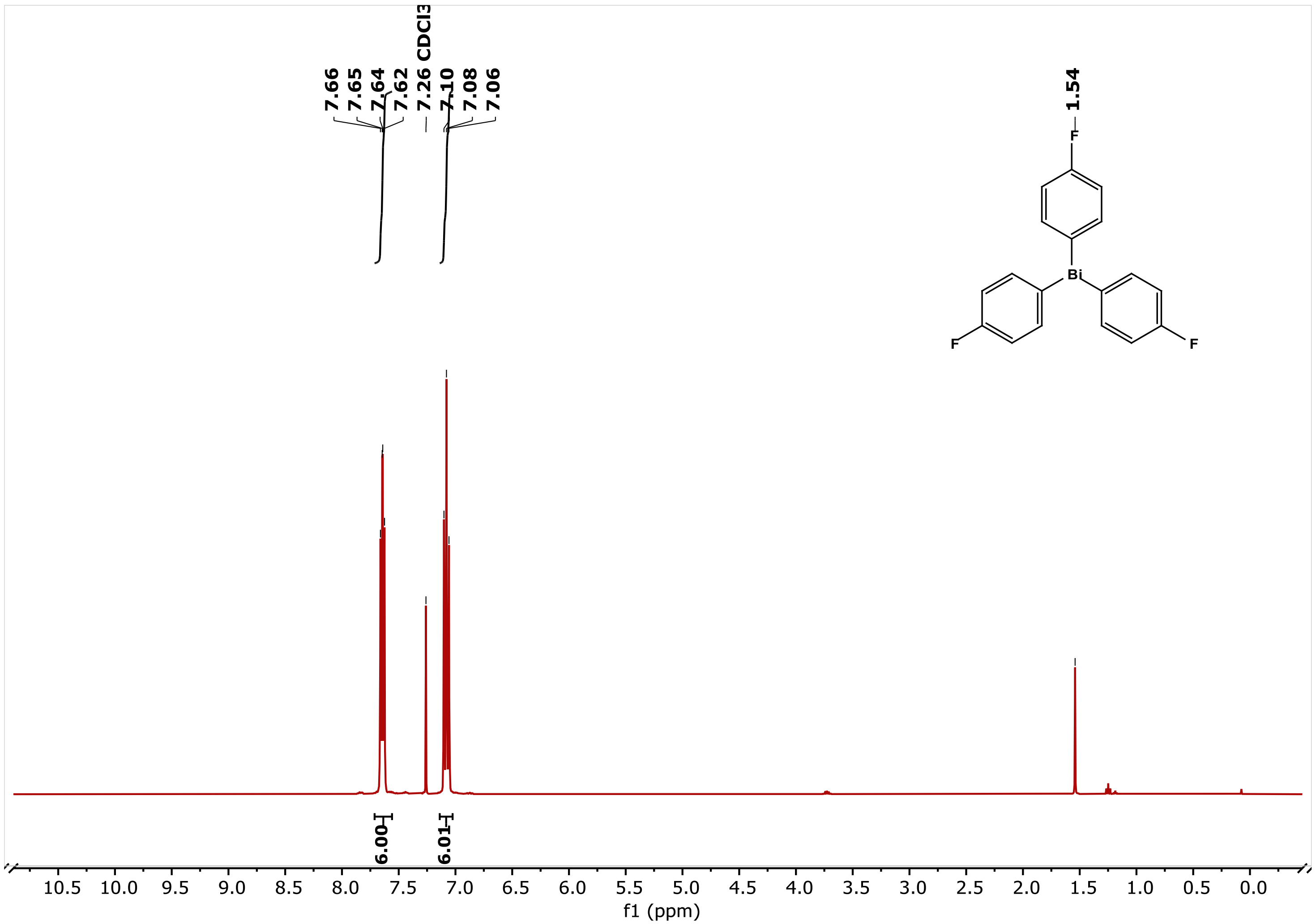

$^{13}C\{^1H\}$ NMR (101 MHz, $CDCl_3$):

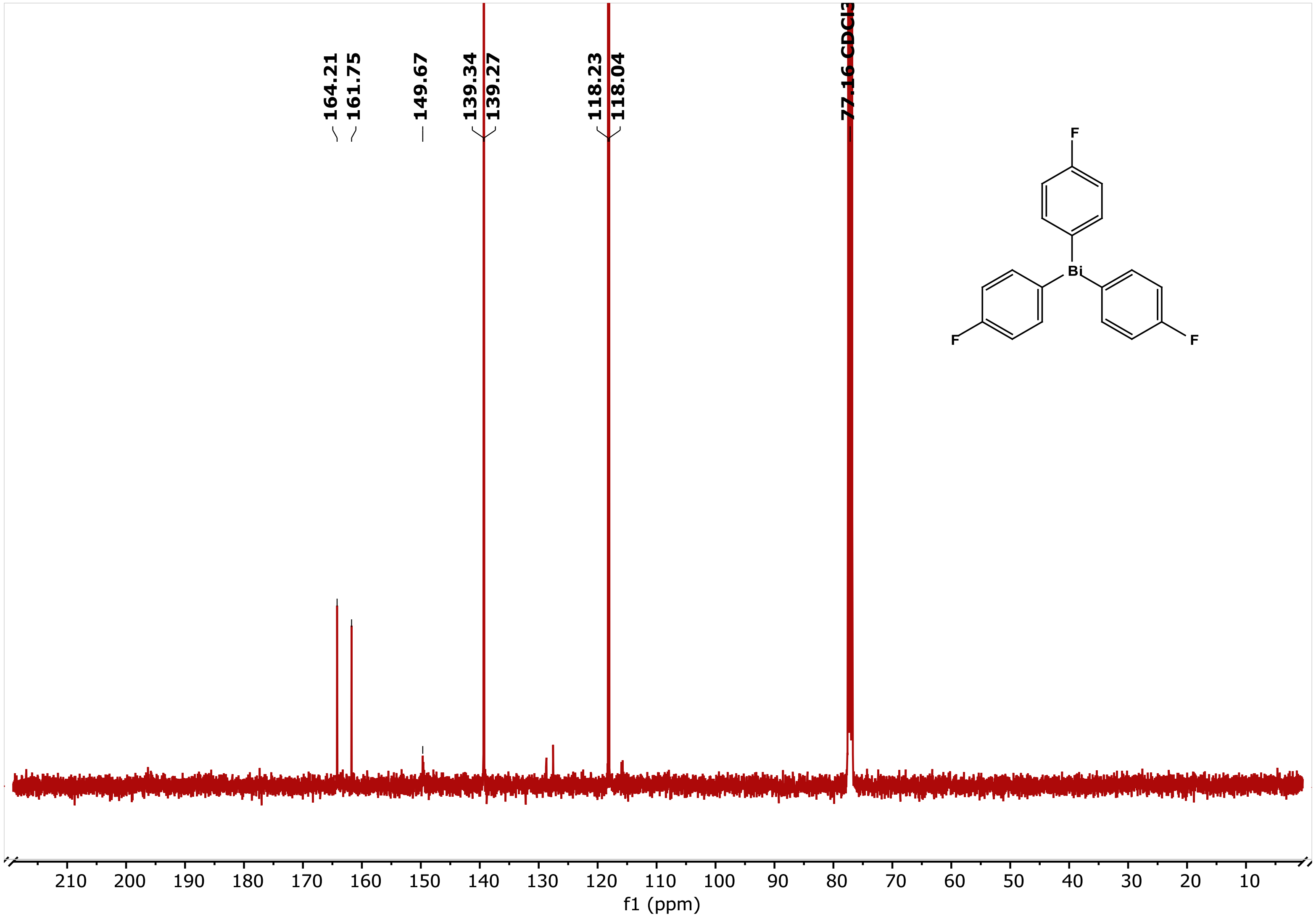

¹⁹F NMR (376 MHz, $CDCl_3$):

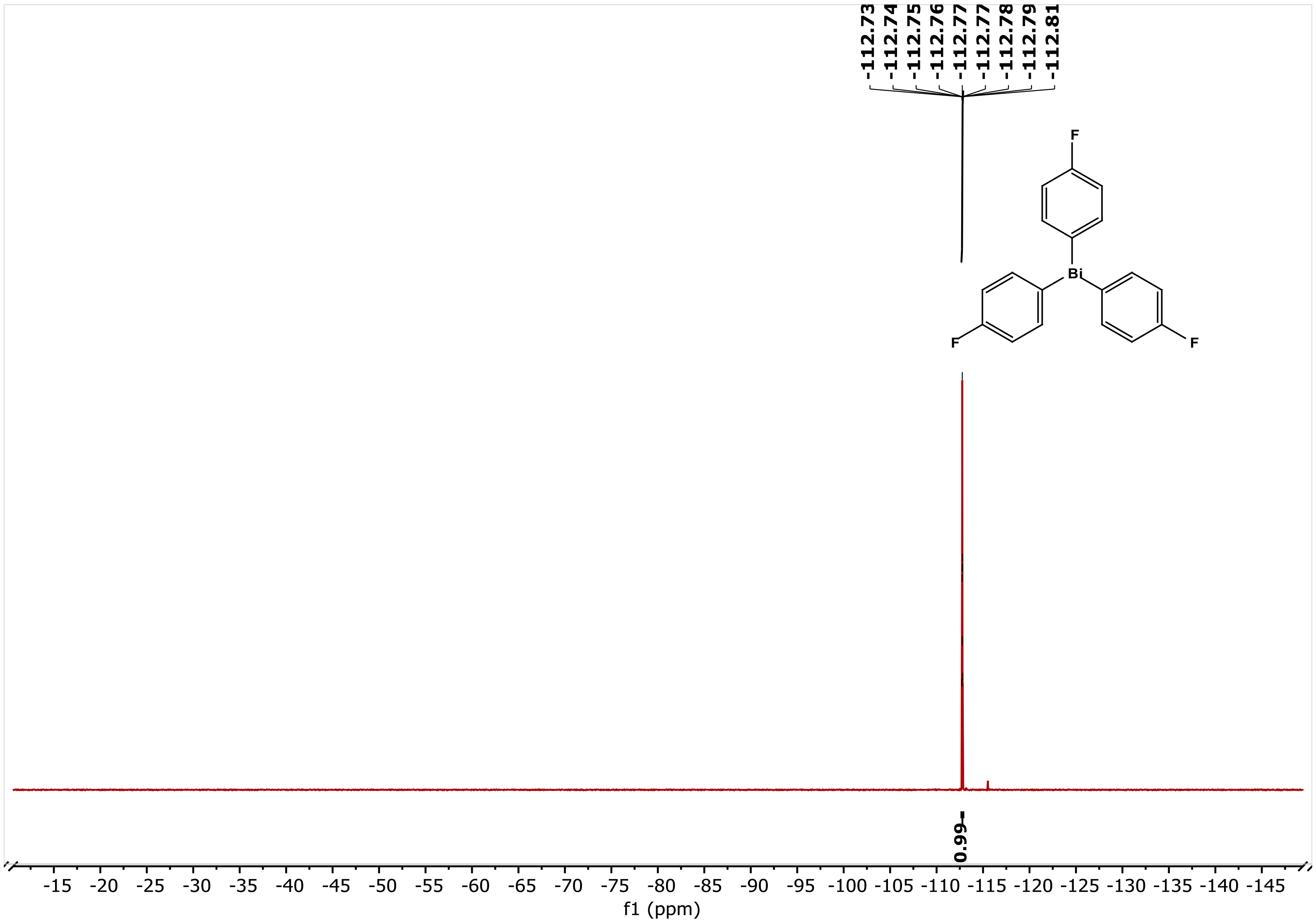

5,5-Dioxido-10H-dibenzo[b,e][1,4]thiabismin-10-yl 4-methylbenzenesulfonate (1-OTs)
^{1}H NMR (400 MHz, DMSO-d_6):

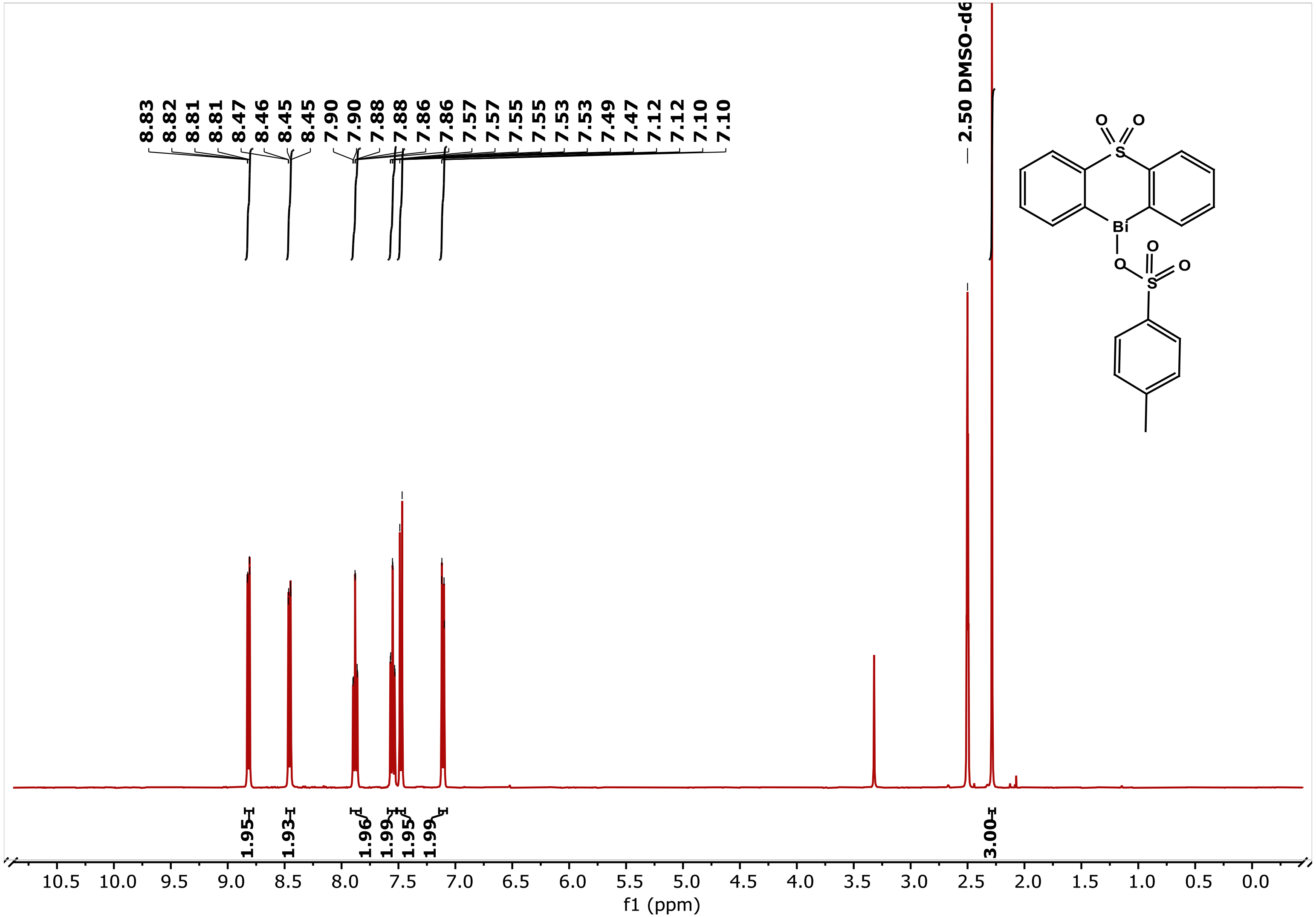

$^{13}C\{^1H\}$ **NMR** (101 MHz, DMSO-d_6):

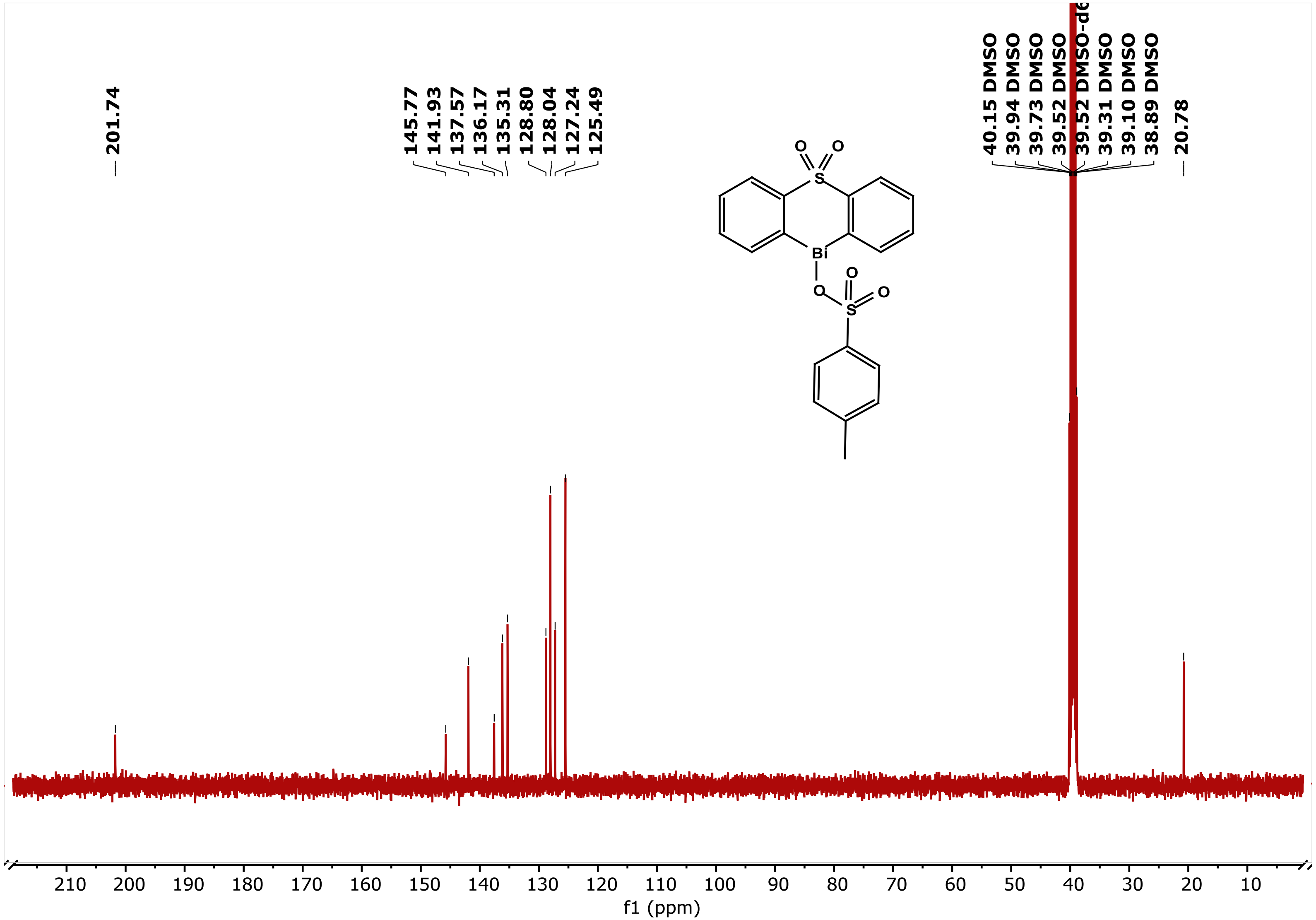

2-Hydroxyphenyl acetate

^{1}H NMR (400 MHz, $CDCl_3$):

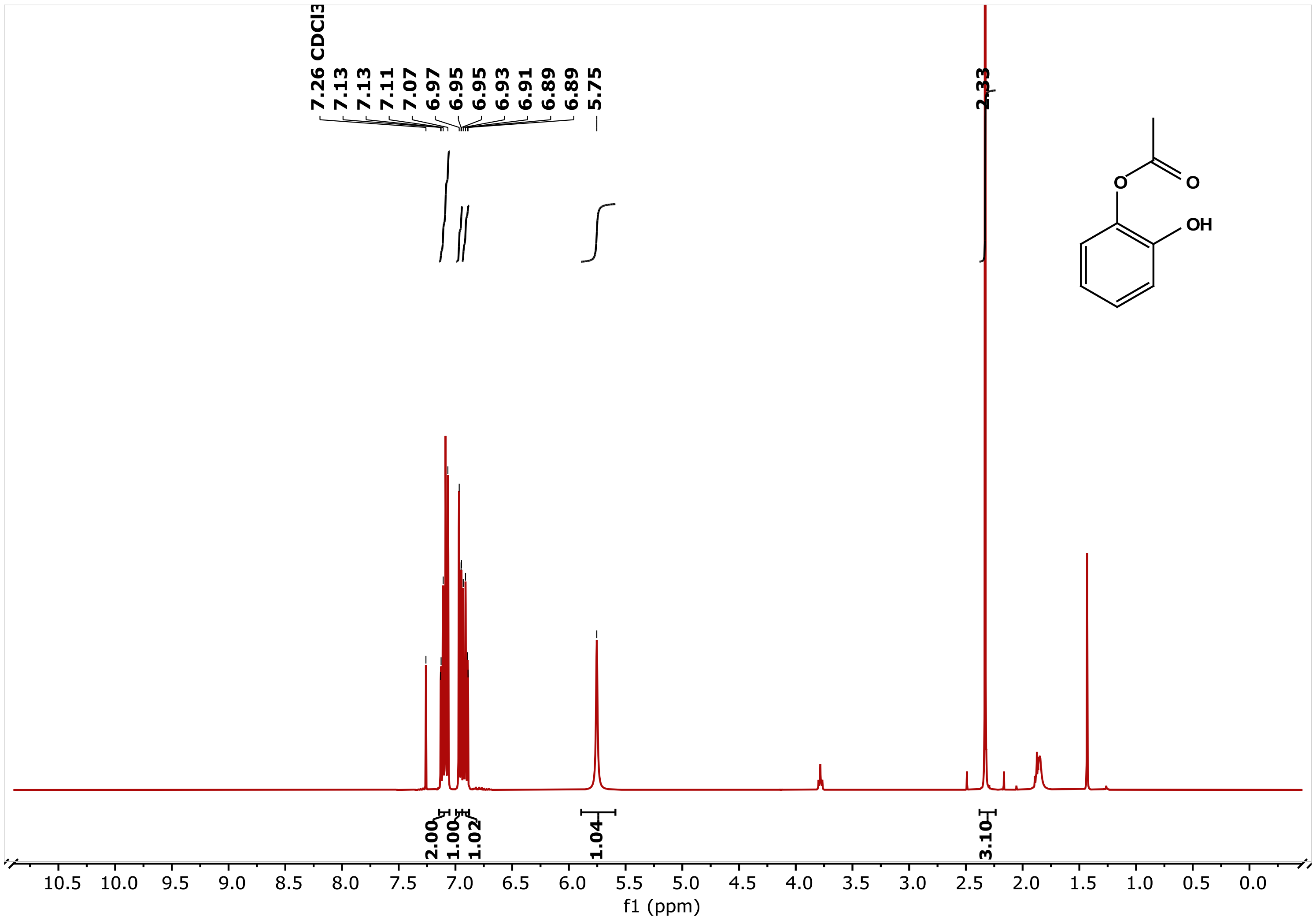

^{13}C{^{1}H} NMR (101 MHz, $CDCl_3$):

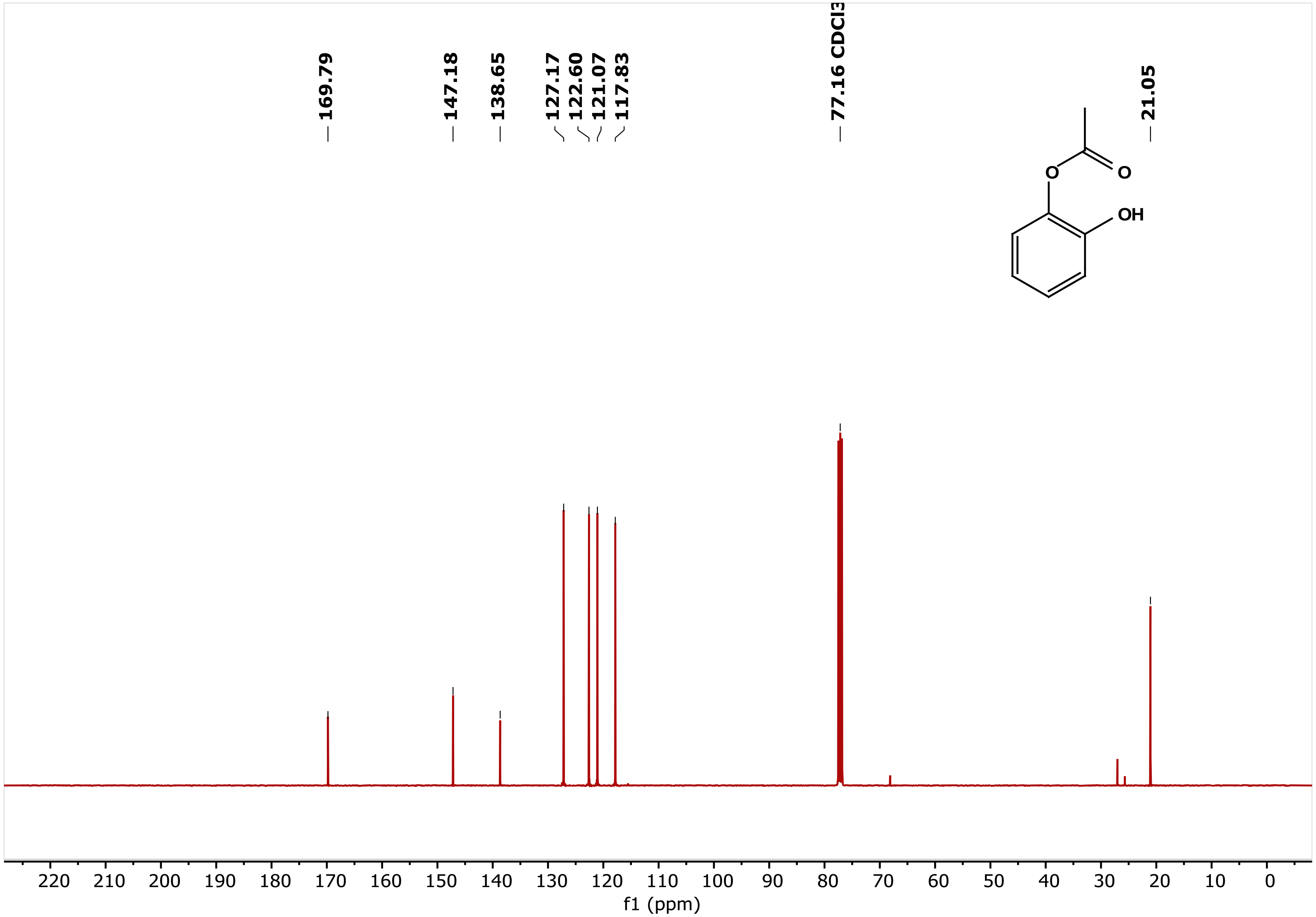

Methyl (*E*)-3-(4-hydroxy-3-methoxyphenyl)acrylate

^{1}H NMR (400 MHz, $CDCl_3$):

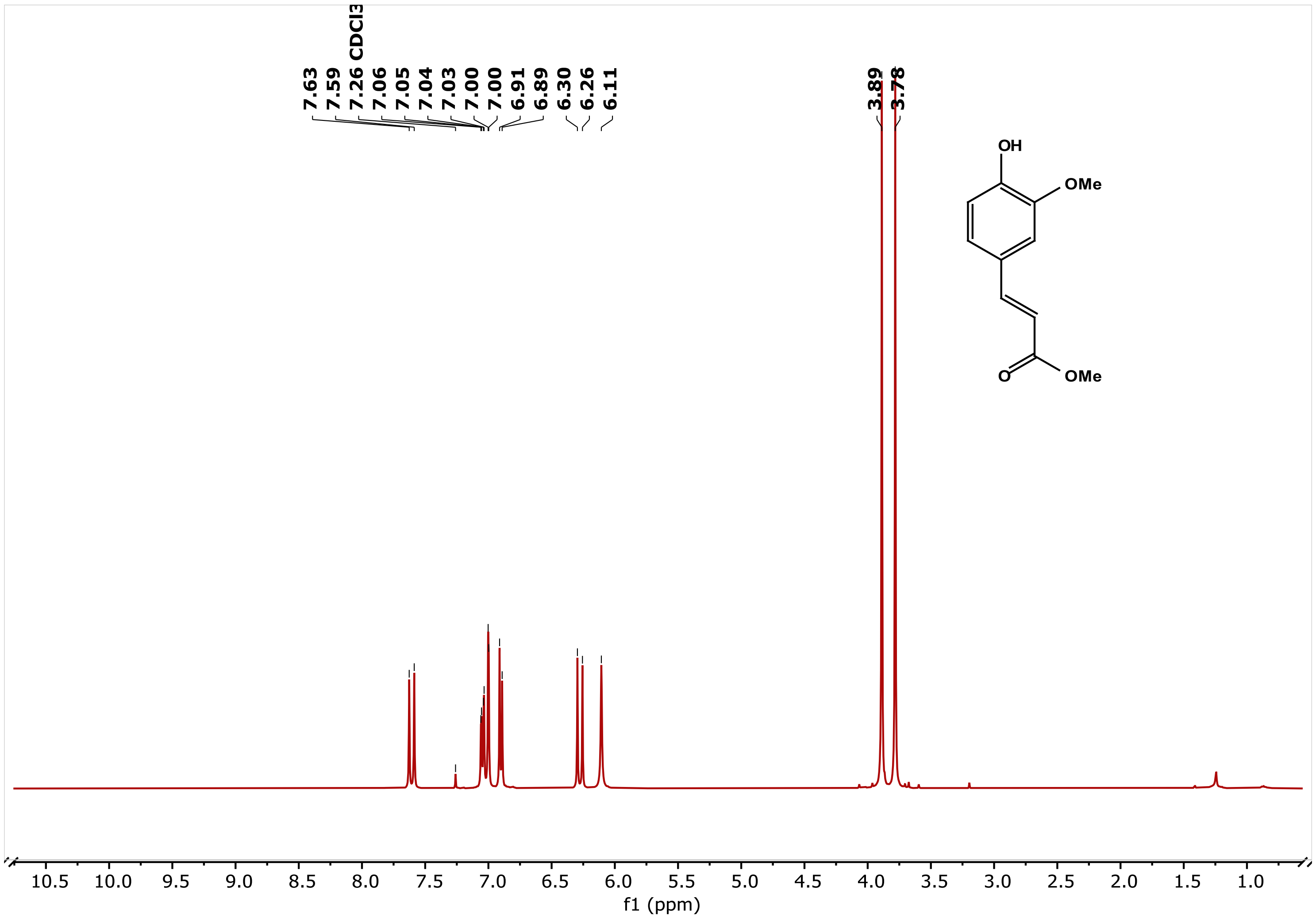

$^{13}C\{^1H\}$ **NMR** (101 MHz, $CDCl_3$):

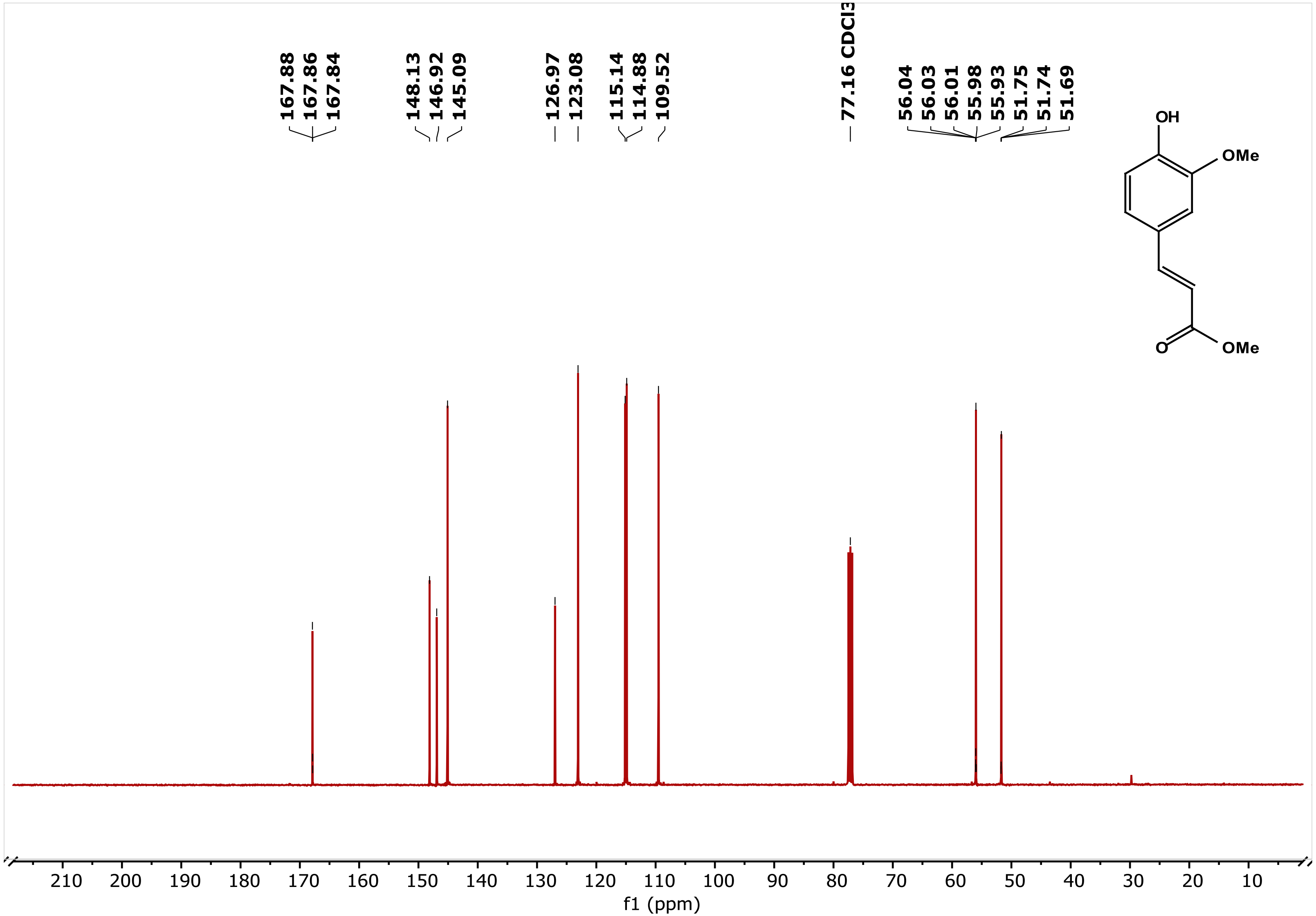

2,3-Dimethoxyphenol

^{1}H NMR (400 MHz, $CDCl_3$):

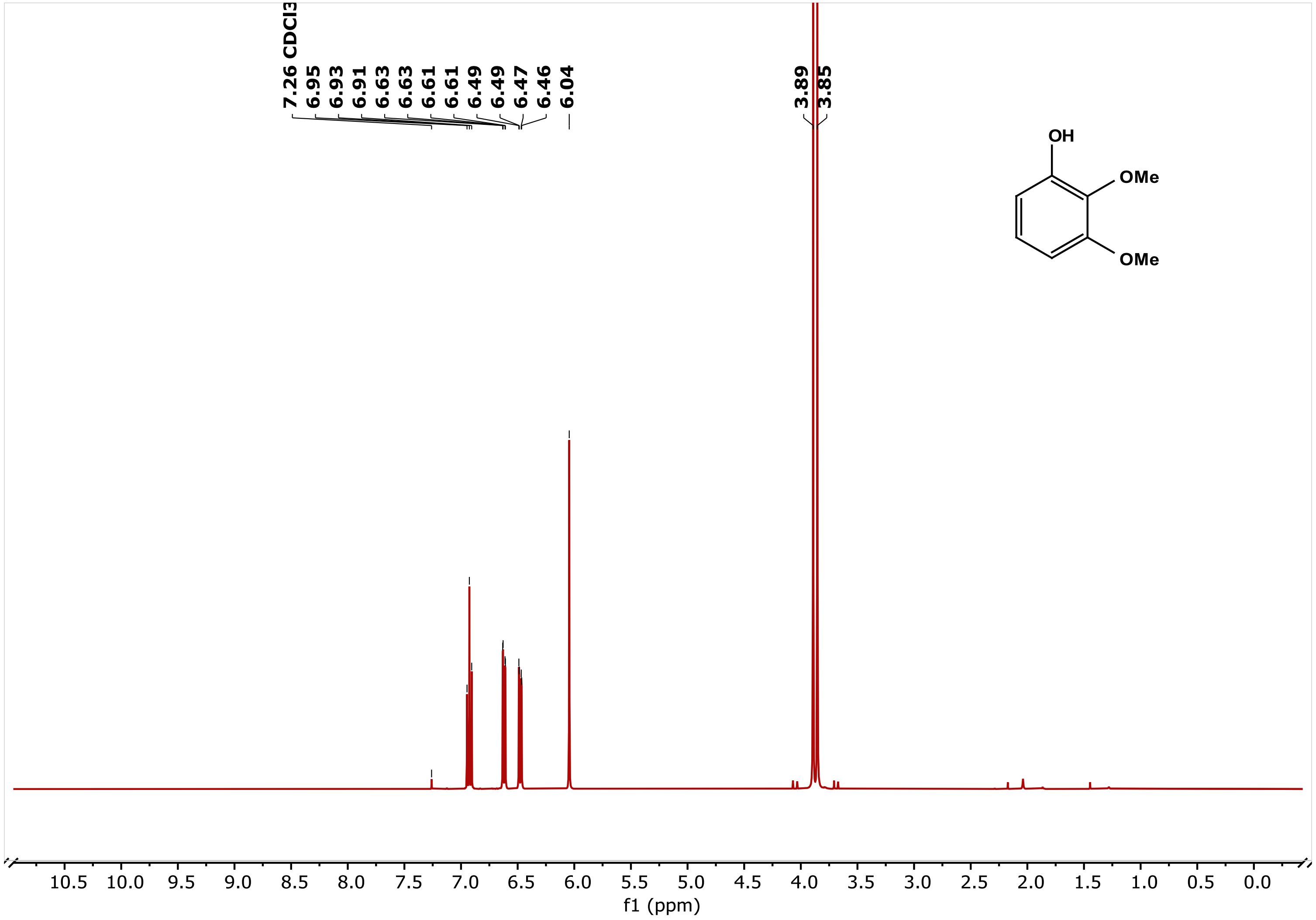

^{13}C{^{1}H} NMR (101 MHz, $CDCl_3$):

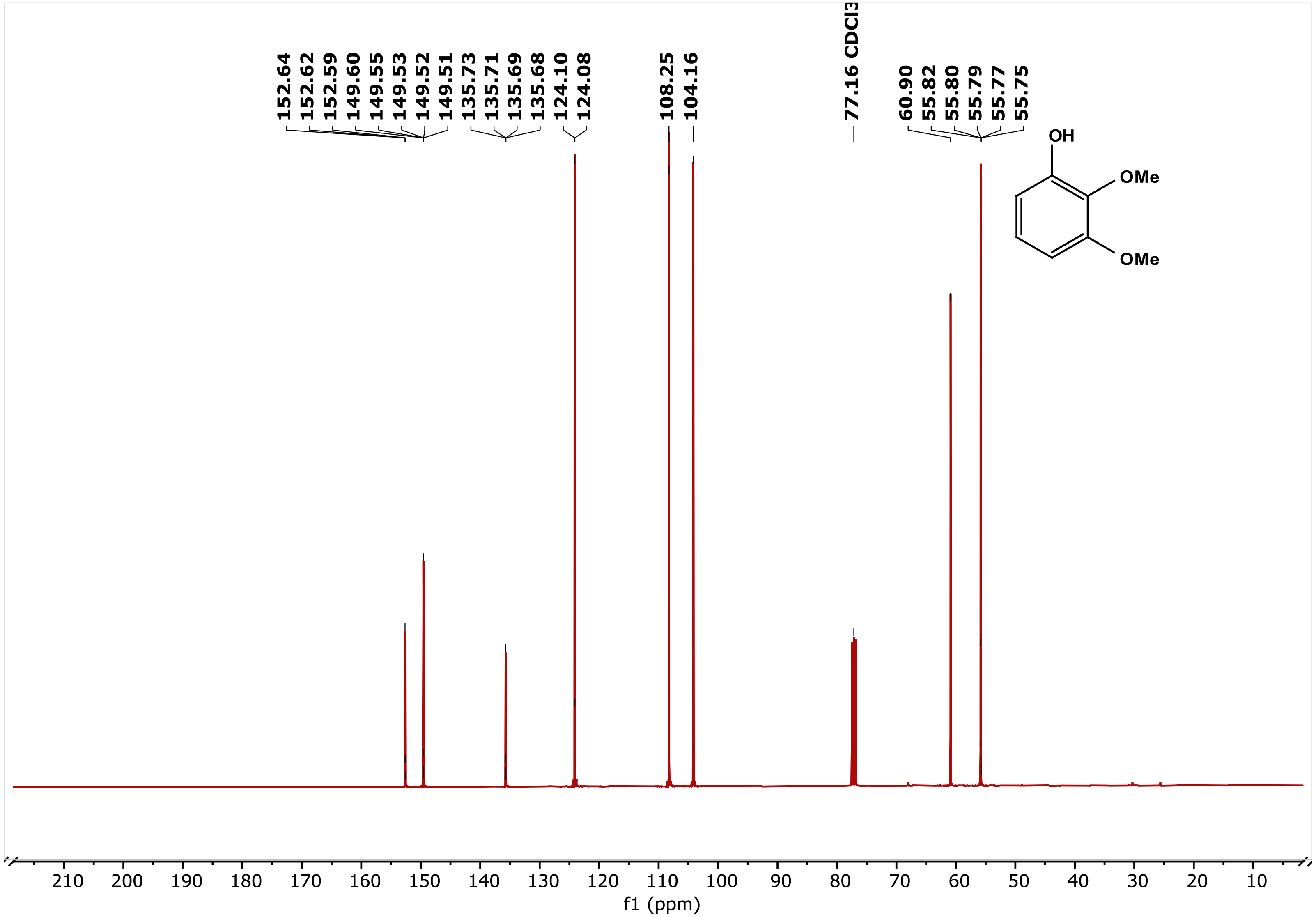

4'-Fluoro-[1,1'-biphenyl]-2-ol (5)

^{1}H NMR (400 MHz, $CDCl_3$):

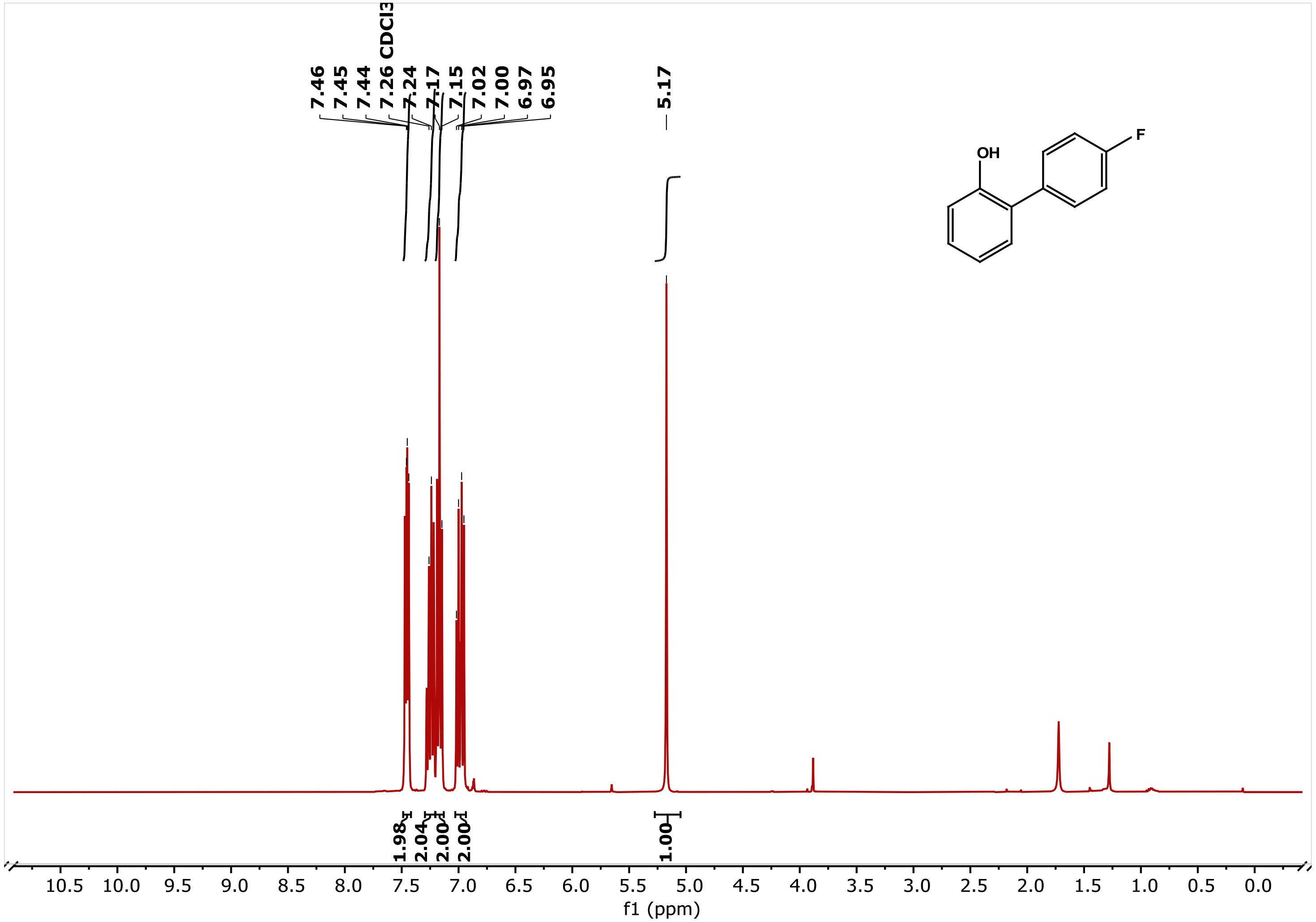

^{13}C{^{1}H} NMR (101 MHz, $CDCl_3$):

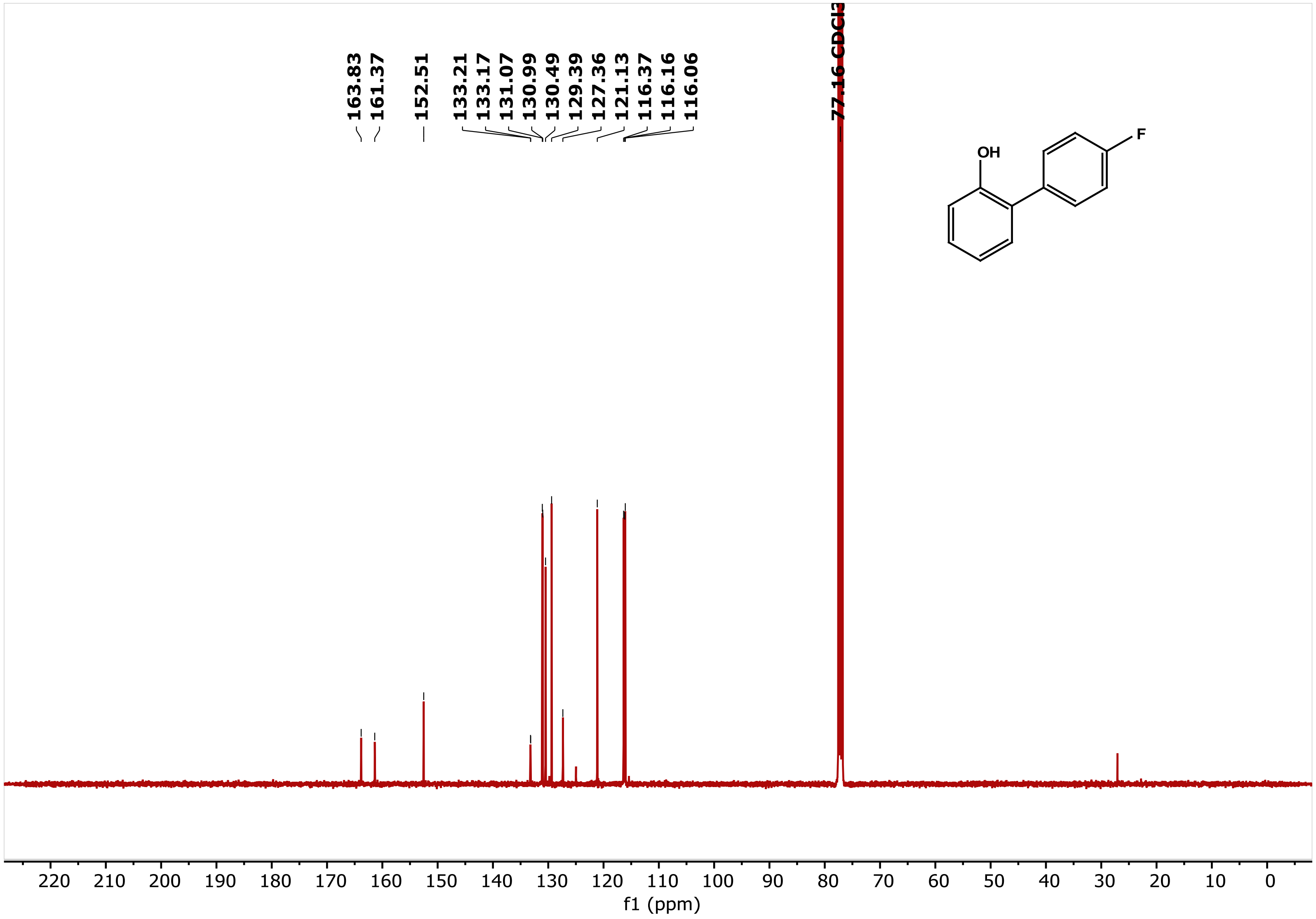

^{19}F NMR (377 MHz, $CDCl_3$):

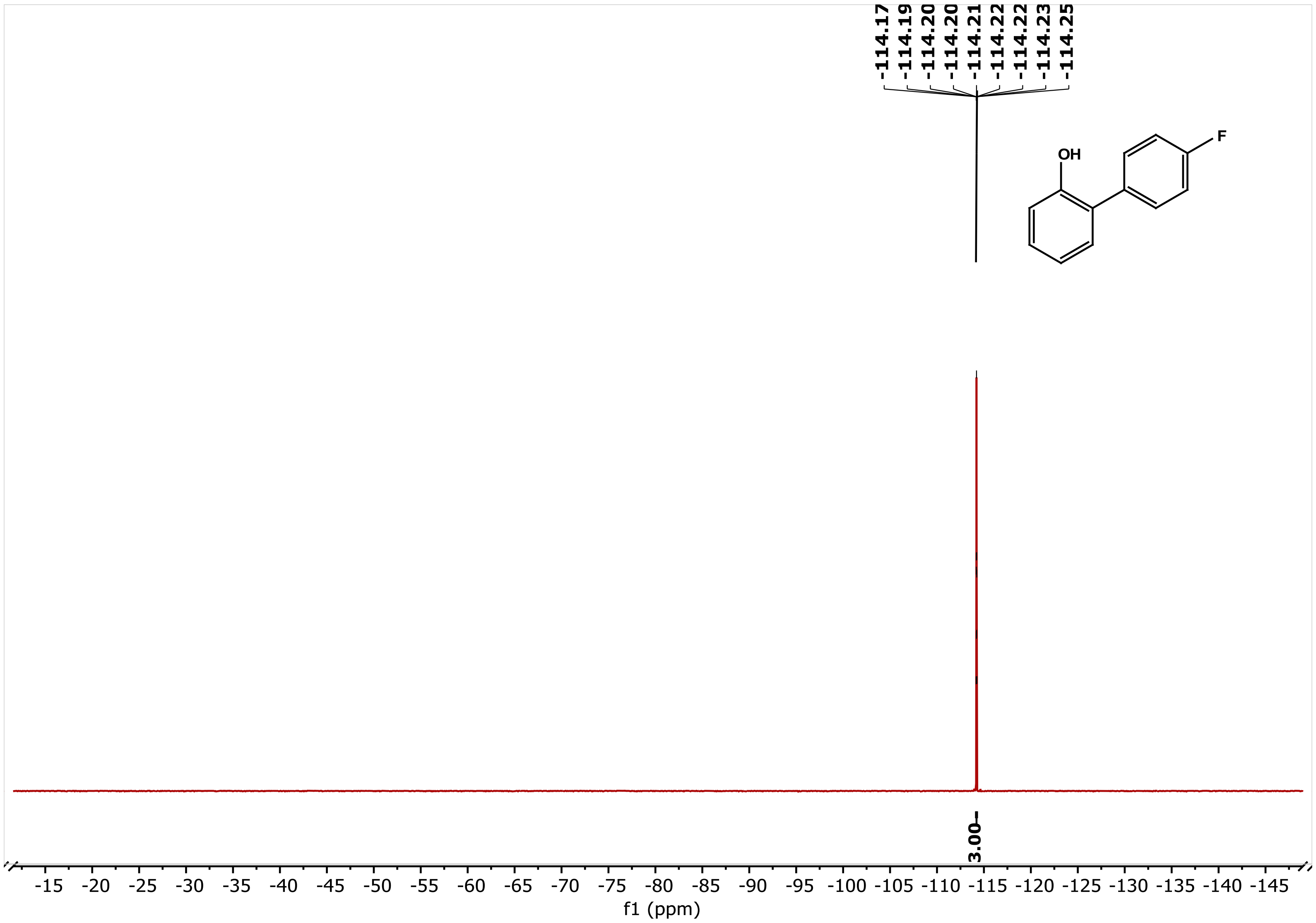

4'-Methoxy-[1,1'-biphenyl]-2-ol (6)

^{1}H NMR (400 MHz, $CDCl_3$):

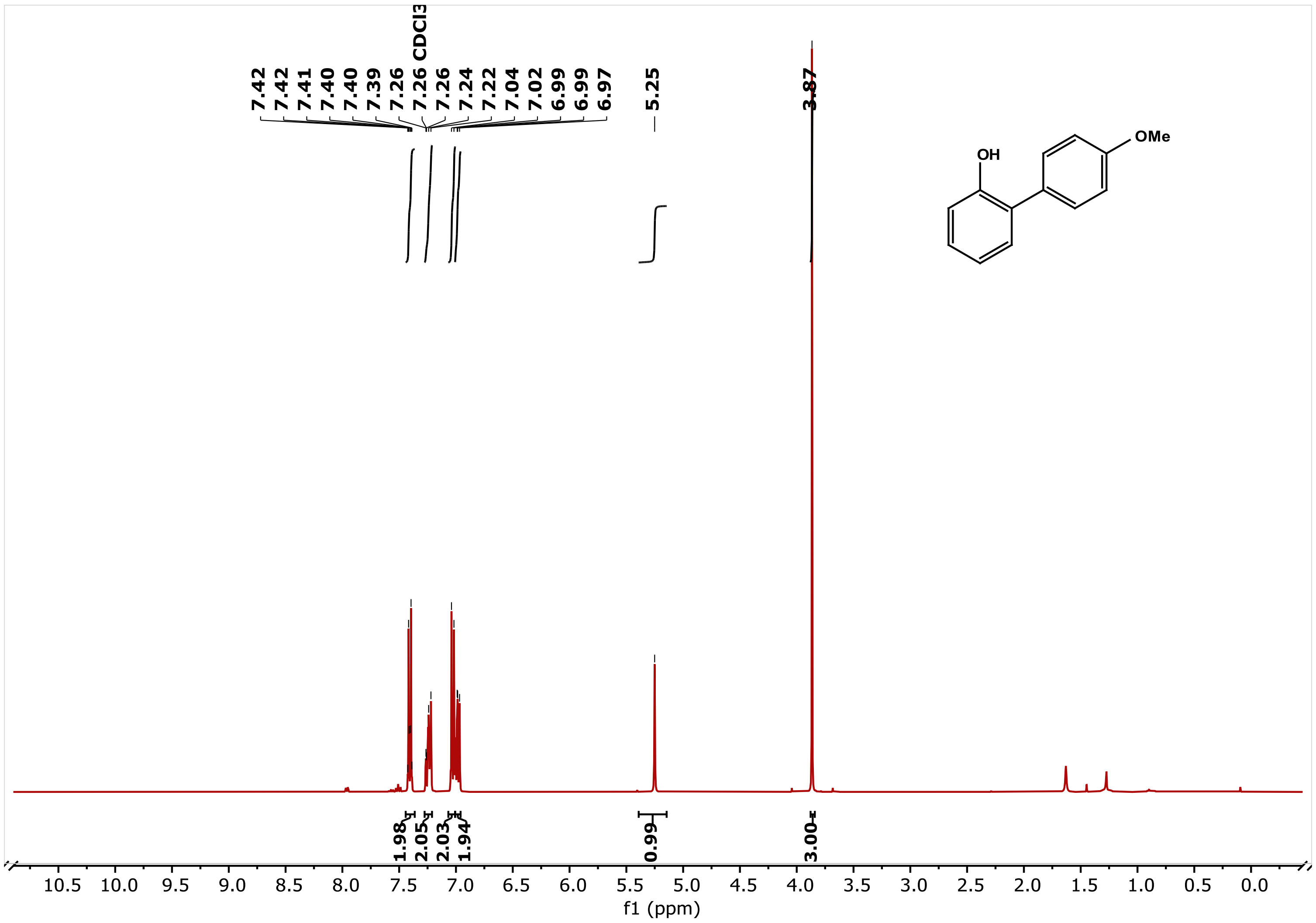

^{13}C{^{1}H} NMR (101 MHz, $CDCl_3$):

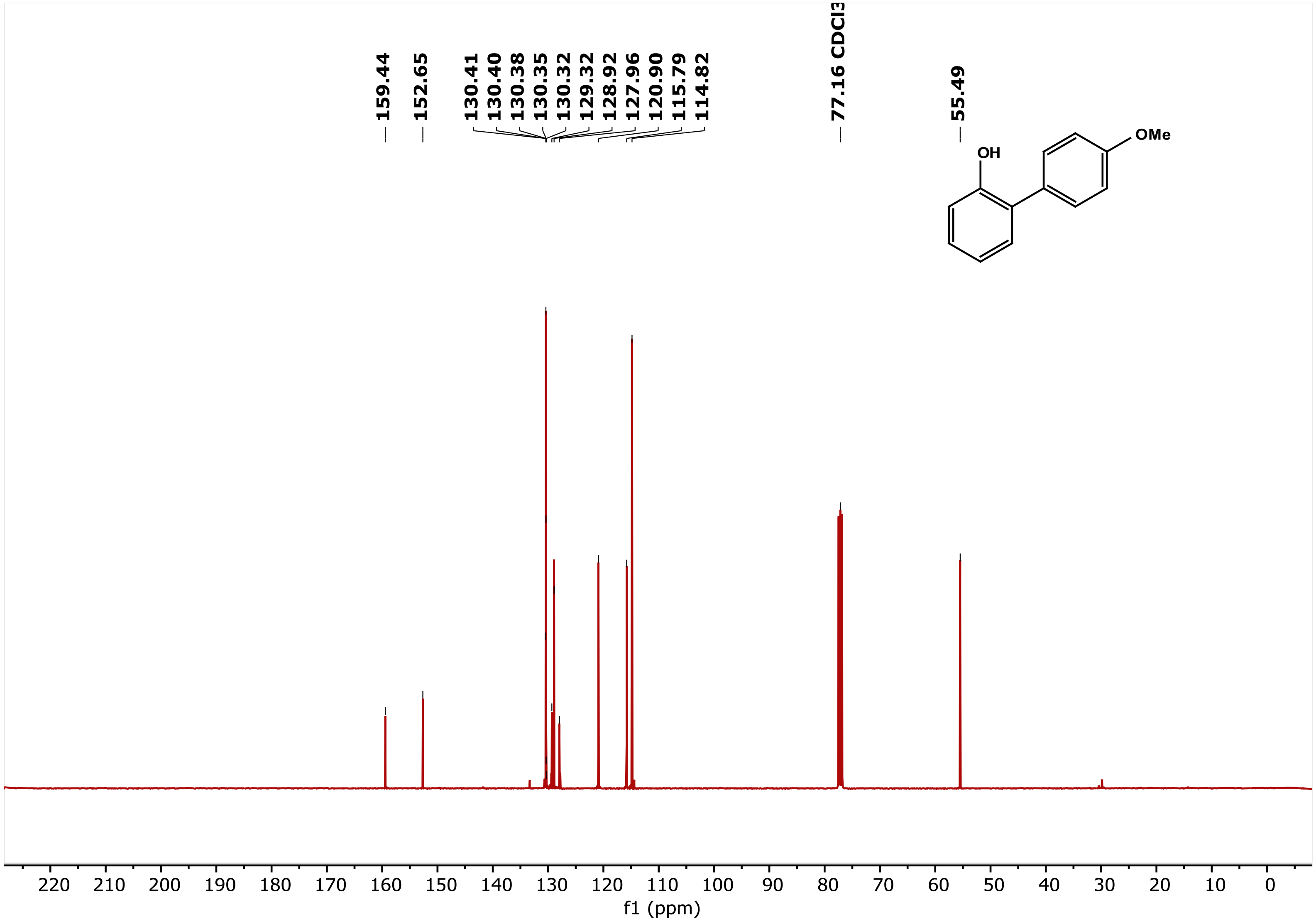

4'-Methyl-[1,1'-biphenyl]-2-ol (7)

^{1}H NMR (400 MHz, $CDCl_3$):

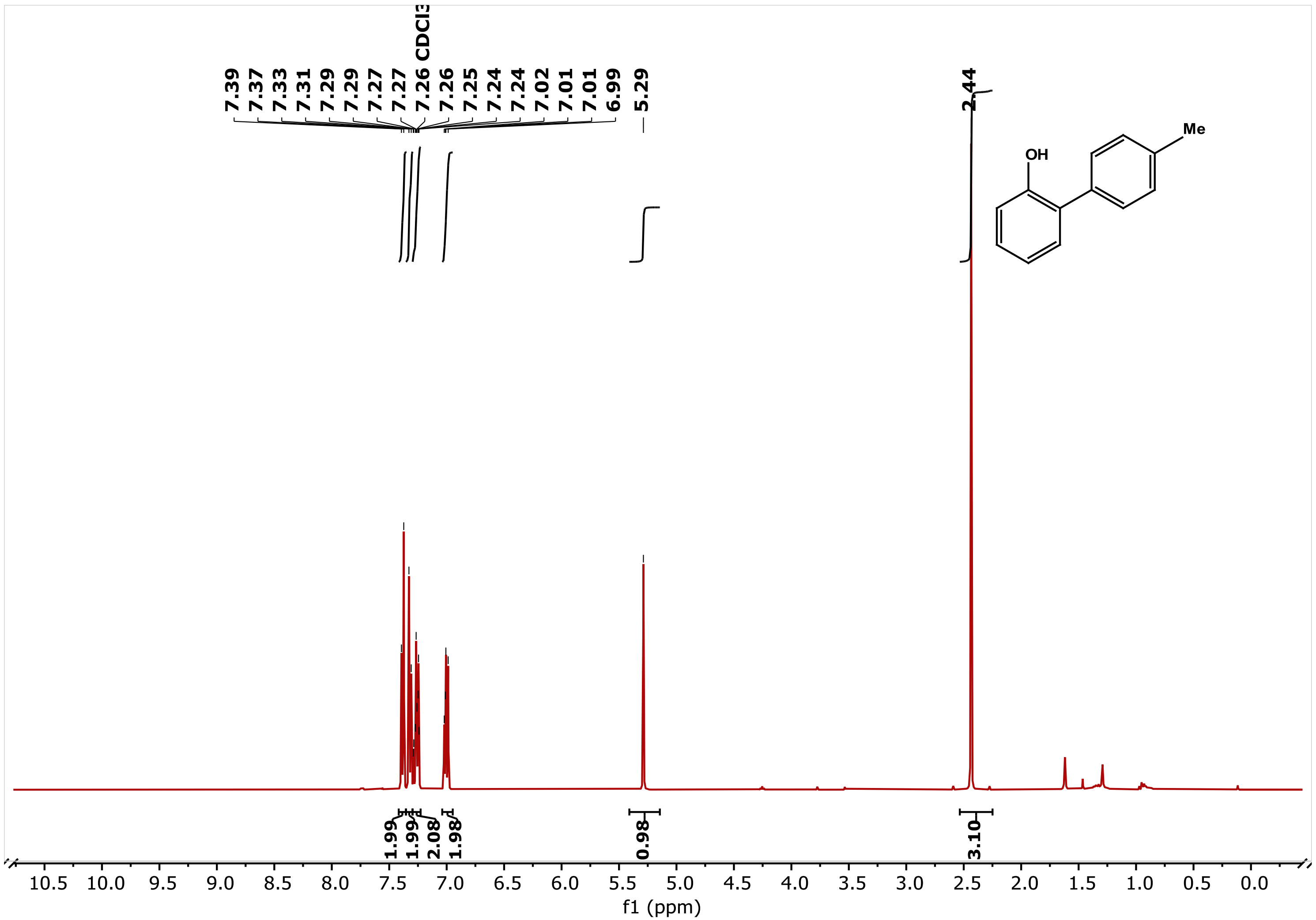

^{13}C{^{1}H} NMR (MHz, CDCl$_3$):

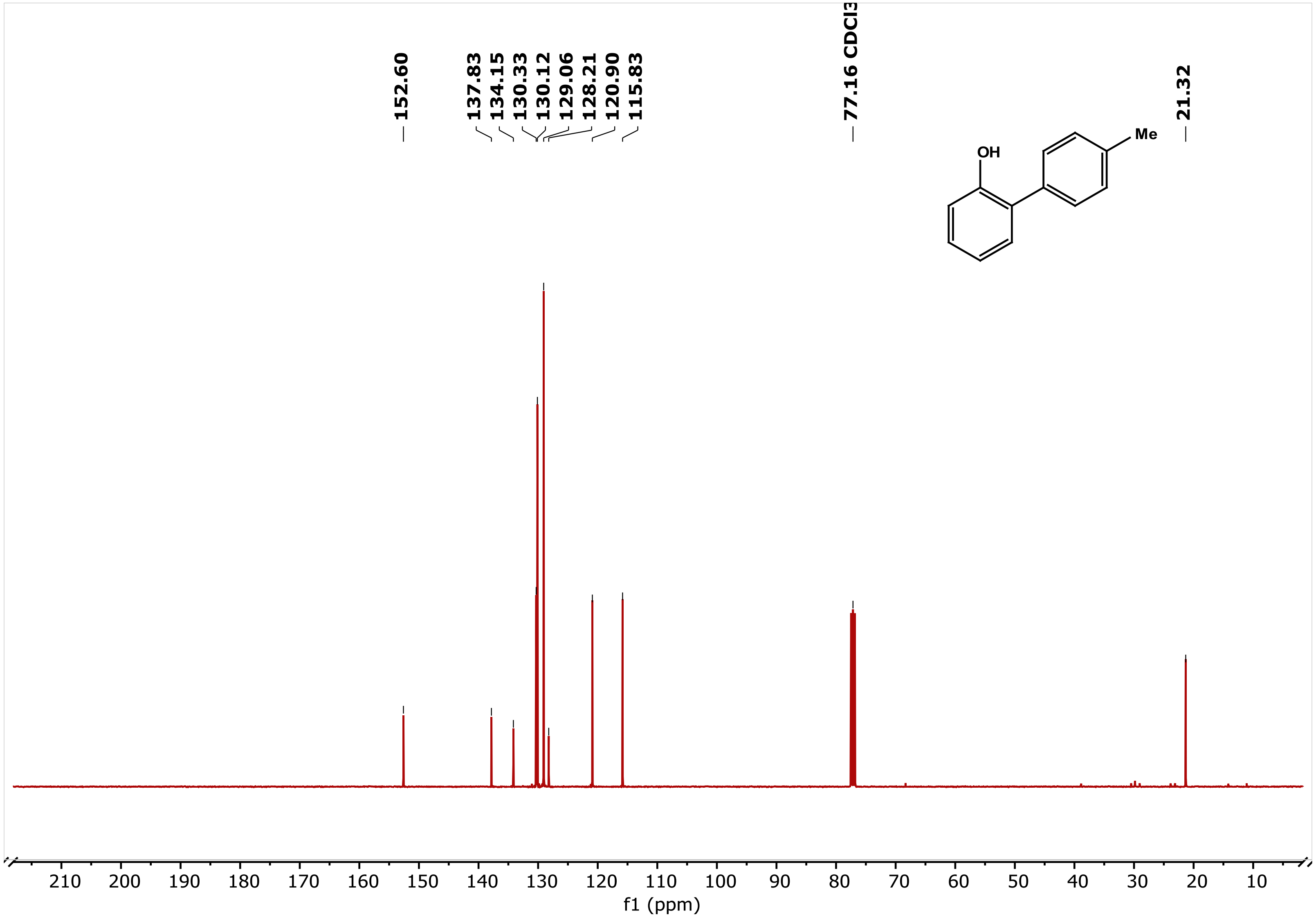

4,4''-dimethyl-[1,1':3',1''-terphenyl]-2'-ol

¹H NMR (400 MHz, $CDCl_3$):

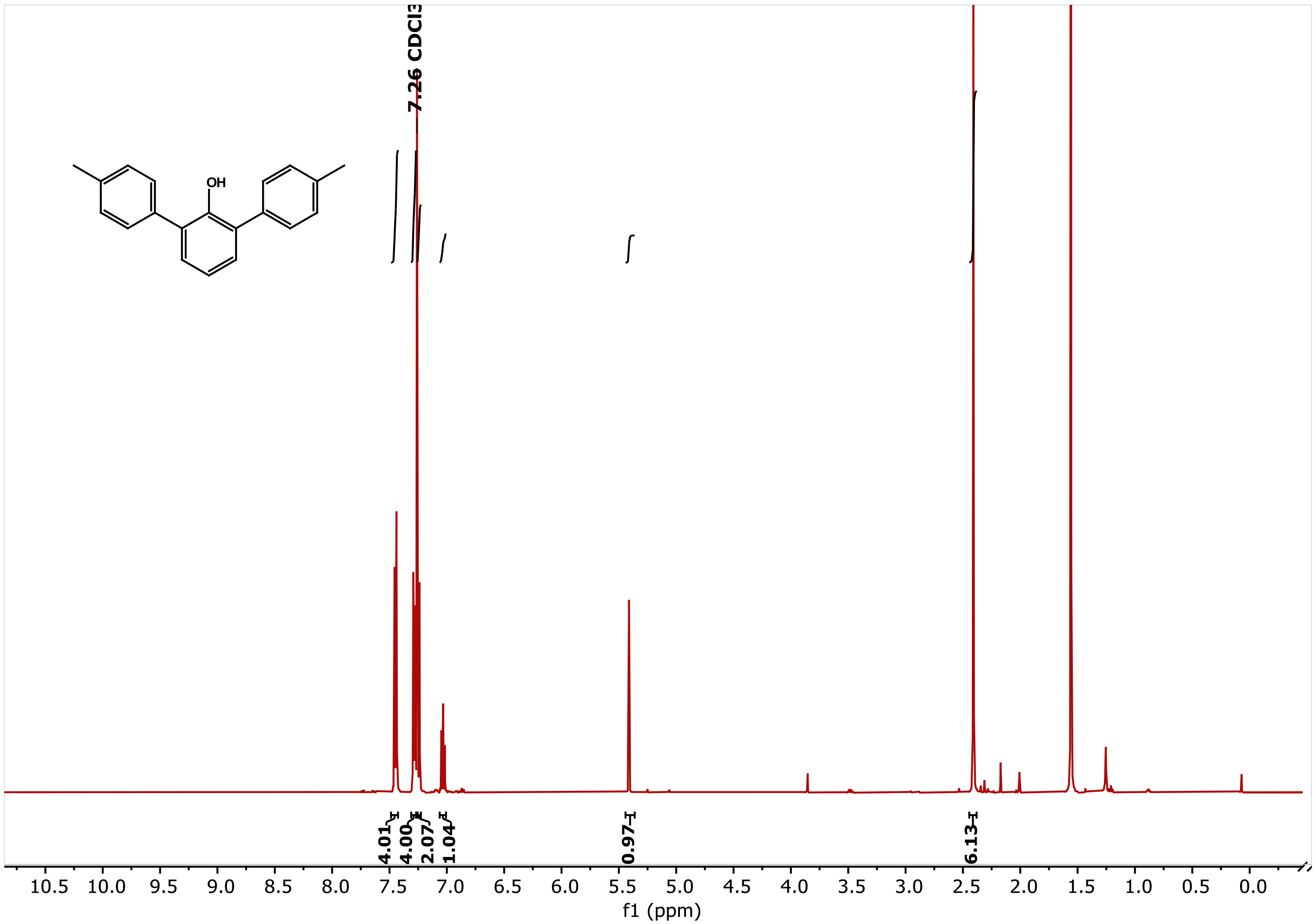

^{13}C{^{1}H} NMR (MHz, $CDCl_3$):

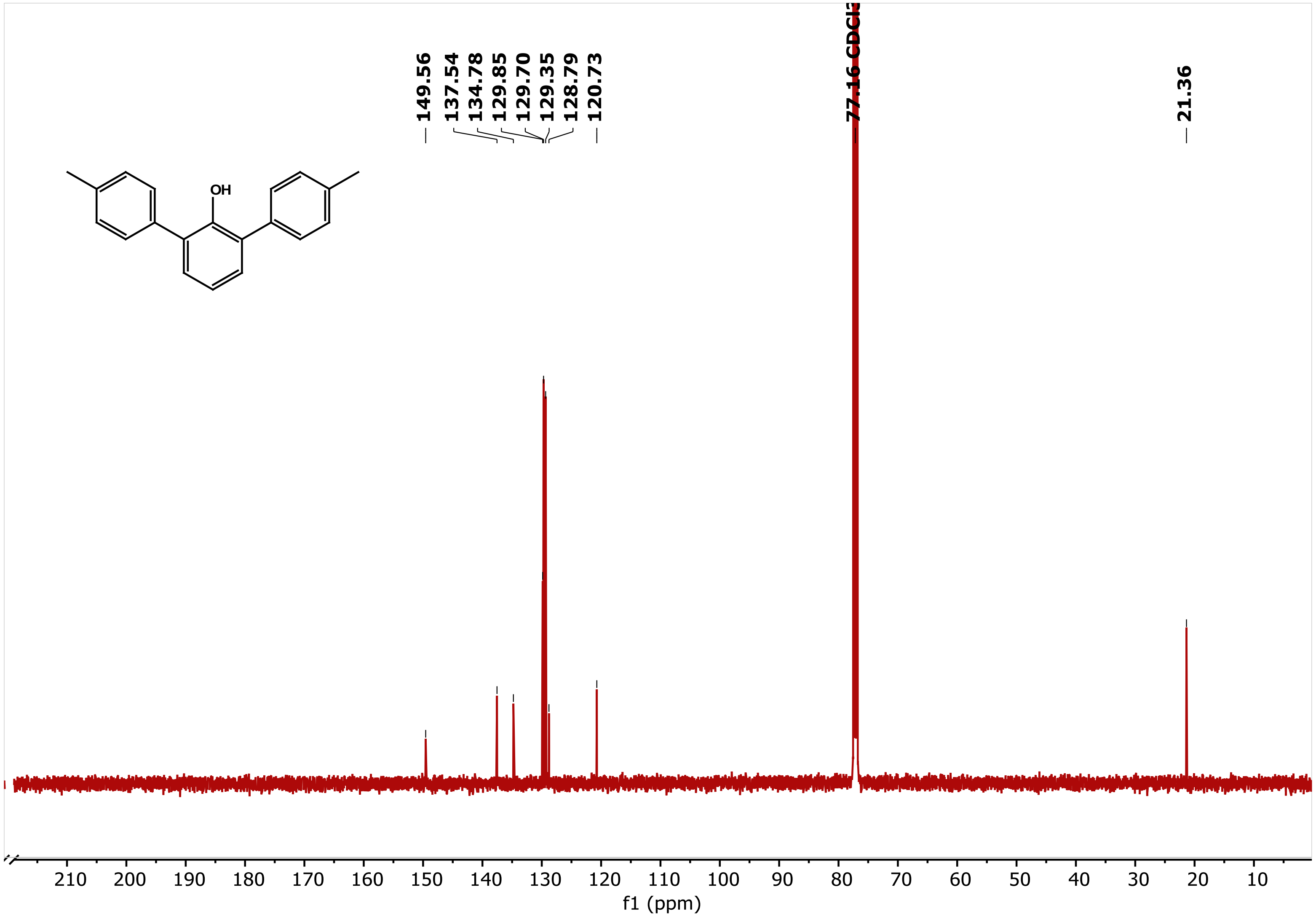

3'-Ethoxy-[1,1'-biphenyl]-2-ol (8)

¹H NMR ($CDCl_3$, 500 MHz):

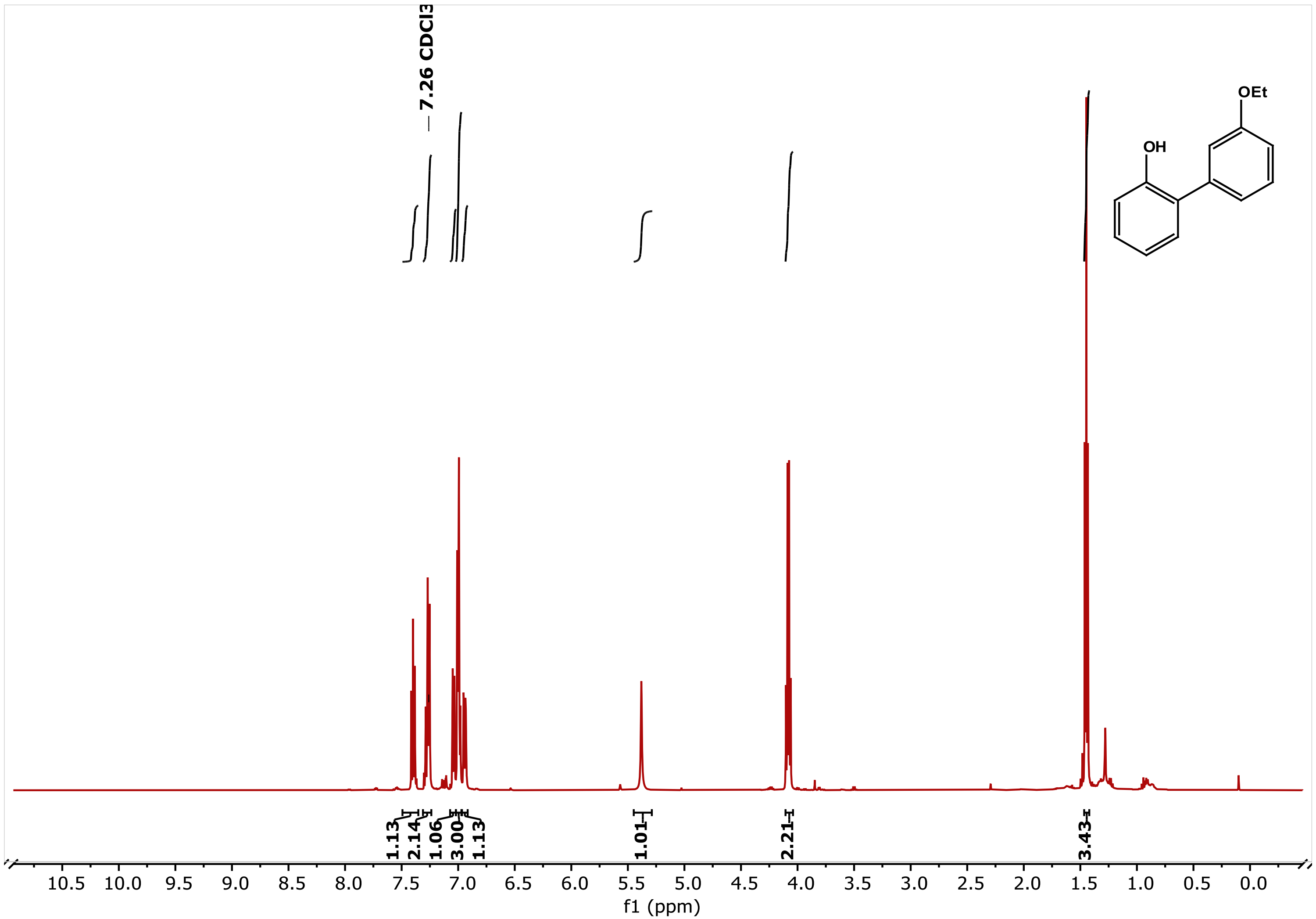

^{13}C{^{1}H} NMR (126 MHz, $CDCl_3$):

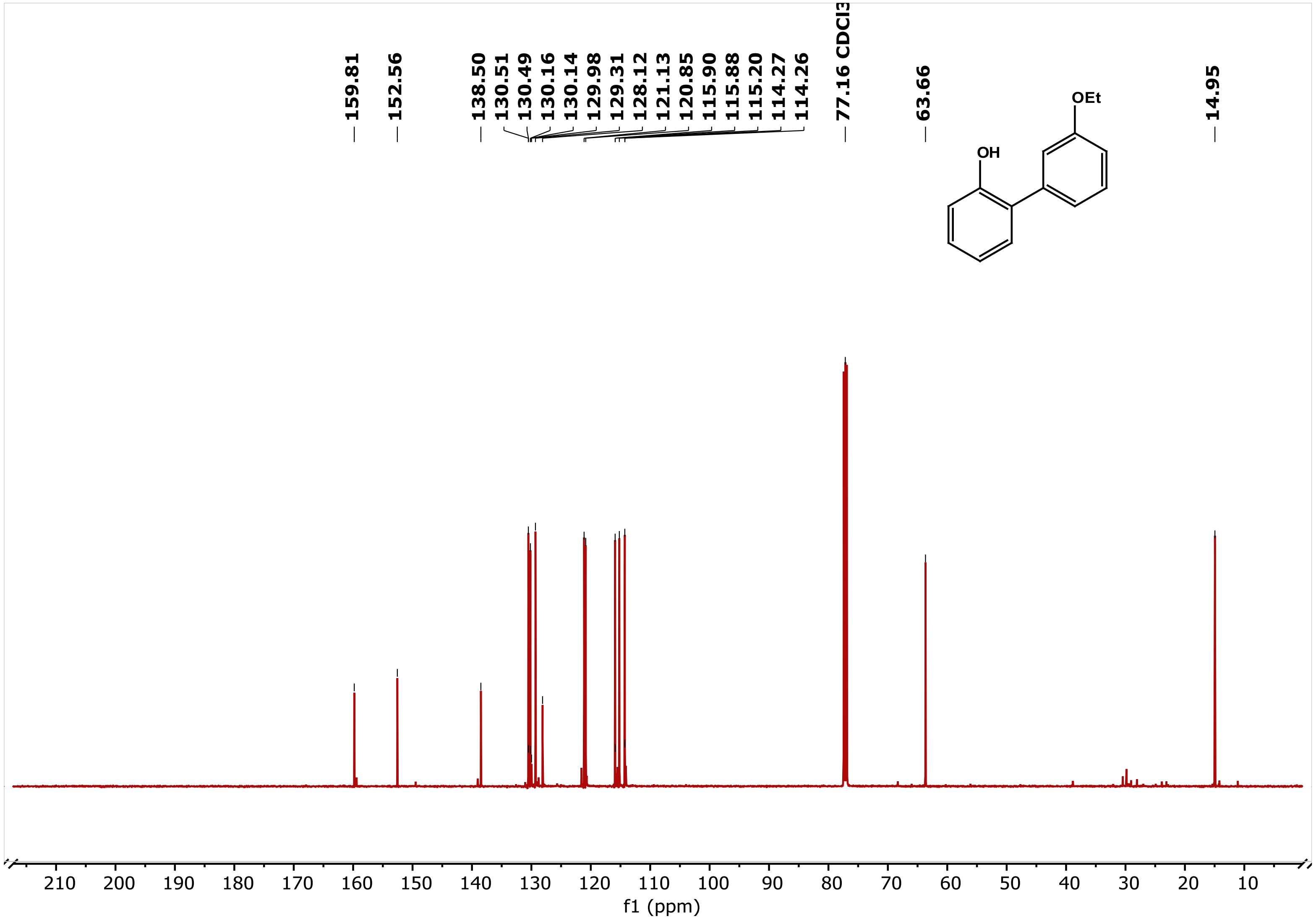

3'-Bromo-[1,1'-biphenyl]-2-ol (9)

^{1}H NMR ($CDCl_3$, 400 MHz):

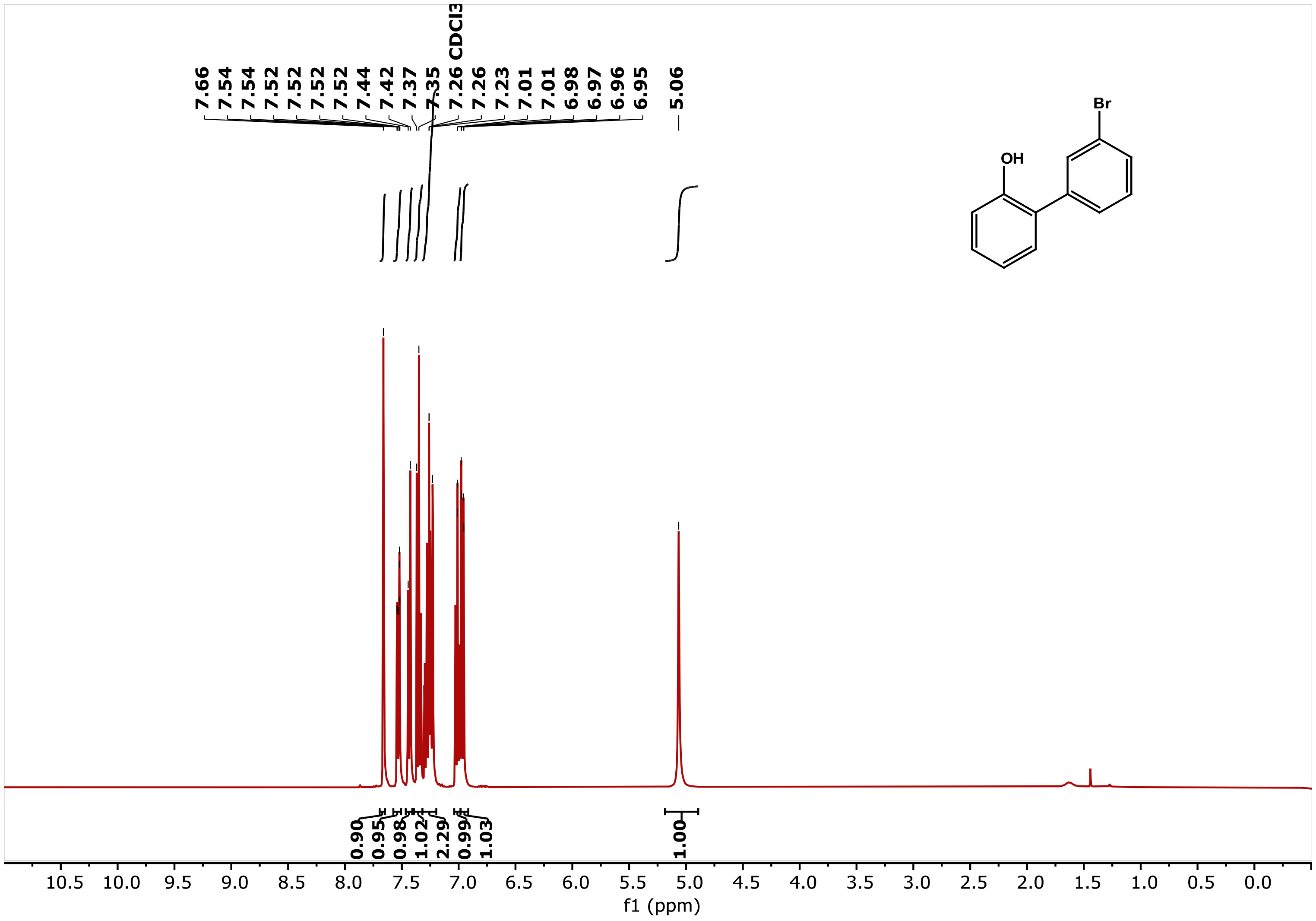

^{13}C{^{1}H} NMR (101 MHz, $CDCl_3$):

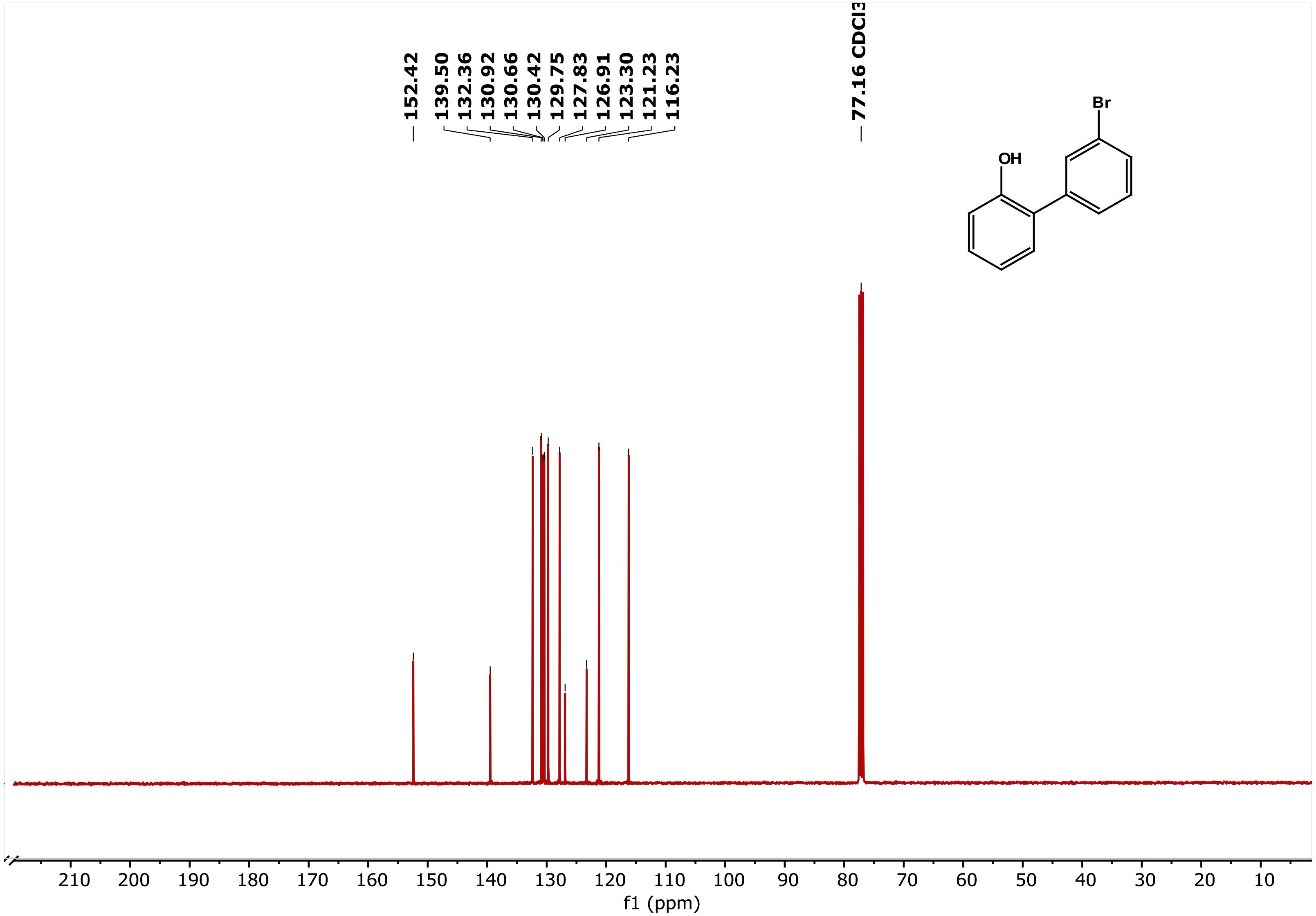

4'-(Trifluoromethoxy)-[1,1'-biphenyl]-2-ol (10)

^{1}H NMR ($CDCl_3$, 500 MHz):

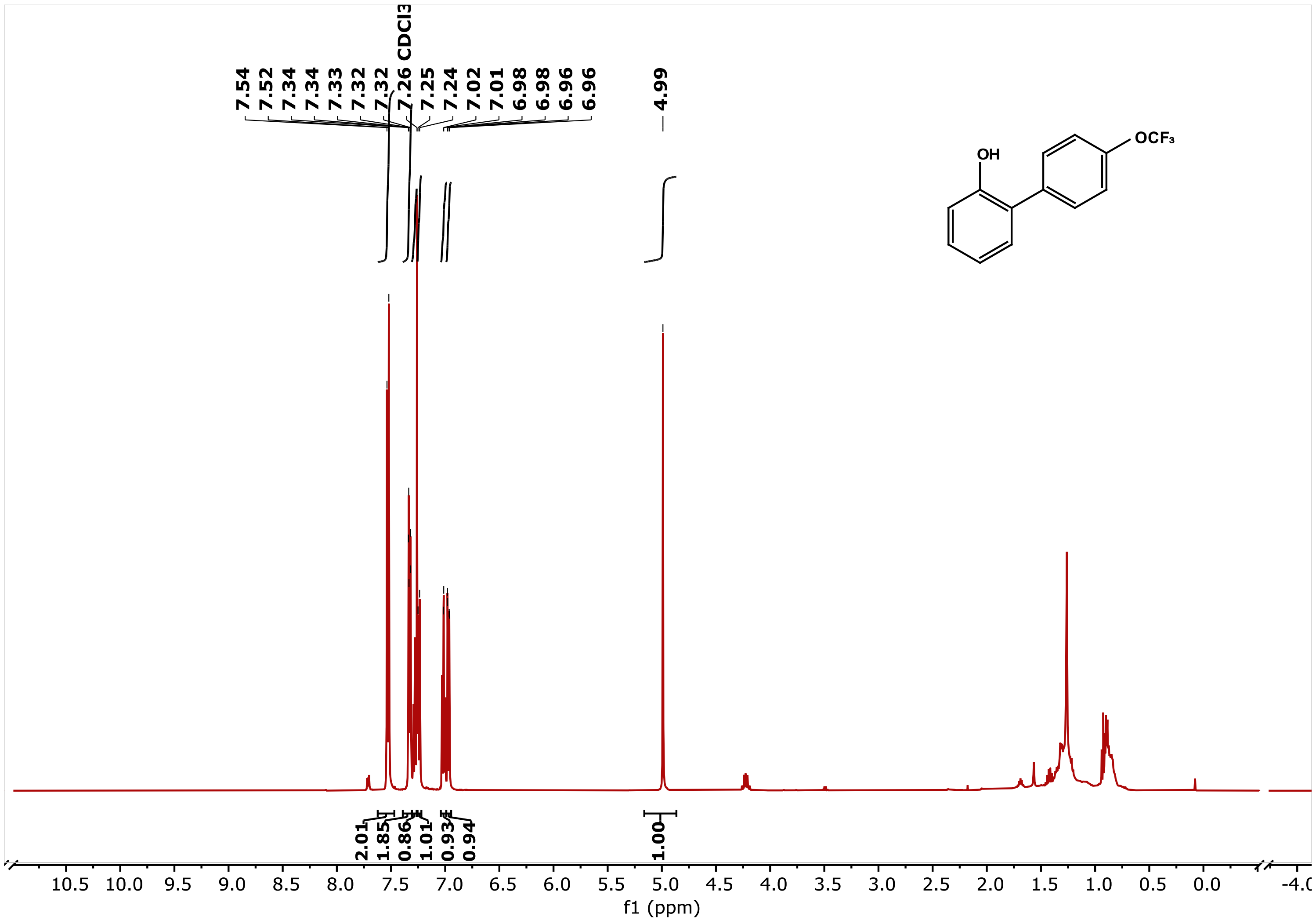

$^{13}C\{^1H\}$ NMR (126 MHz, $CDCl_3$):

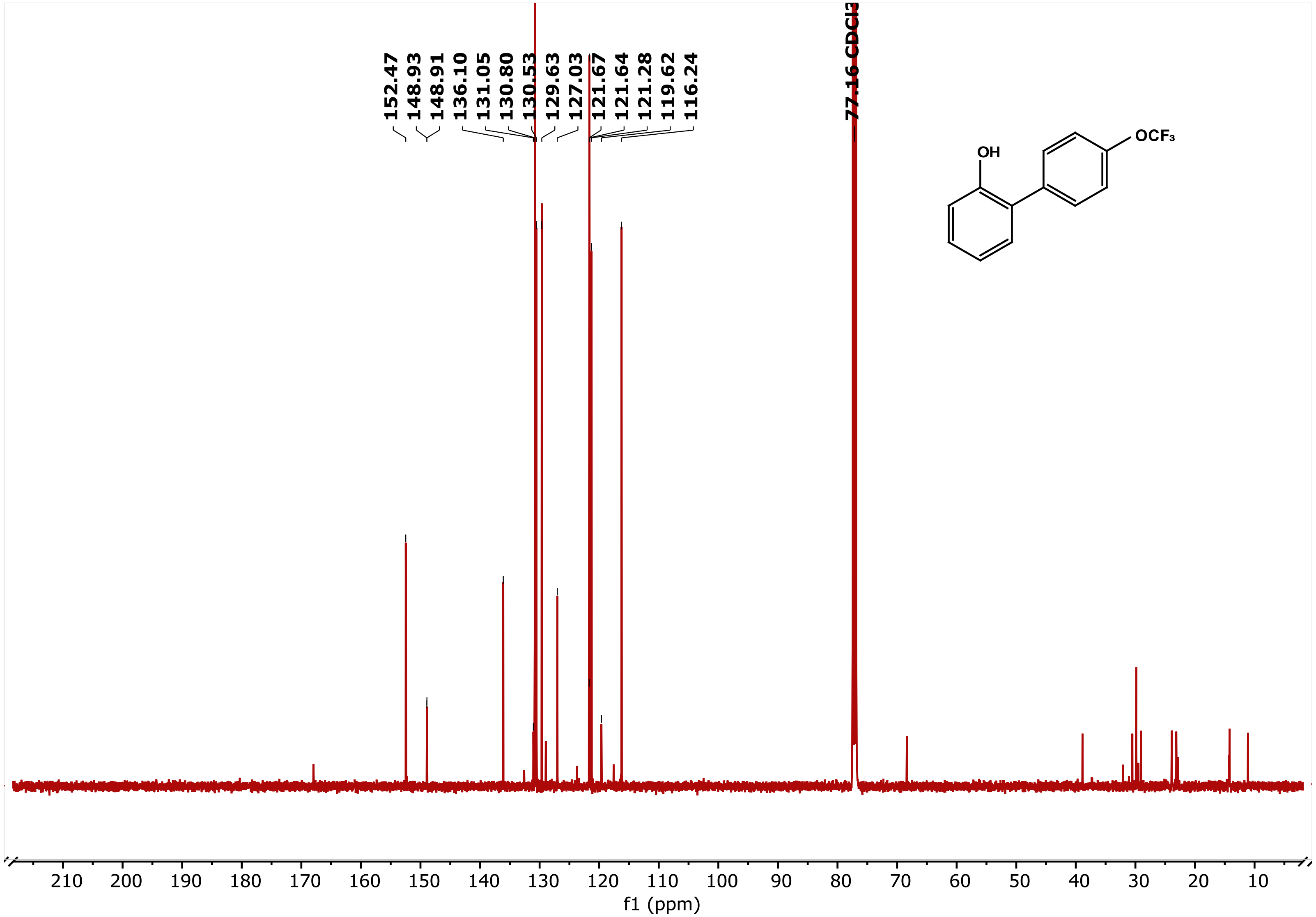

^{19}F NMR (377 MHz, $CDCl_3$):

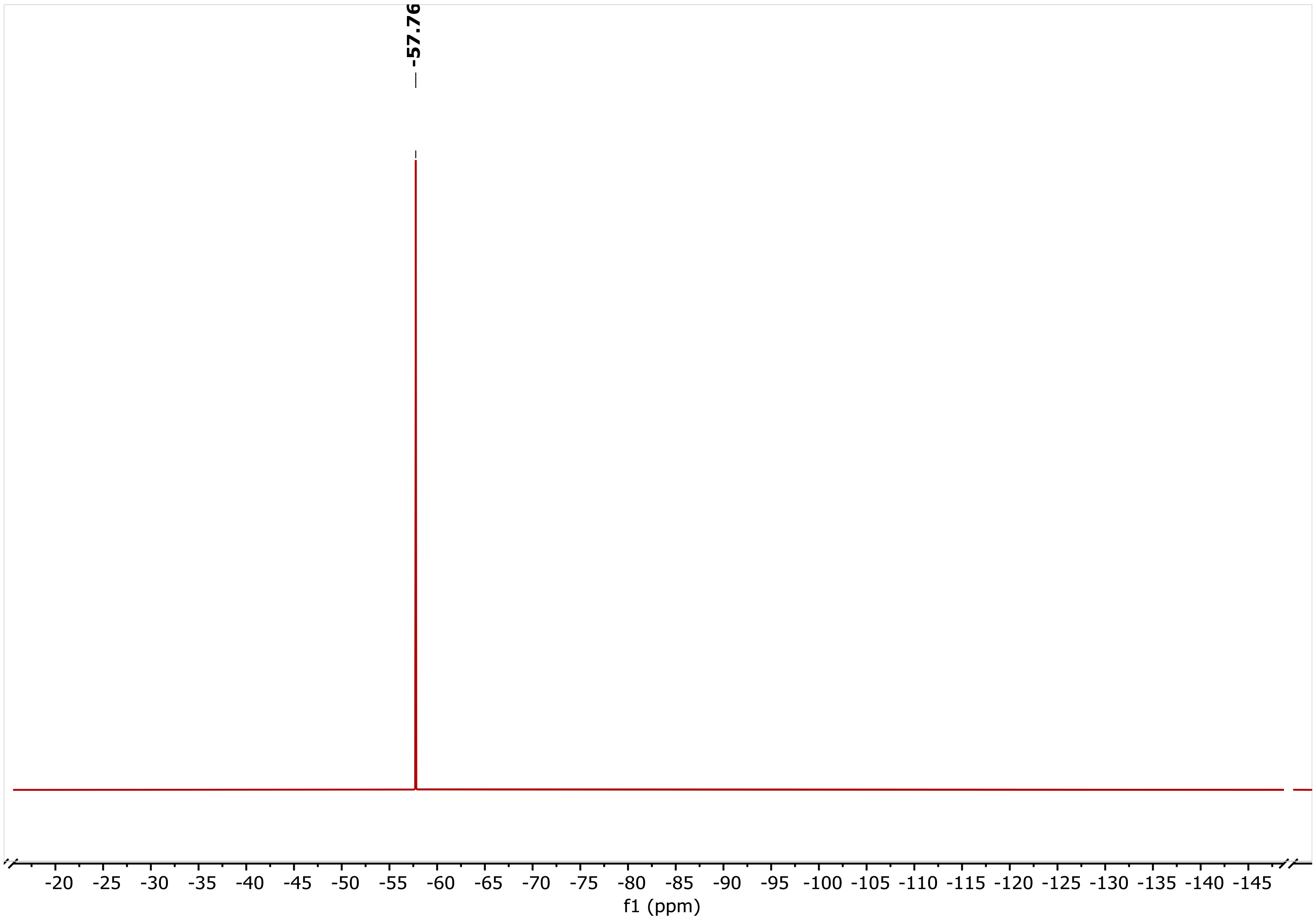

4'-(Trifluoromethyl)-[1,1'-biphenyl]-2-ol (11)

^{1}H NMR (400 MHz, $CDCl_3$):

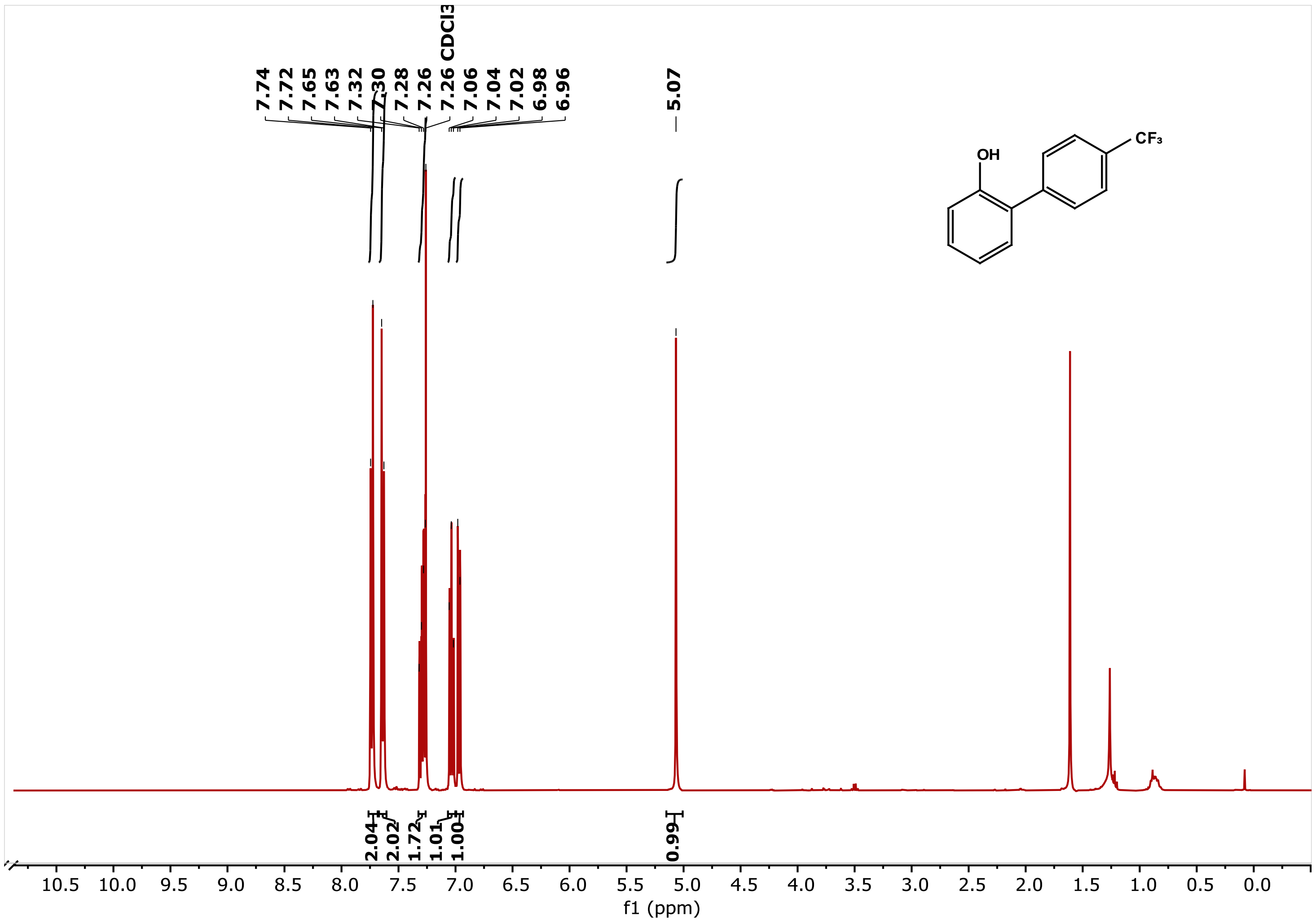

^{13}C{^{1}H} NMR (126 MHz, $CDCl_3$):

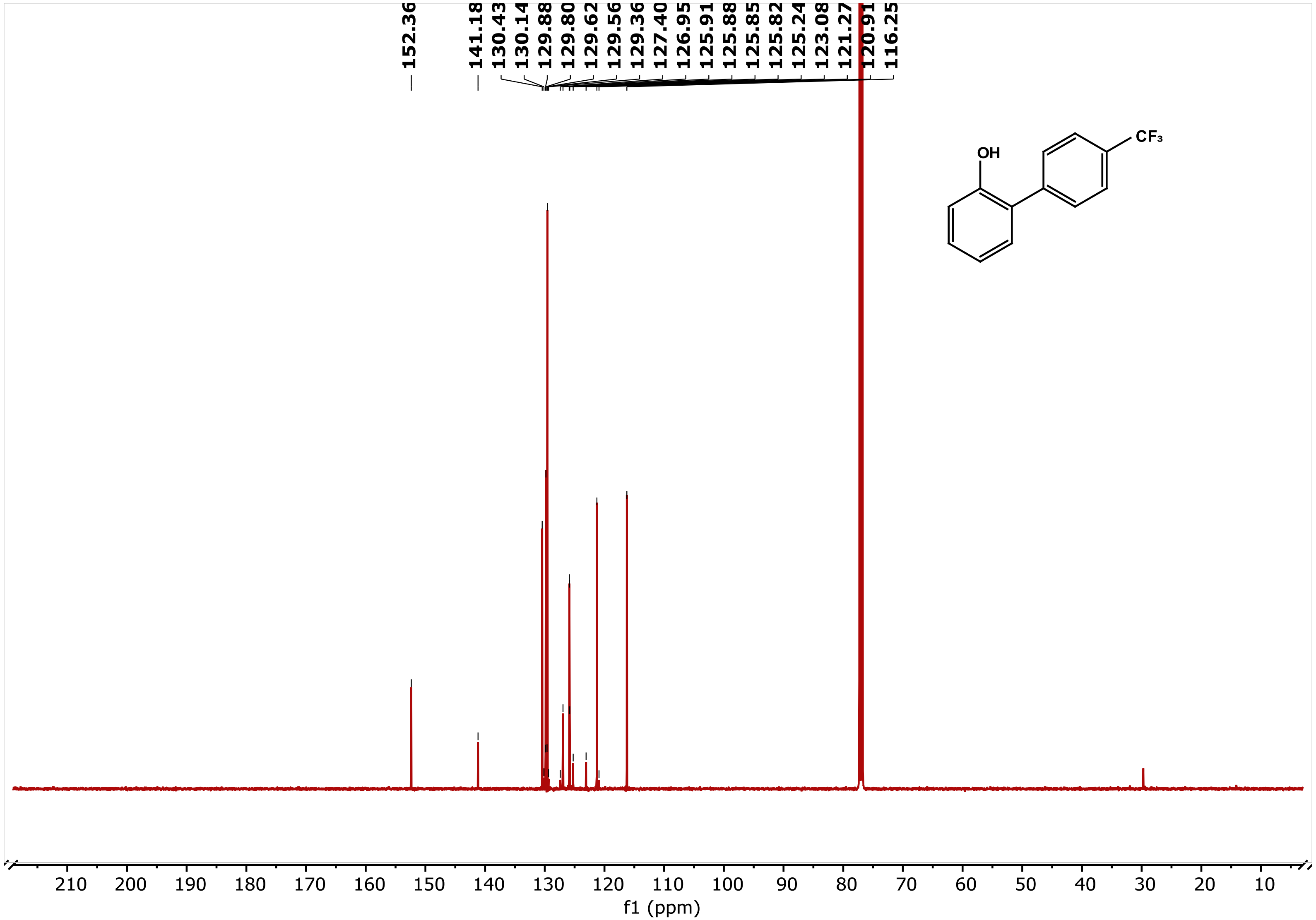

^{19}F NMR (376 MHz, $CDCl_3$):

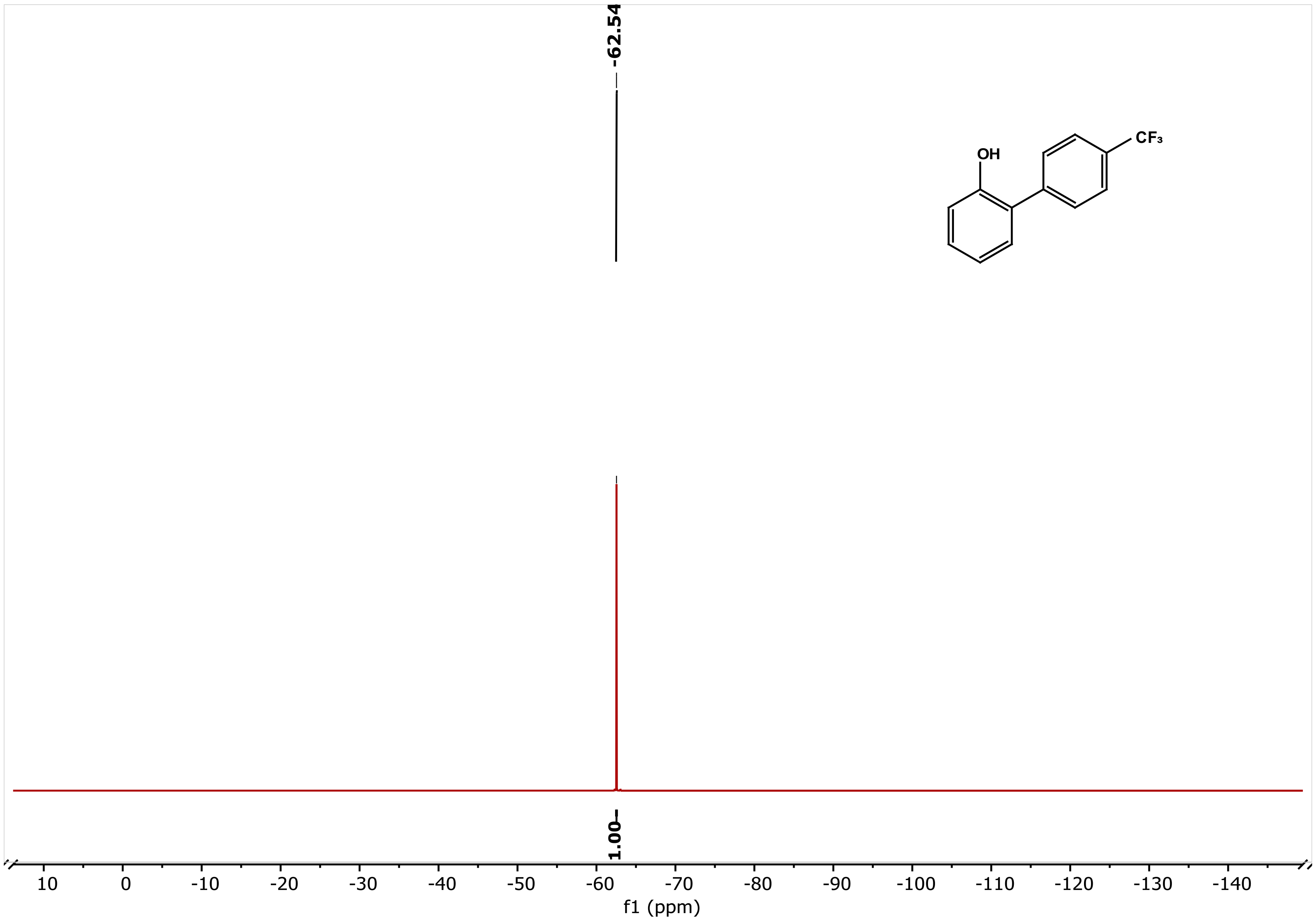

2'-Hydroxy-[1,1'-biphenyl]-4-carbonitrile (12)

^{1}H NMR (400 MHz, $CDCl_3$):

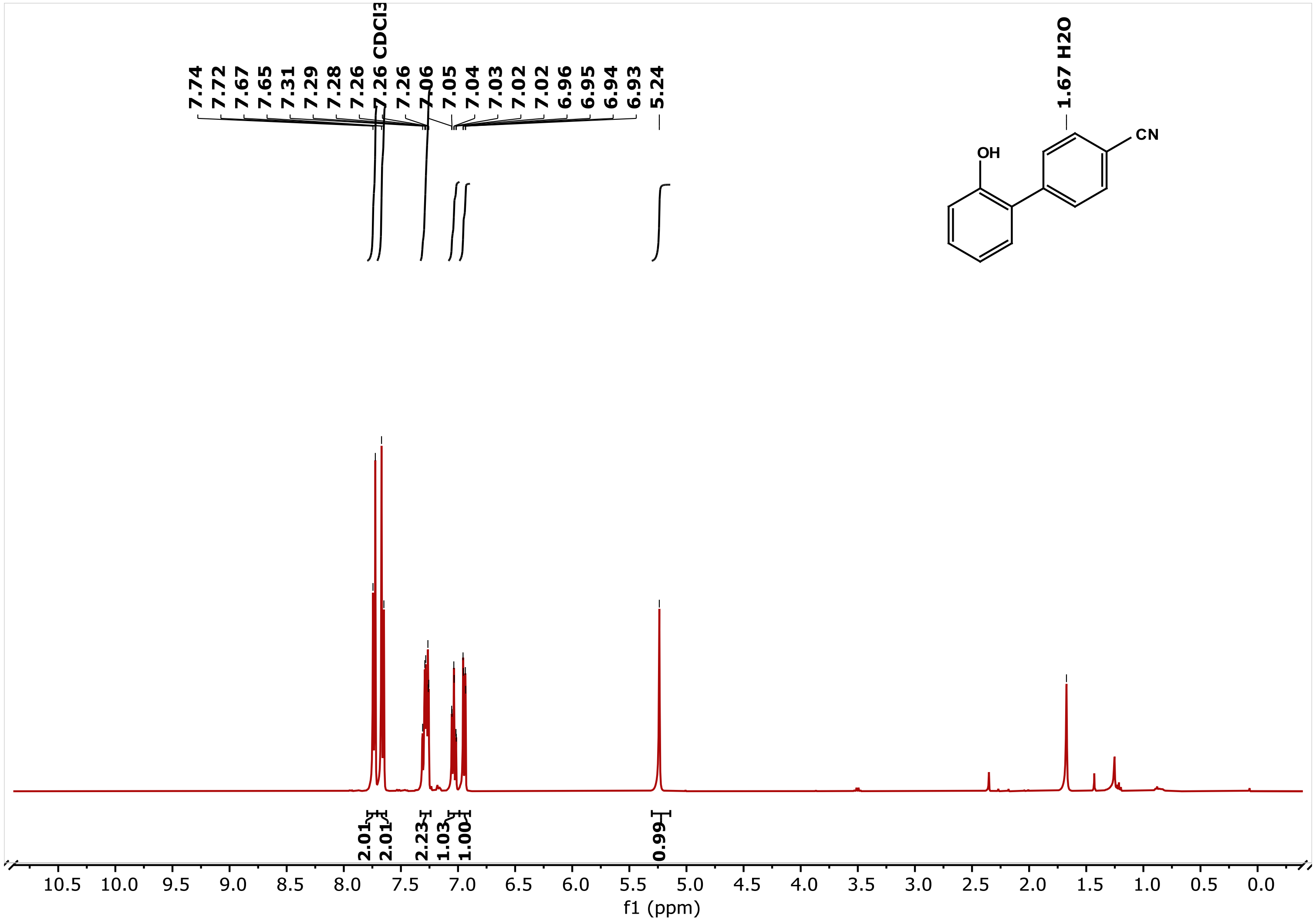

$^{13}C\{^1H\}$ NMR (101 MHz, $CDCl_3$):

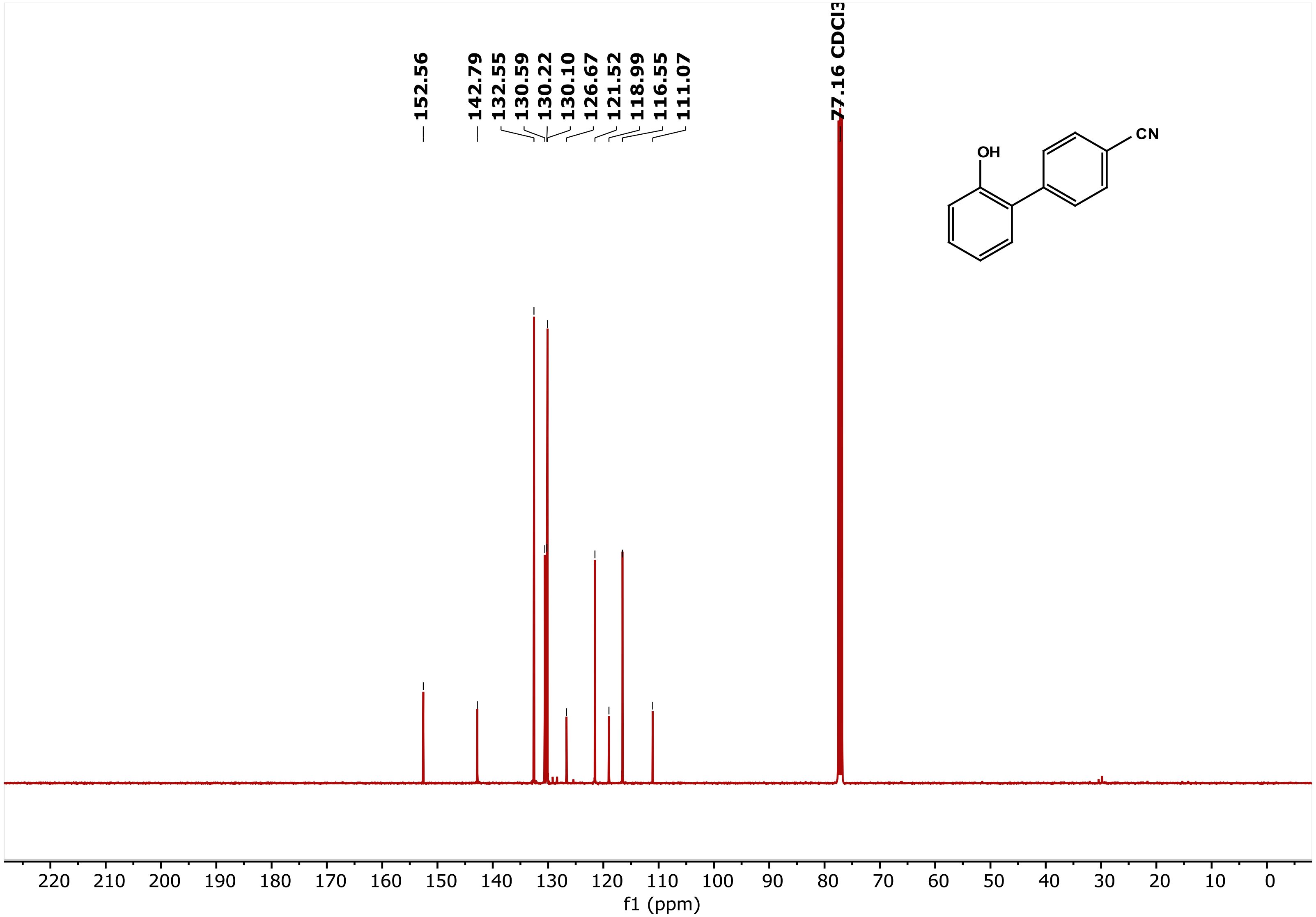

2-(Naphthalen-1-yl)phenol (13)

^{1}H NMR (400 MHz, $CDCl_3$):

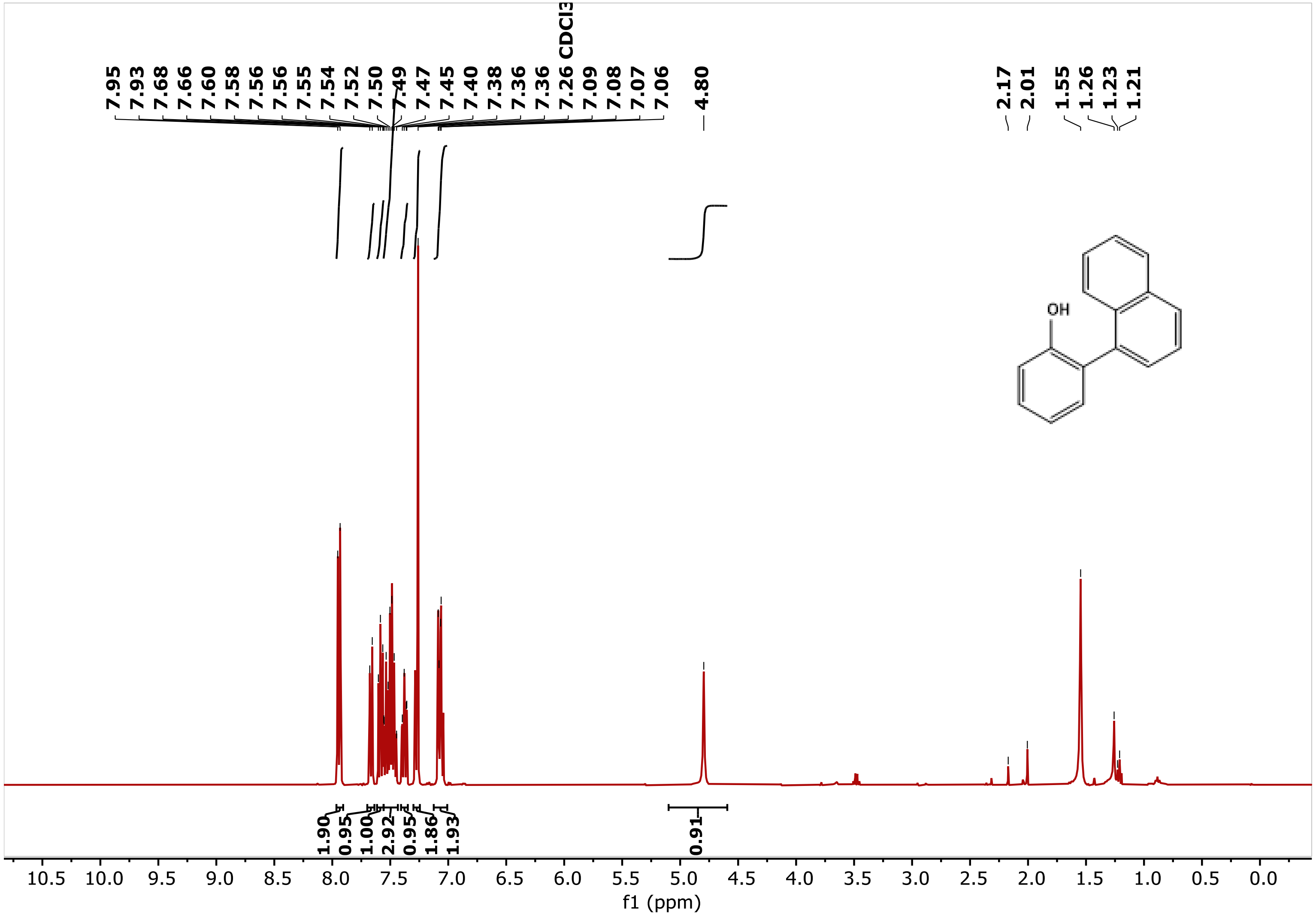

$^{13}C\{^1H\}$ **NMR** (101 MHz, $CDCl_3$):

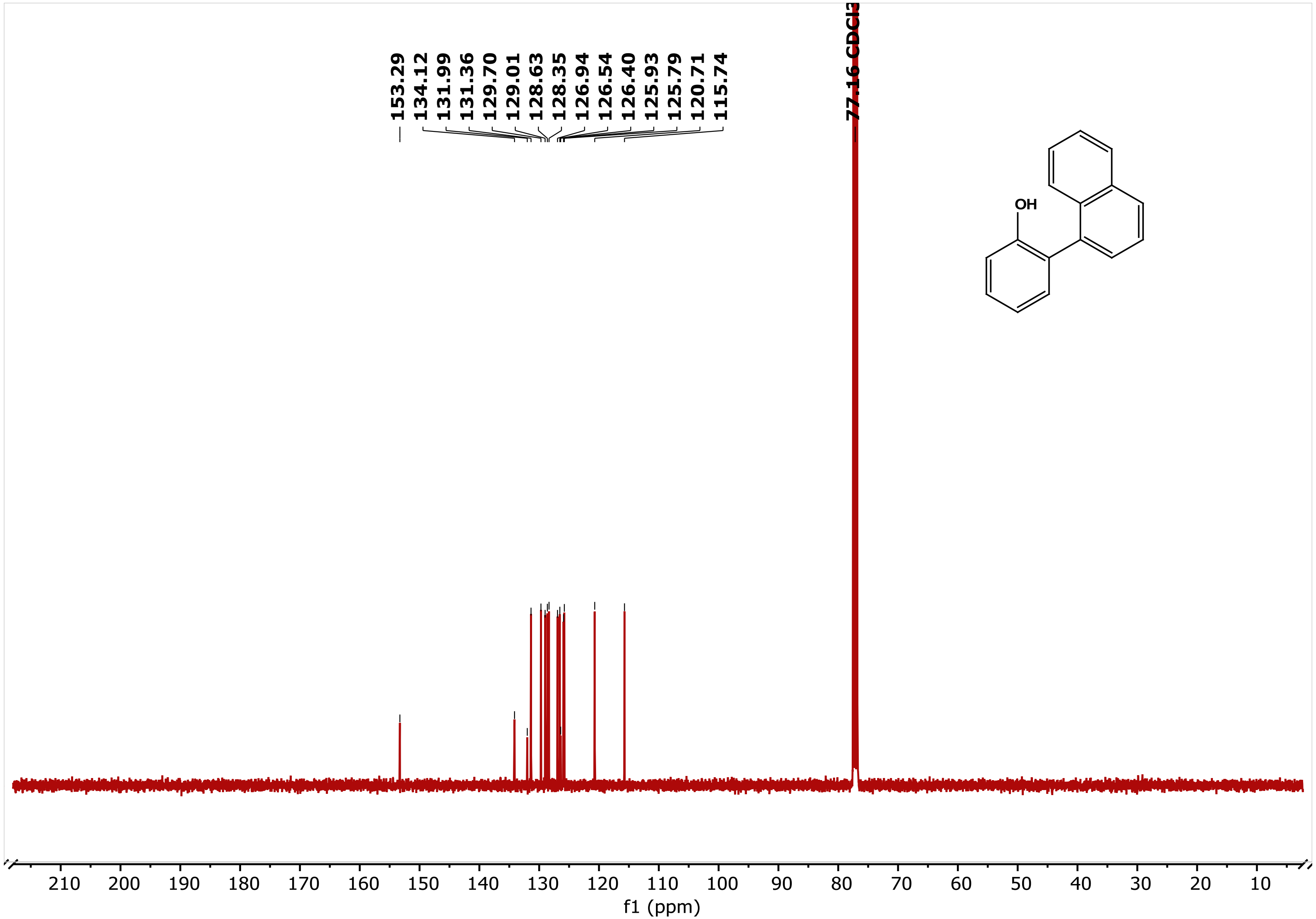

2-(6-Methoxypyridin-3-yl)phenol (14)

^{1}H NMR (DMSO, 400 MHz):

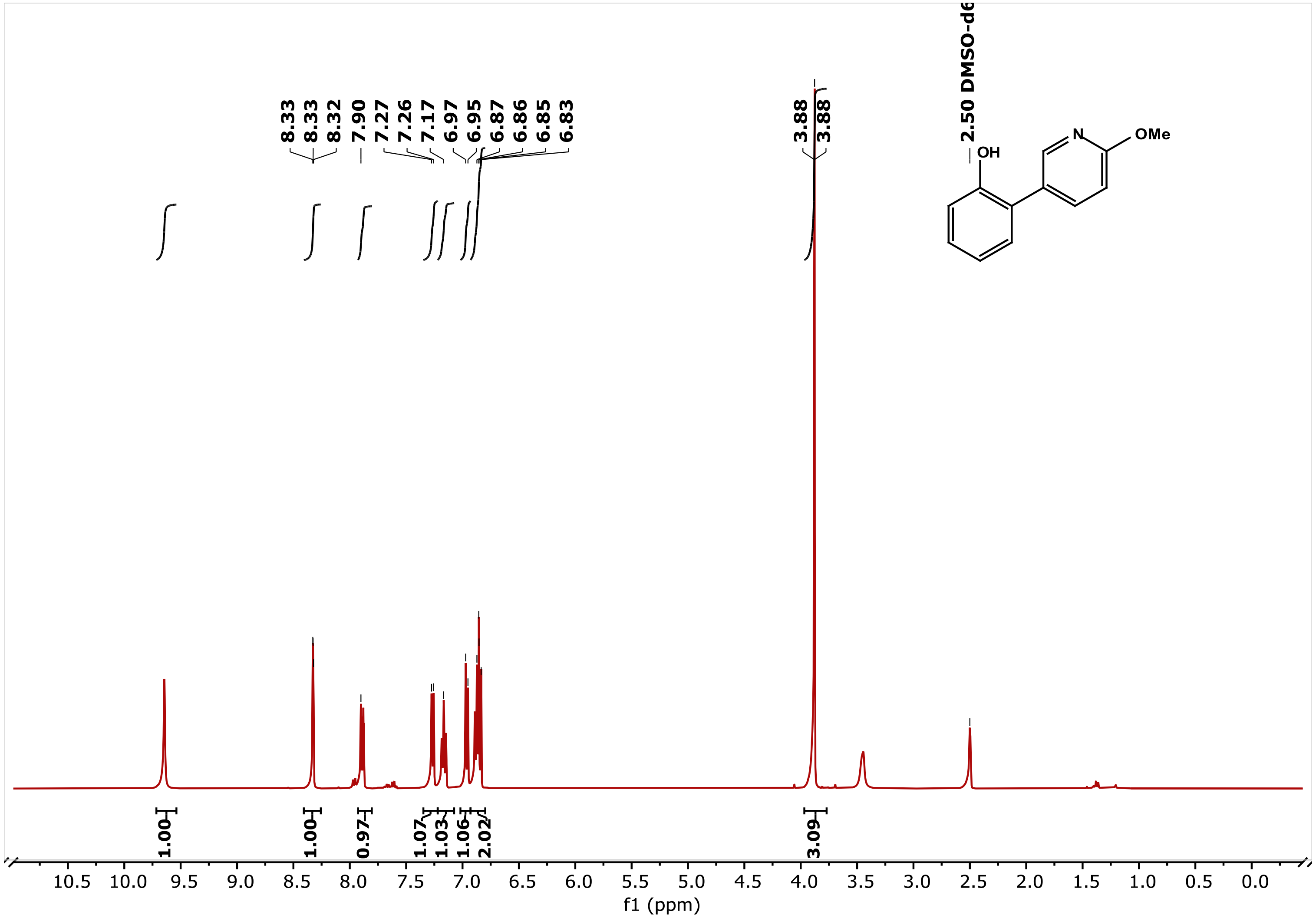

$^{13}C\{^1H\}$ NMR (DMSO, 101 MHz):

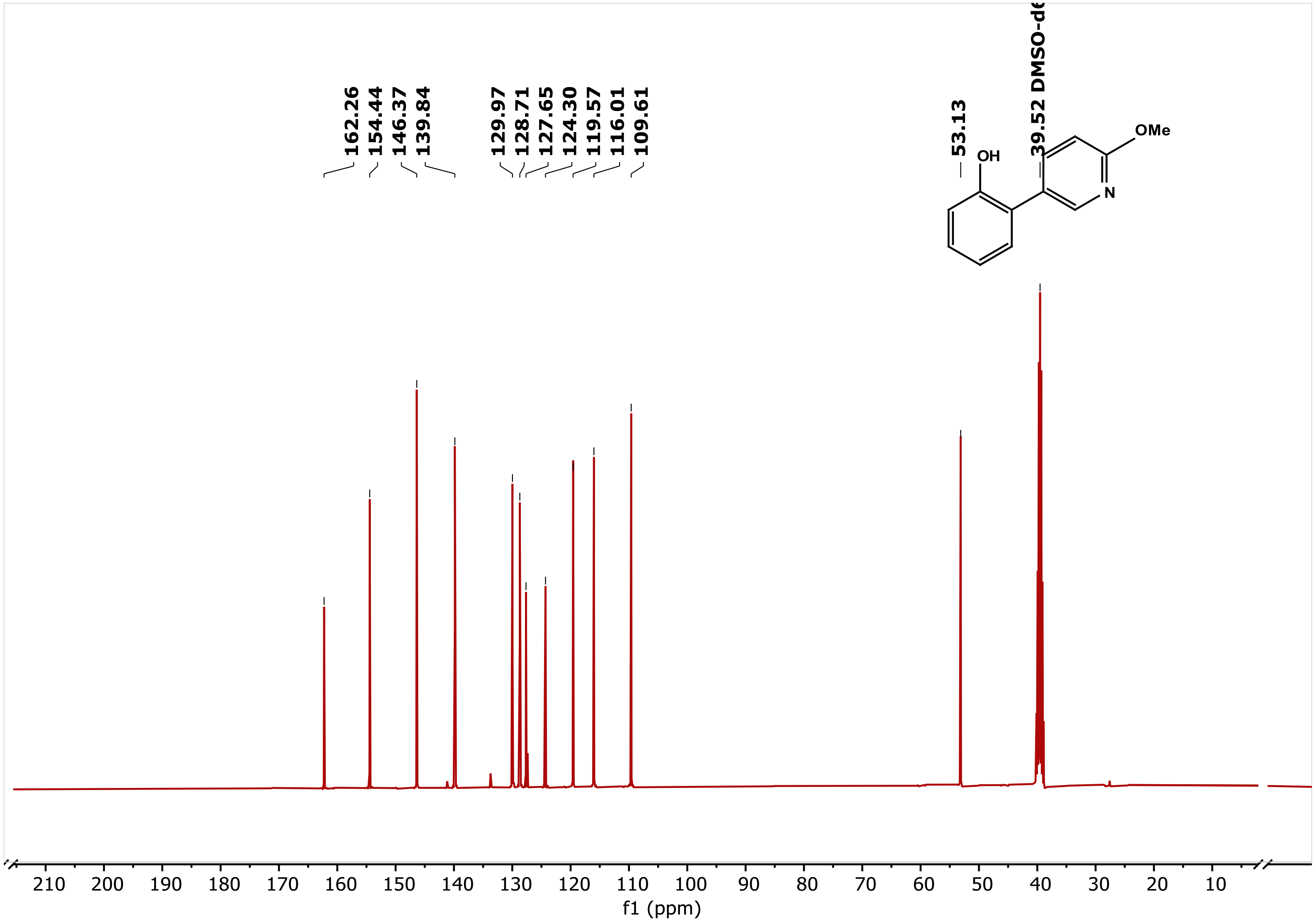

4'-Fluoro-3-methoxy-[1,1'-biphenyl]-2-ol (16)

¹H NMR (400 MHz, $CDCl_3$):

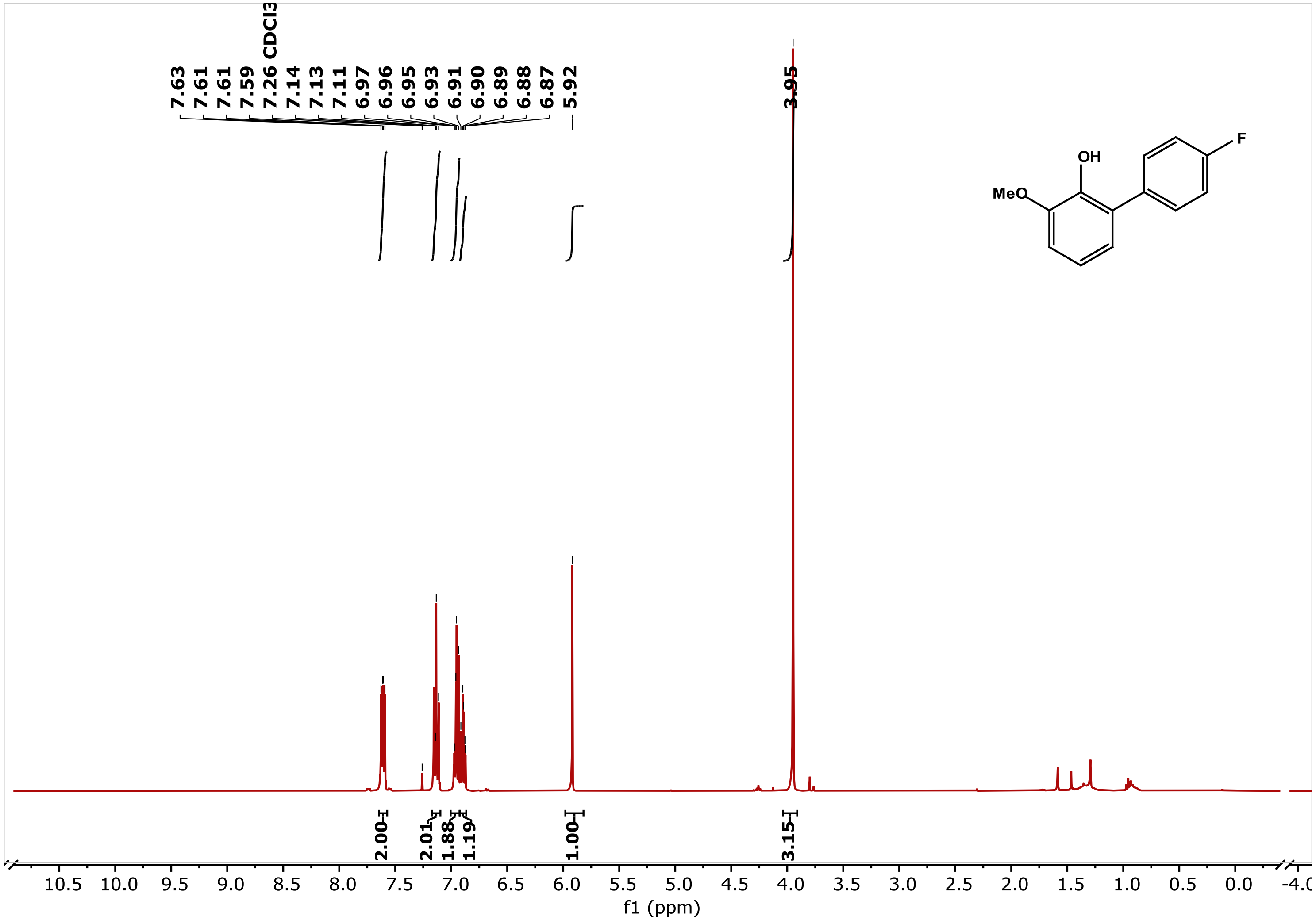

^{13}C{^{1}H} NMR ($CDCl_3$, 101 MHz):

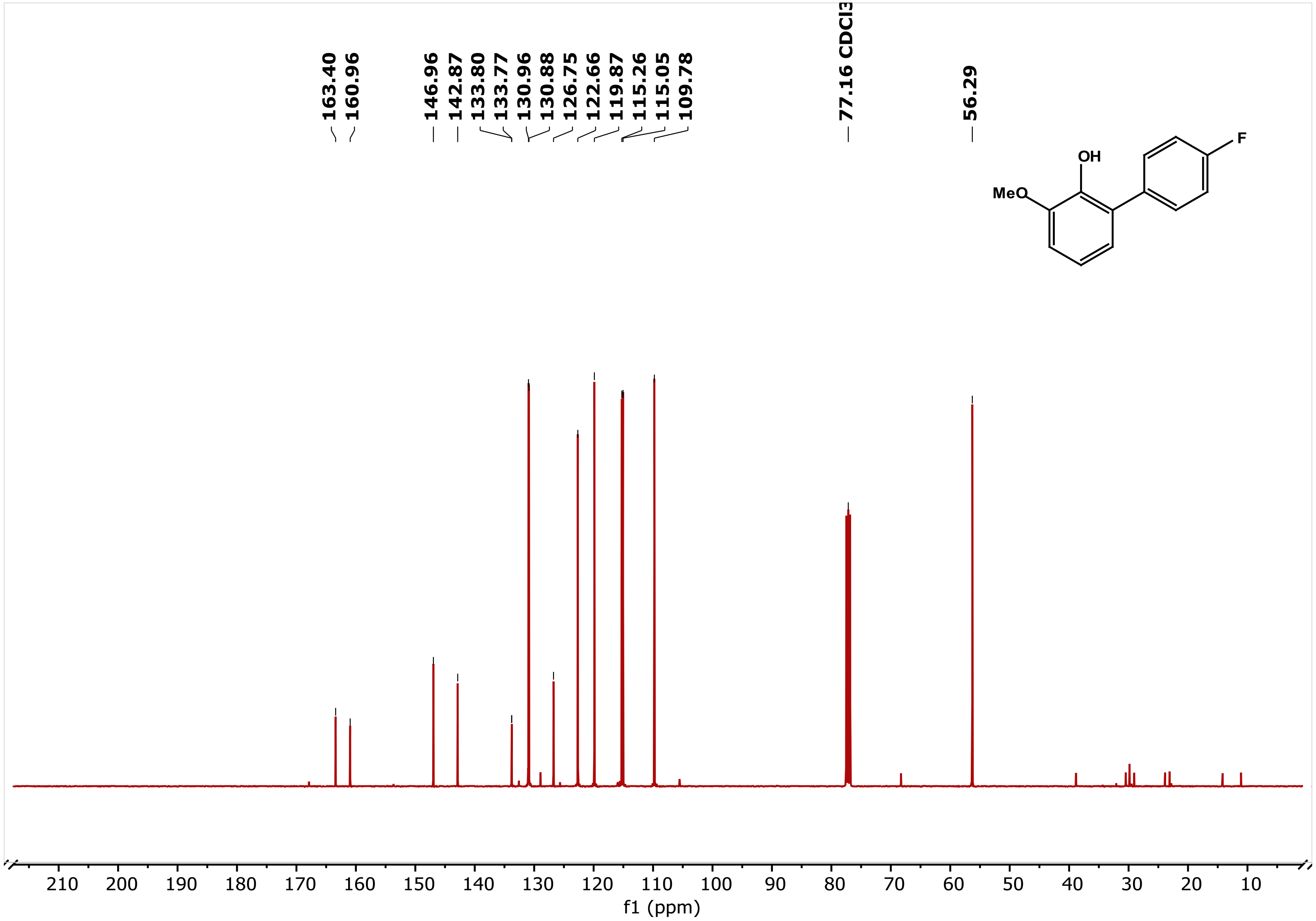

^{19}F NMR (377 MHz, $CDCl_3$):

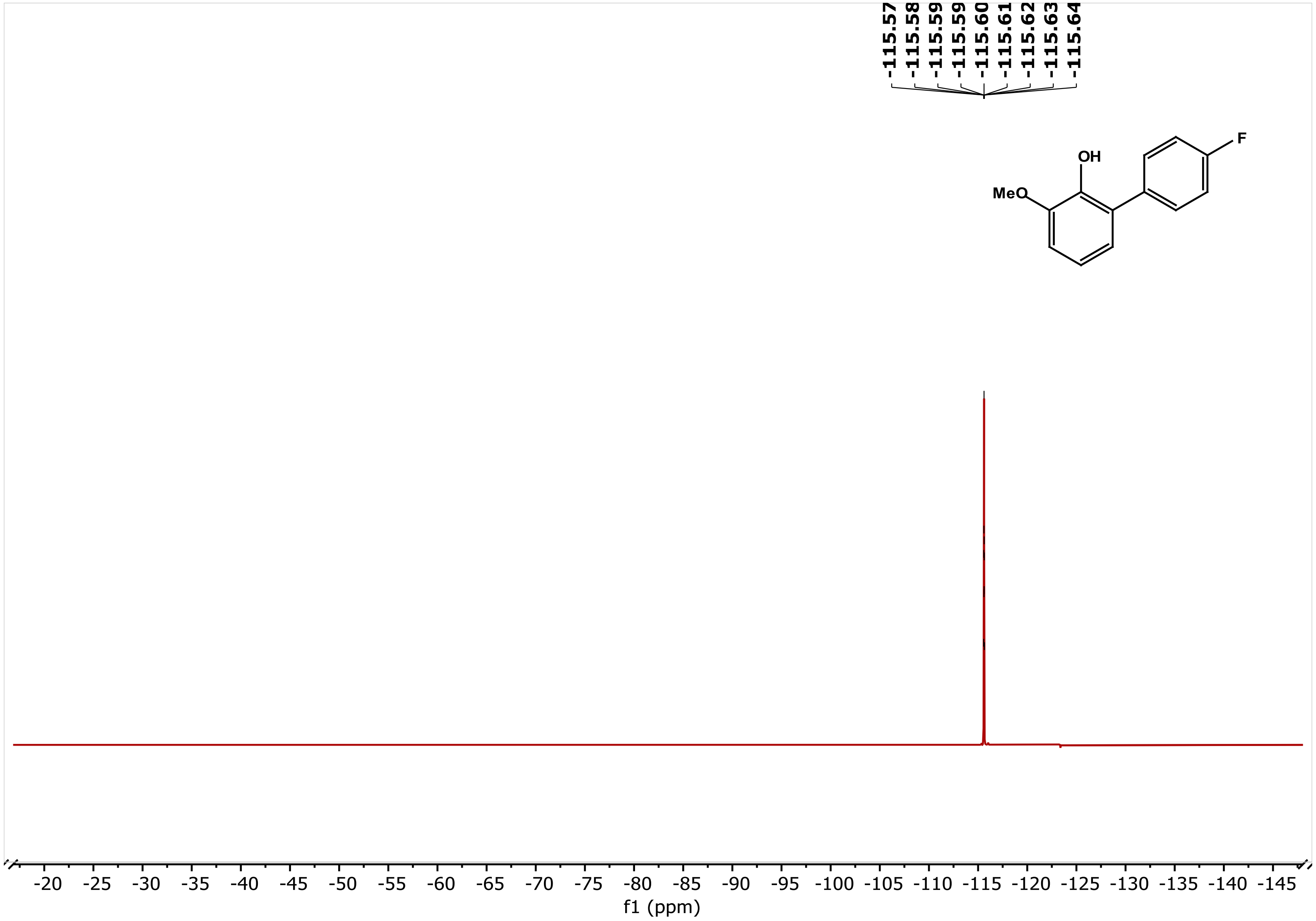

4'-Fluoro-3-methyl-[1,1'-biphenyl]-2-ol (17)

^{1}H NMR (400 MHz, $CDCl_3$):

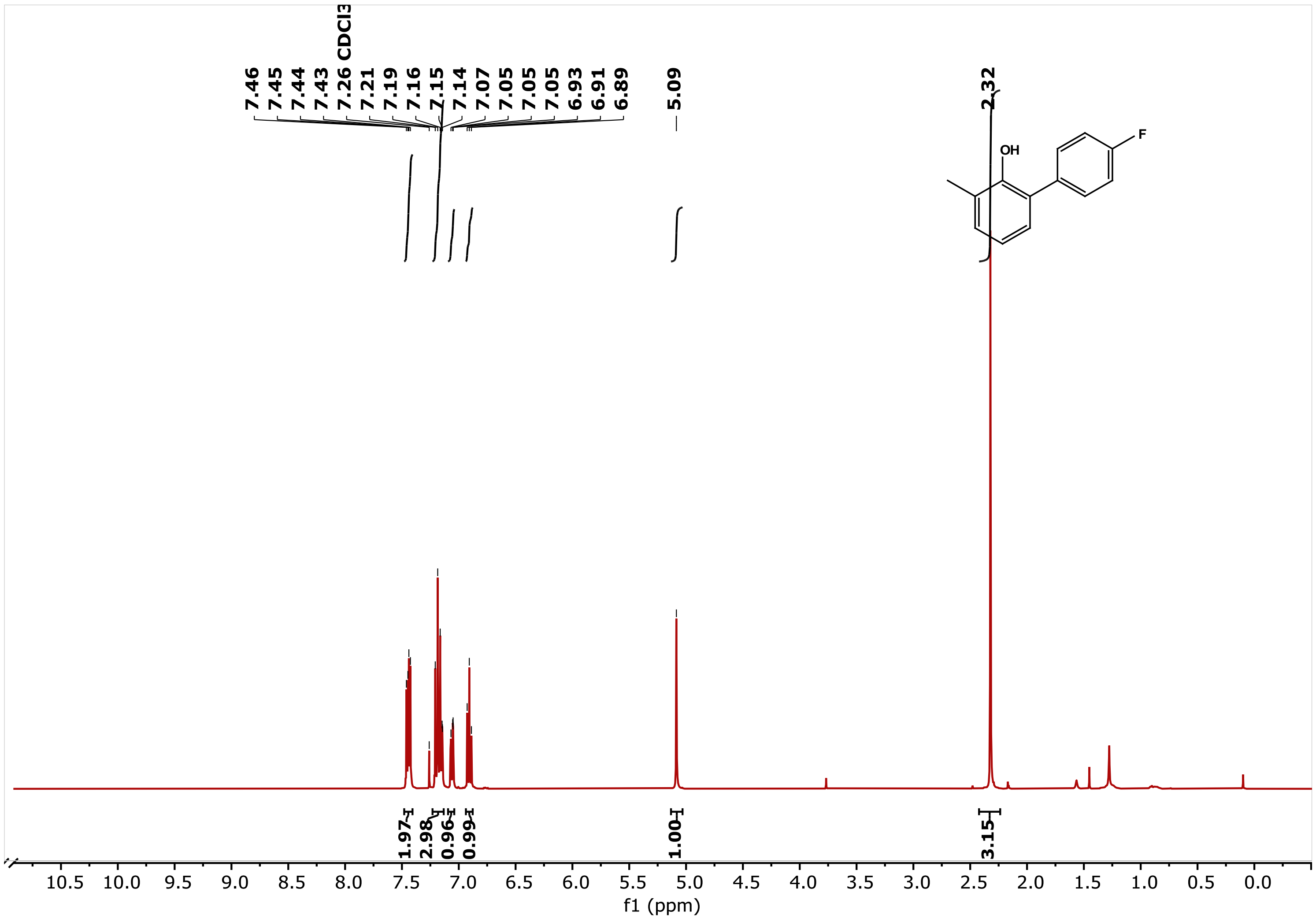

¹³C{¹H} NMR ($CDCl_3$, 101 MHz):

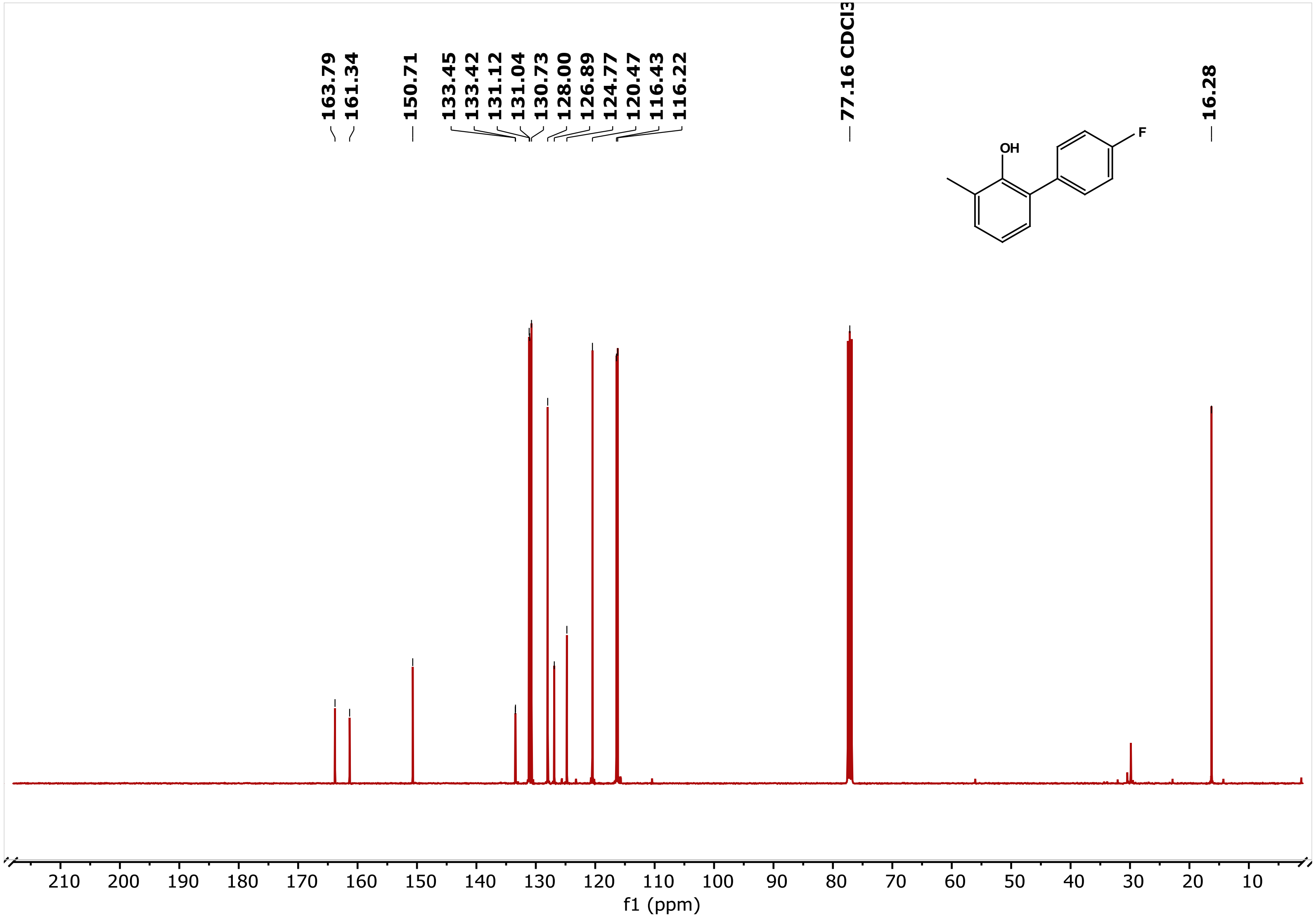

^{19}F NMR (376 MHz, $CDCl_3$):

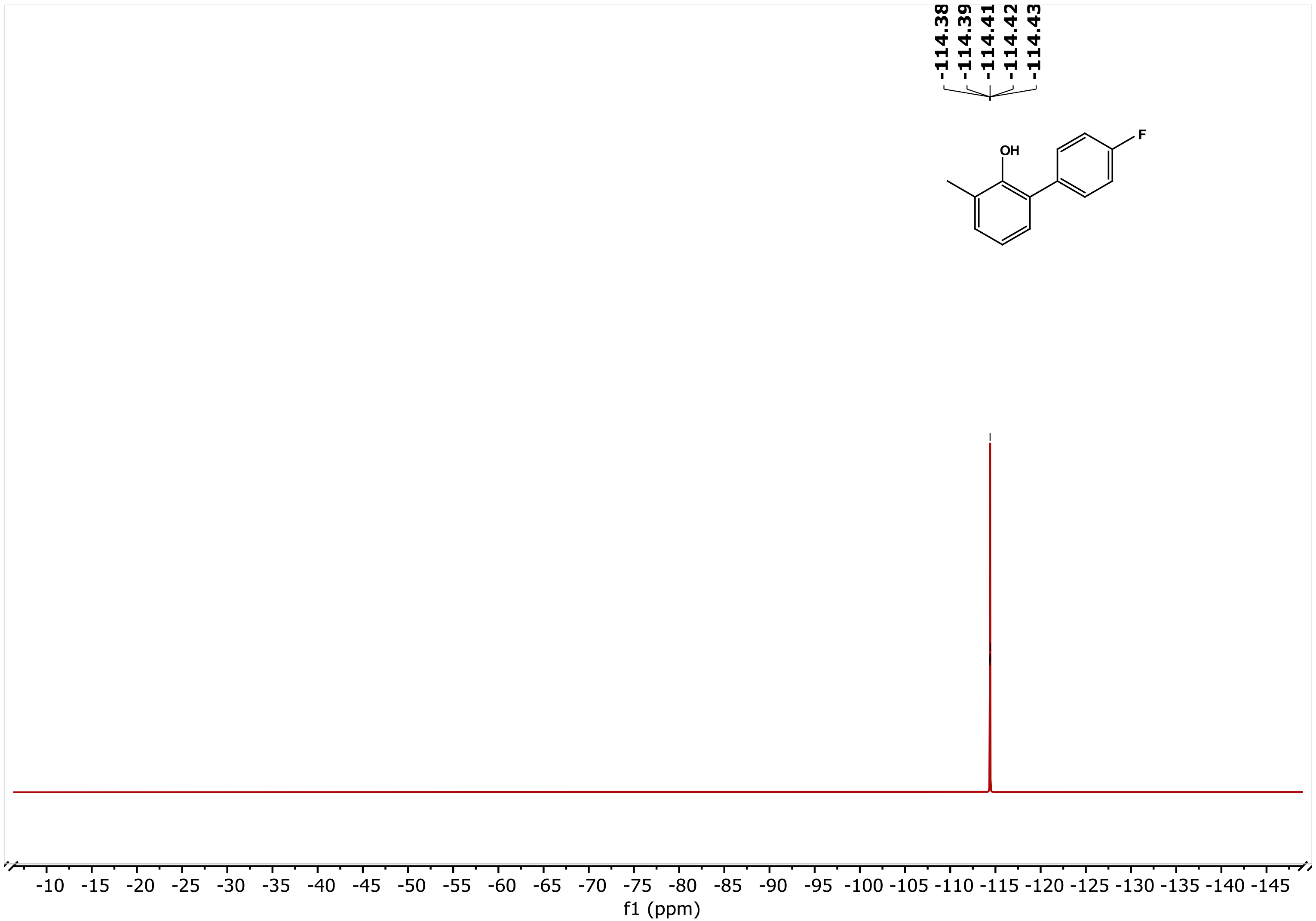

1,7-Dibromo-5,9-bis(4-fluorophenyl)-5,9-dimethoxy-1,4a,5,8a-tetrahydro-1,4-ethanonaphthalene-6,10(4H)-dione (18a)₂

^{1}H NMR (400 MHz, $CDCl_3$):

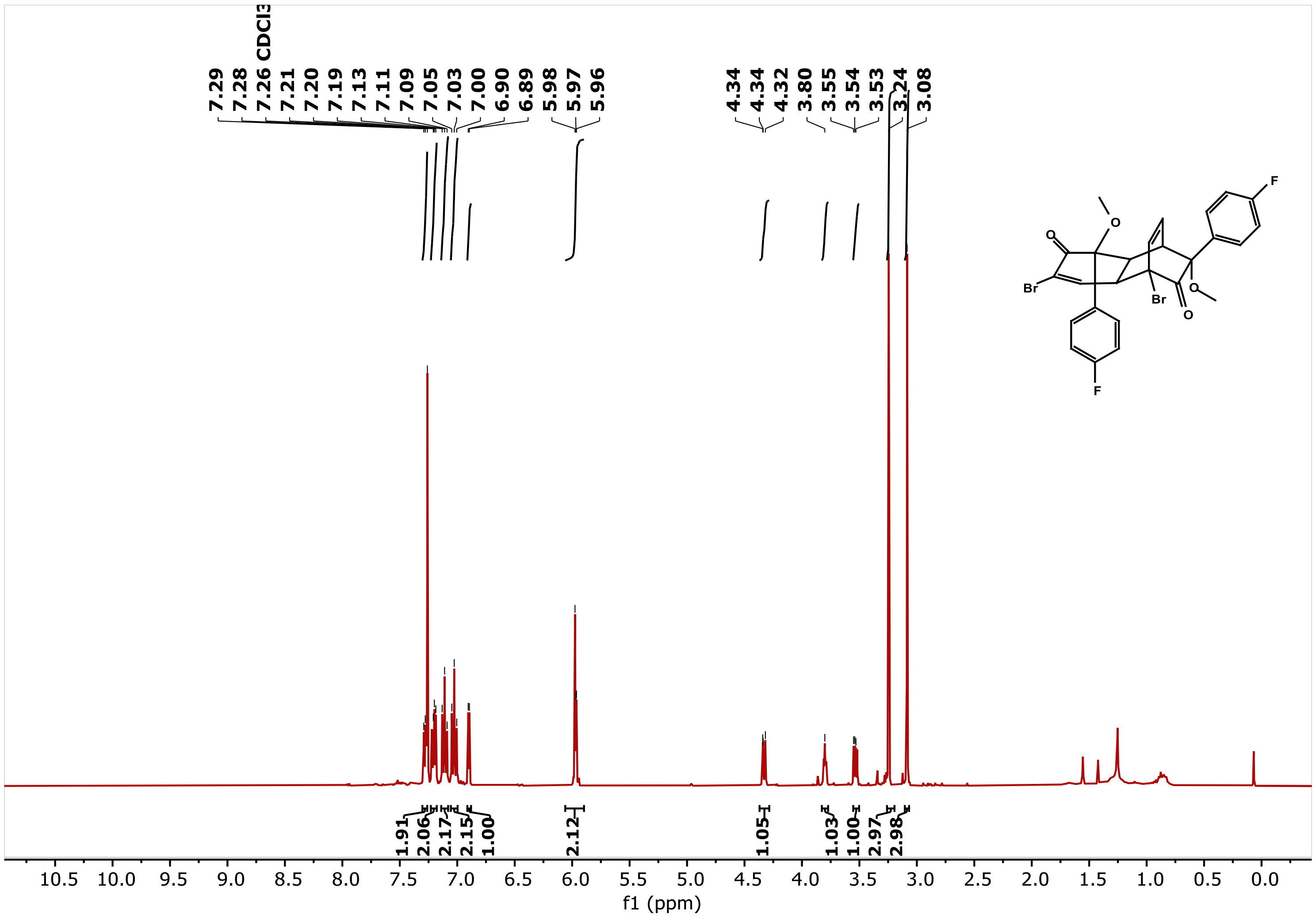

$^{13}C\{^1H\}$ NMR ($CDCl_3$, 101 MHz):

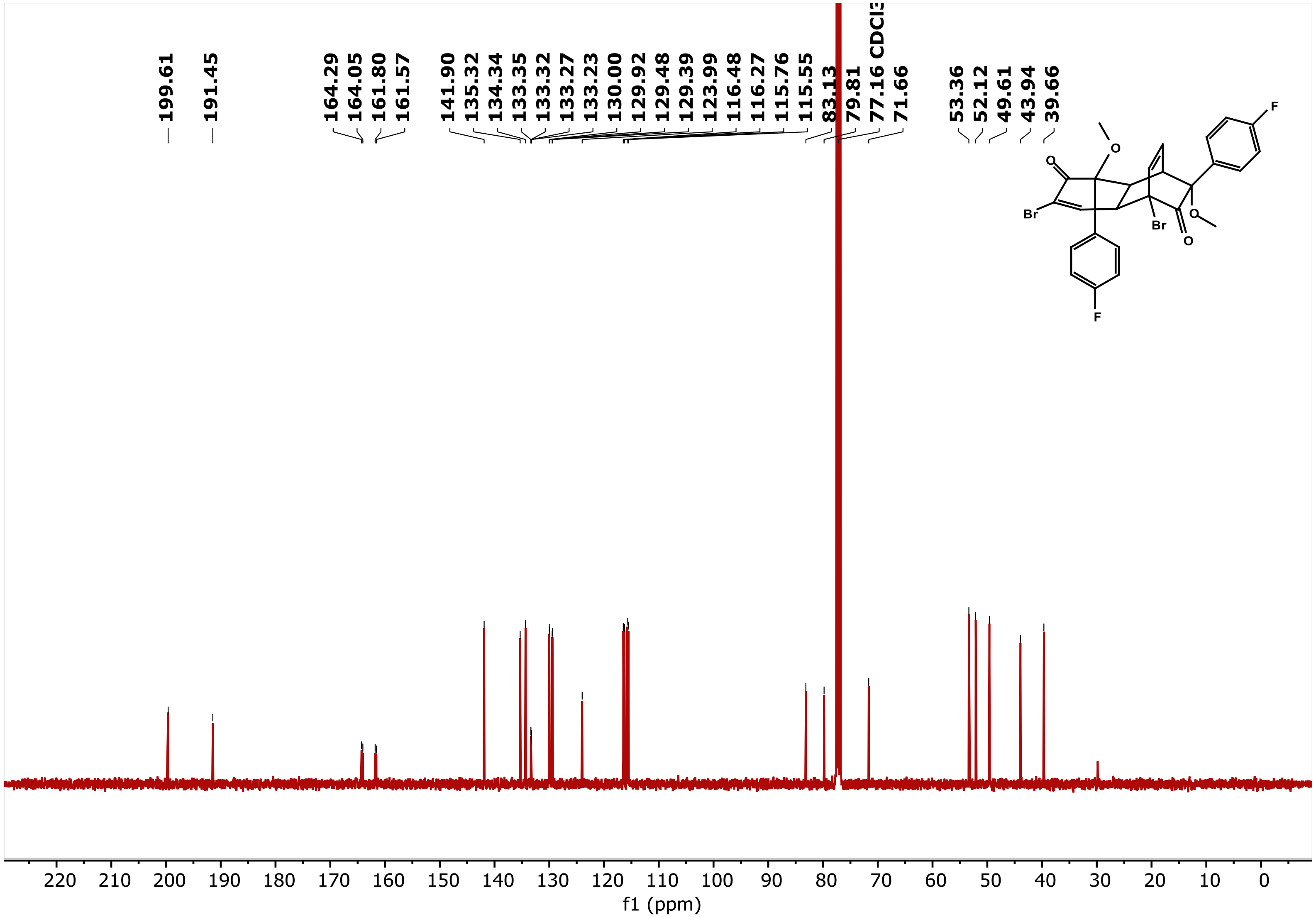

^{19}F NMR (376 MHz, $CDCl_3$):

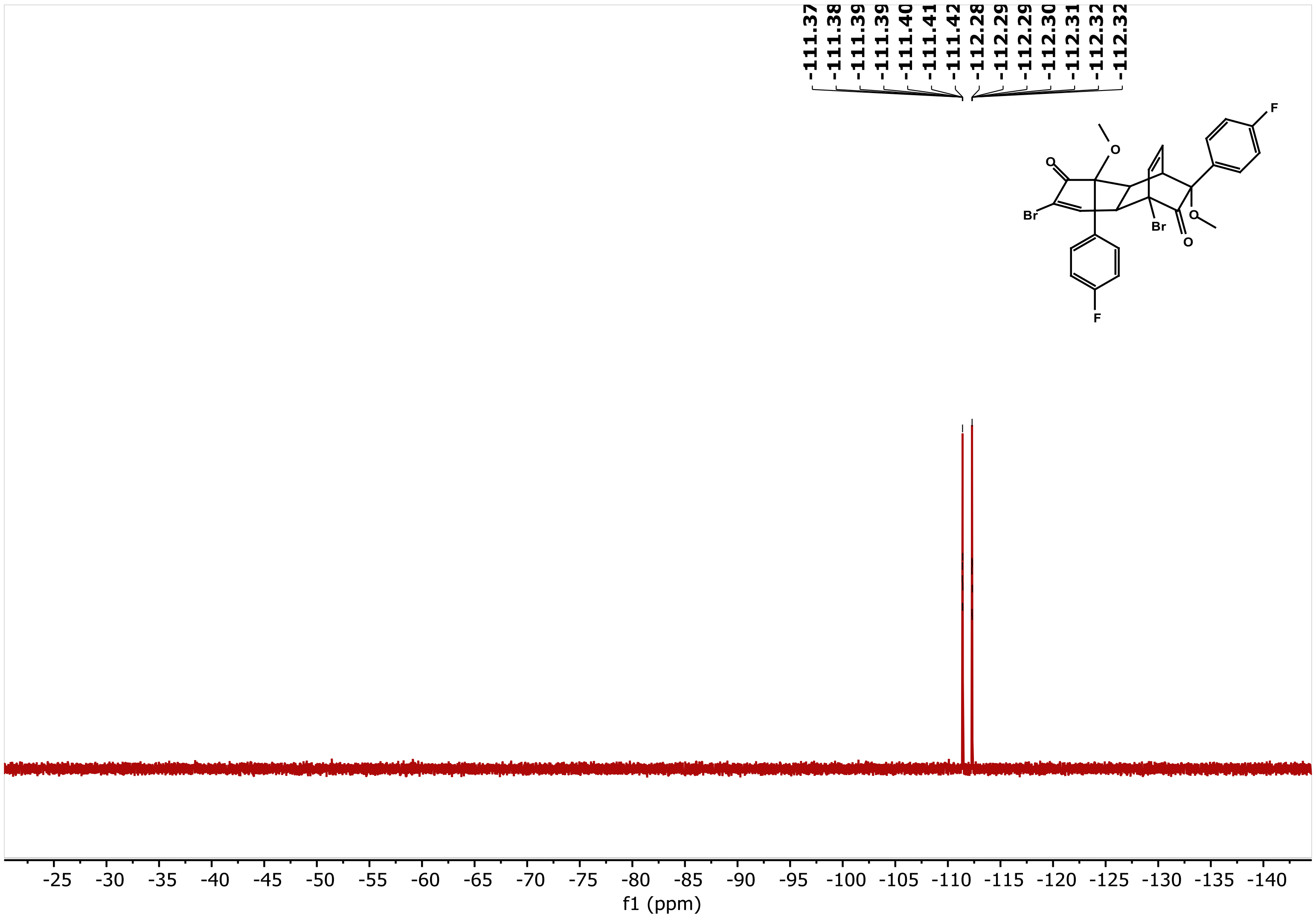

4'-Fluoro-4-methyl-[1,1'-biphenyl]-2-ol (19)

¹H NMR (400 MHz, $CDCl_3$):

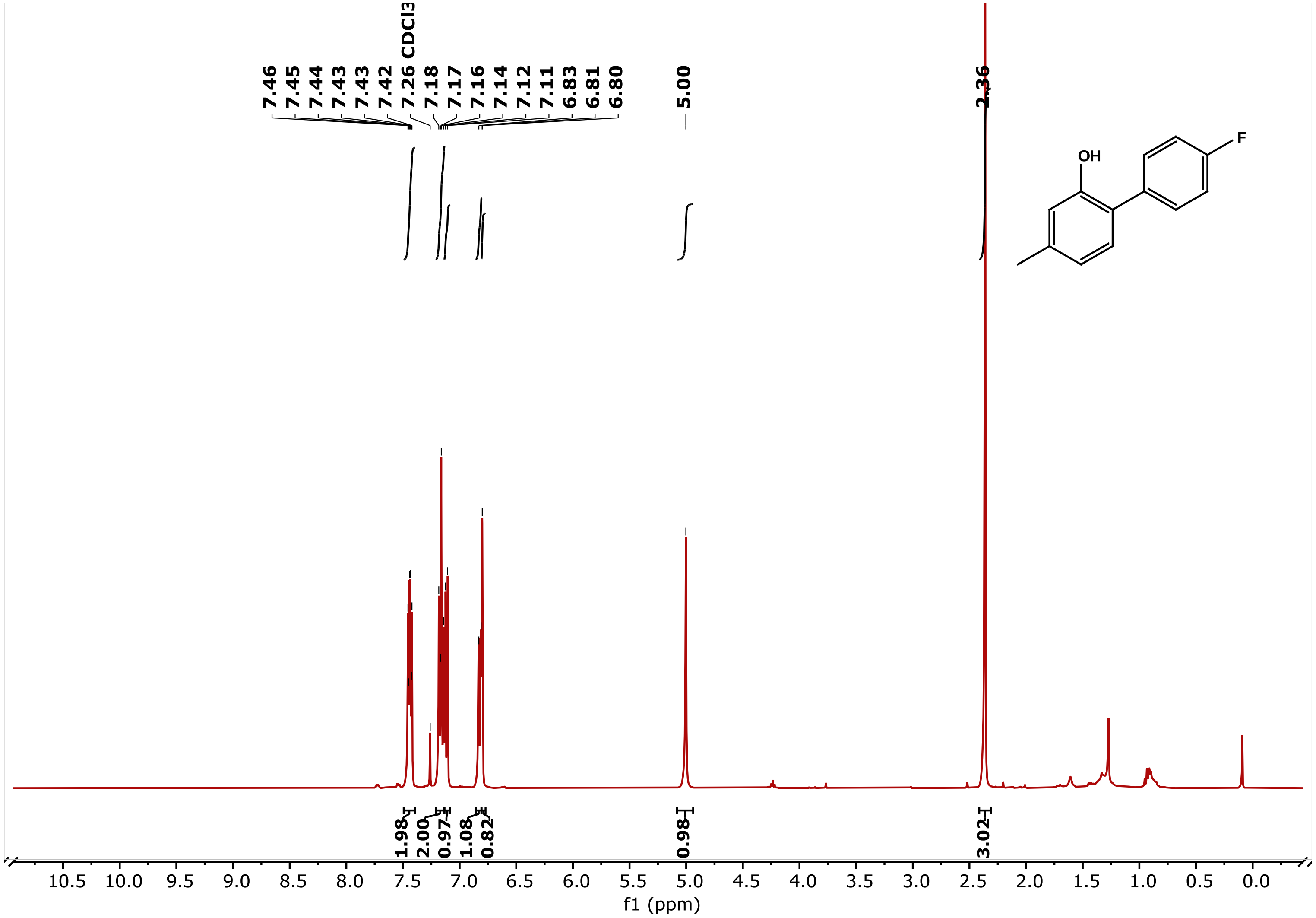

$^{13}C\{^1H\}$ **NMR** (126 MHz, $CDCl_3$):

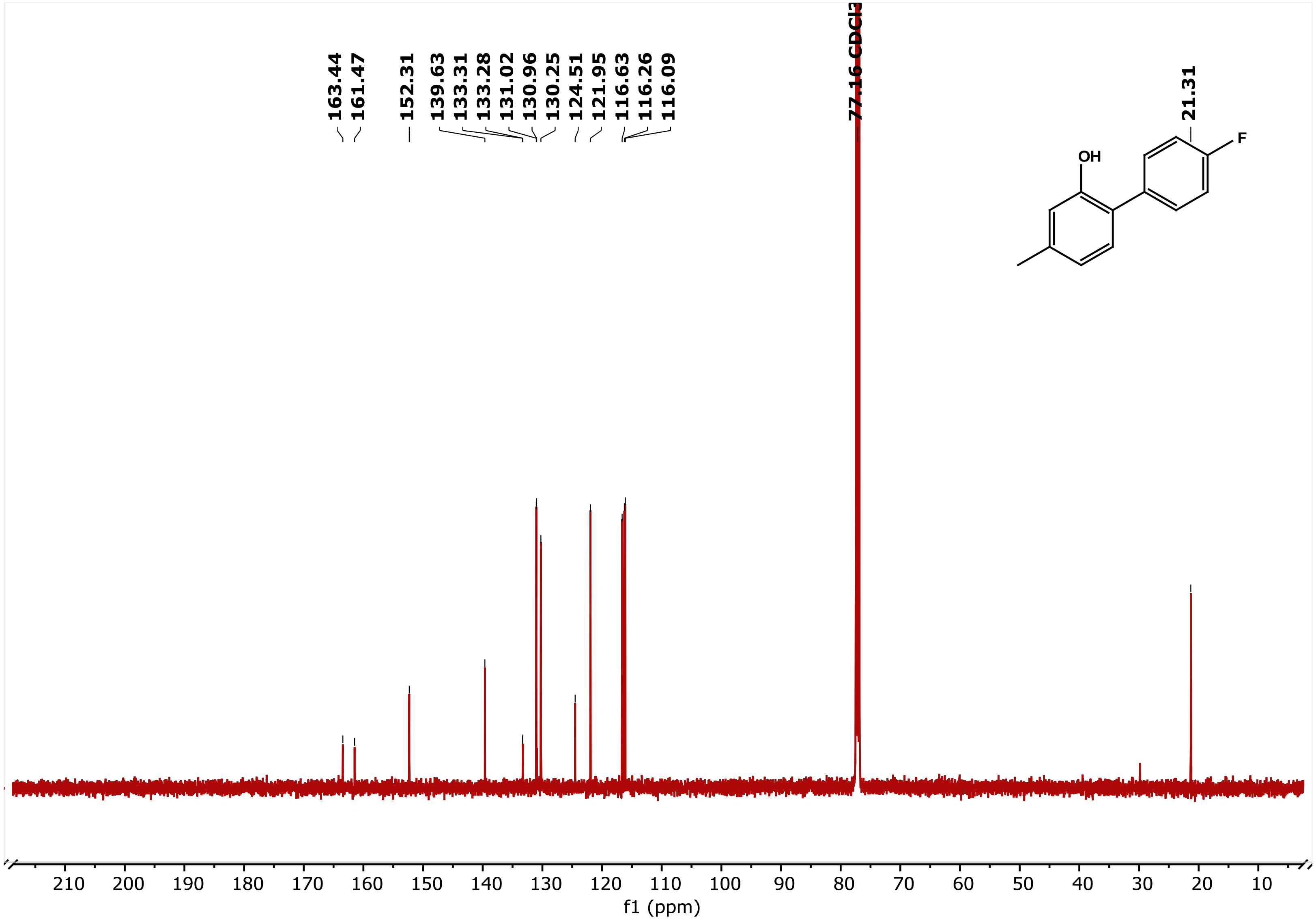

¹⁹F NMR (377 MHz, $CDCl_3$):

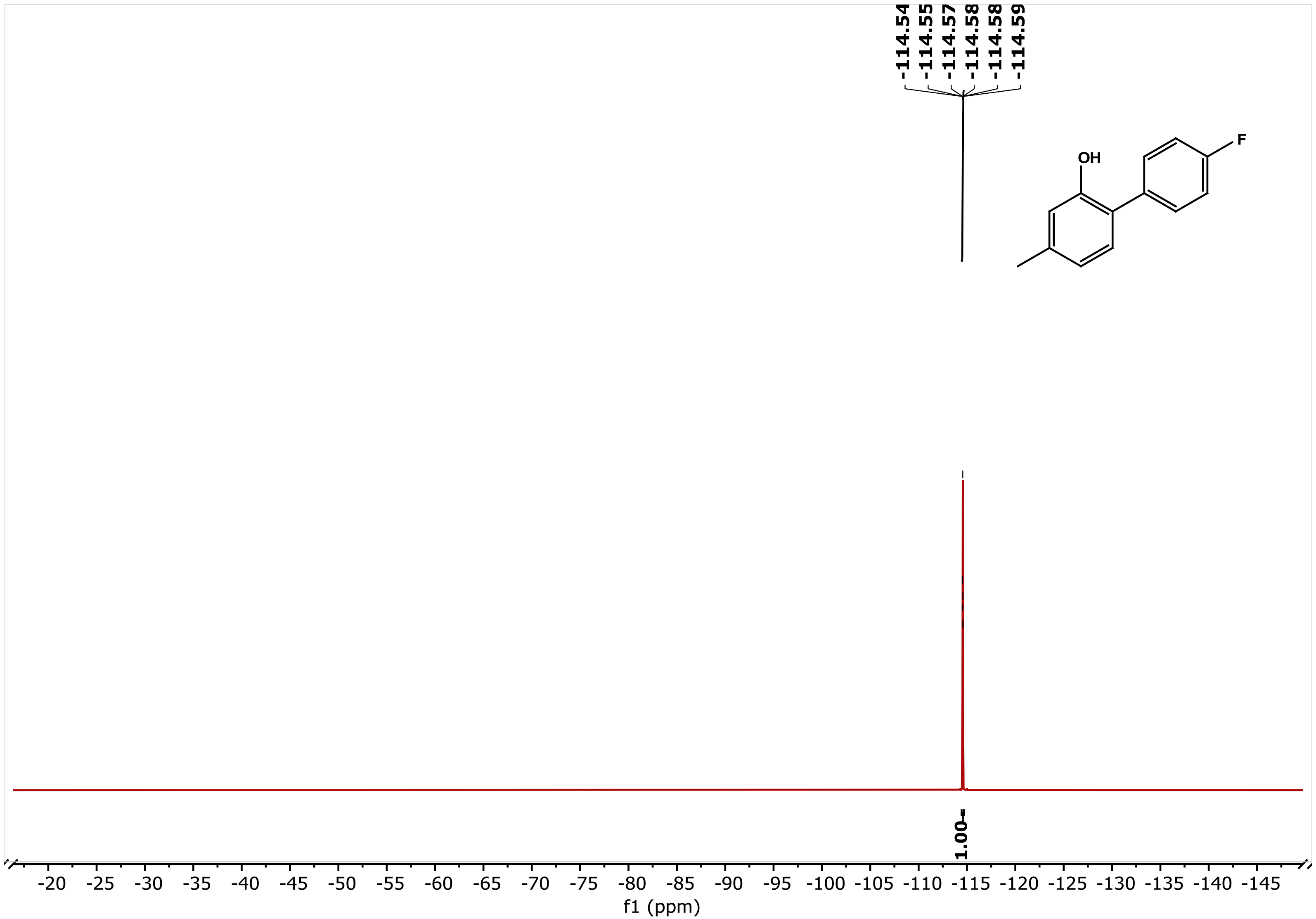

4-Bromo-4'-fluoro-[1,1'-biphenyl]-2-ol (20)

^{1}H NMR (500 MHz, $CDCl_3$):

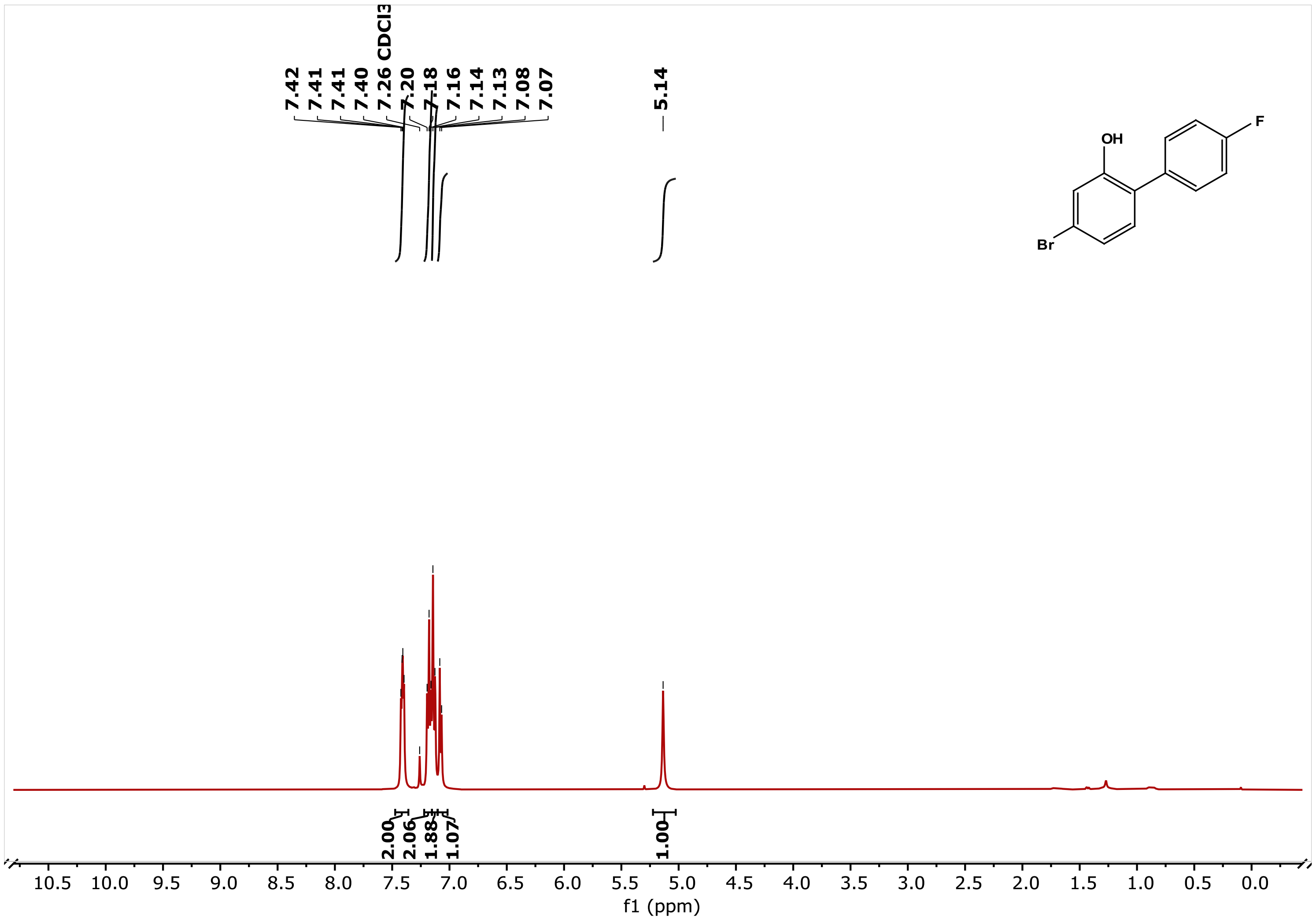

$^{13}C\{^{1}H\}$ **NMR** ($CDCl_3$, 126 MHz)

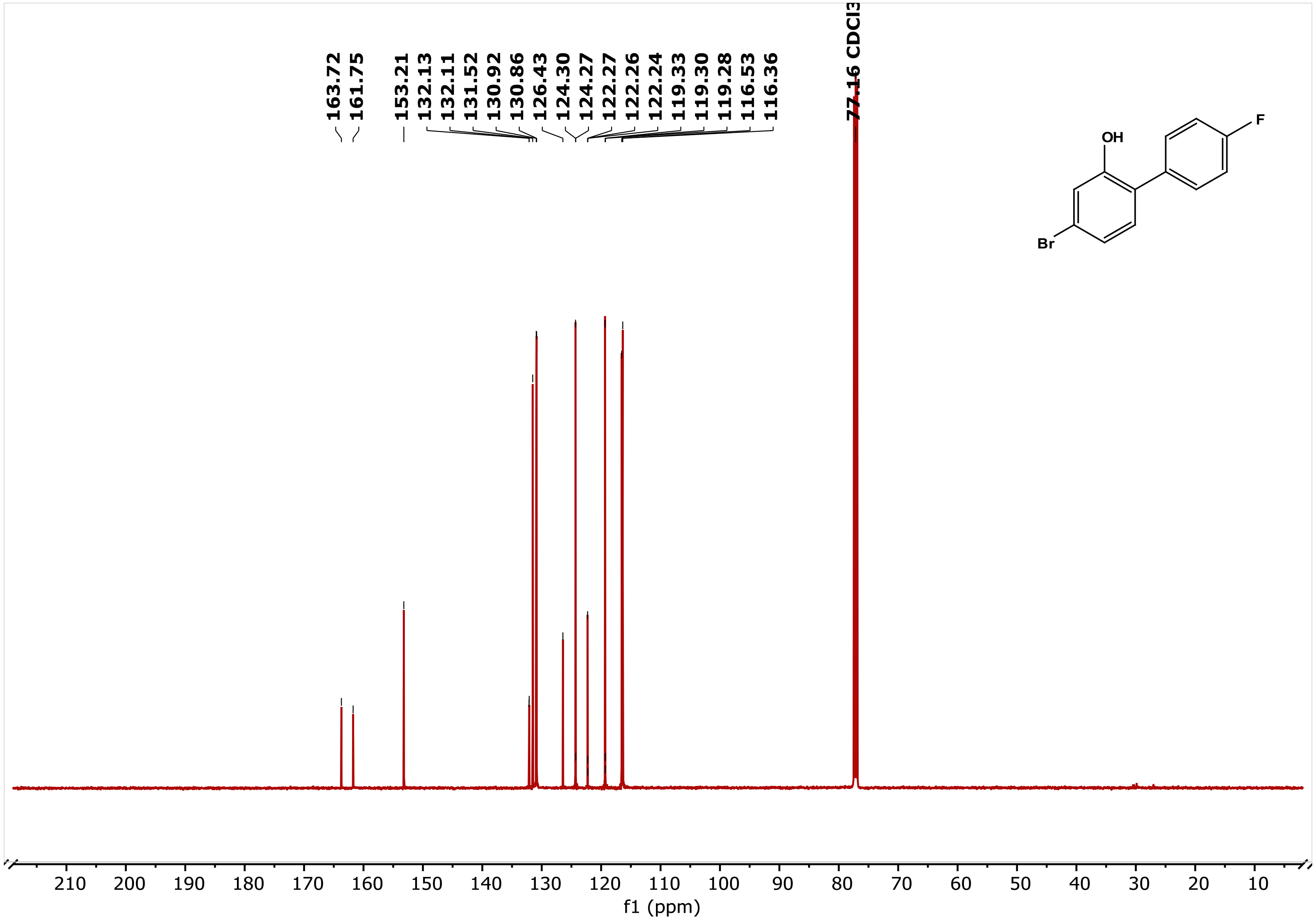

^{19}F NMR (377 MHz, $CDCl_3$):

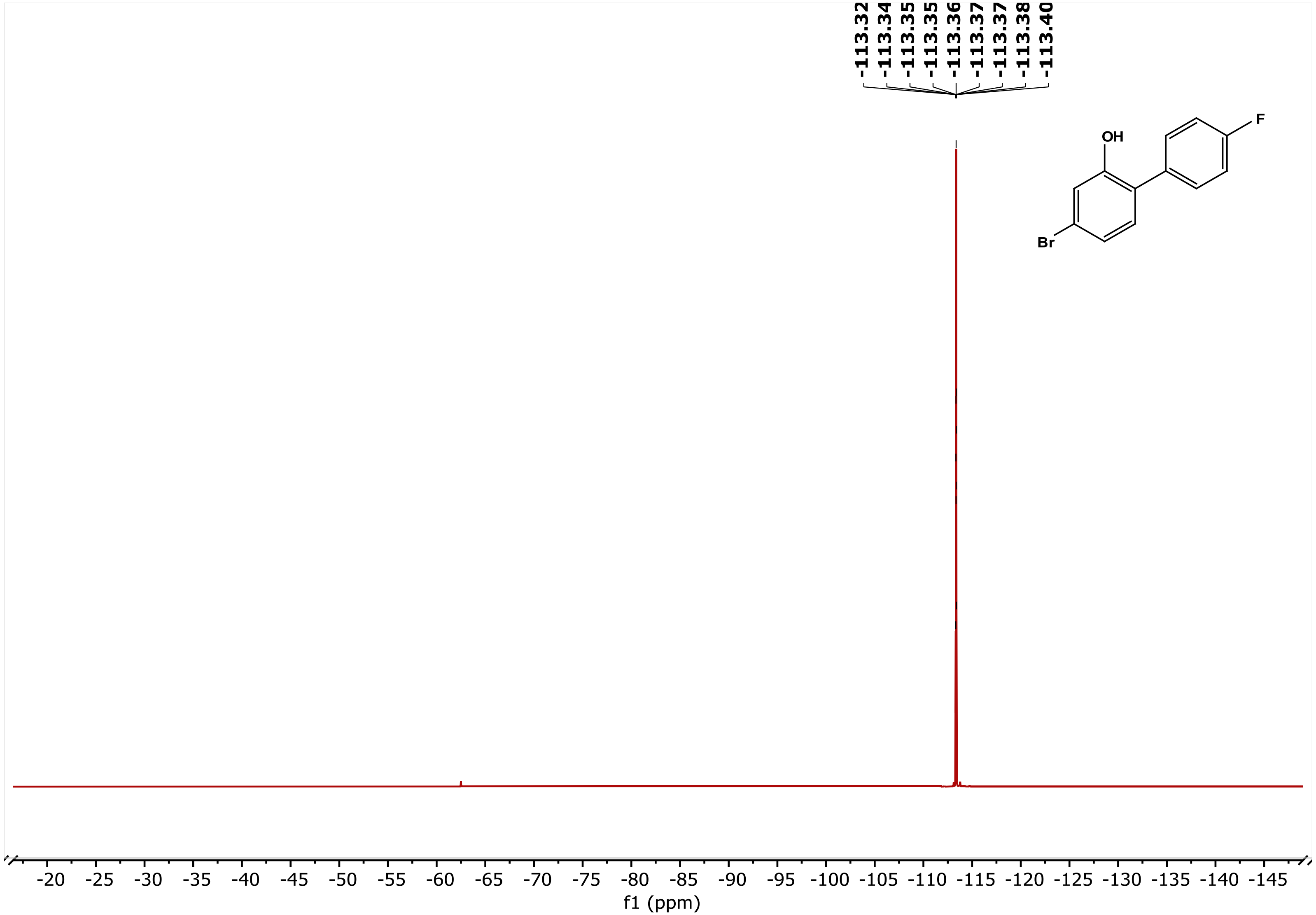

4'-Fluoro-5-methyl-[1,1'-biphenyl]-2-ol (21)

^{1}H NMR (400 MHz, $CDCl_3$):

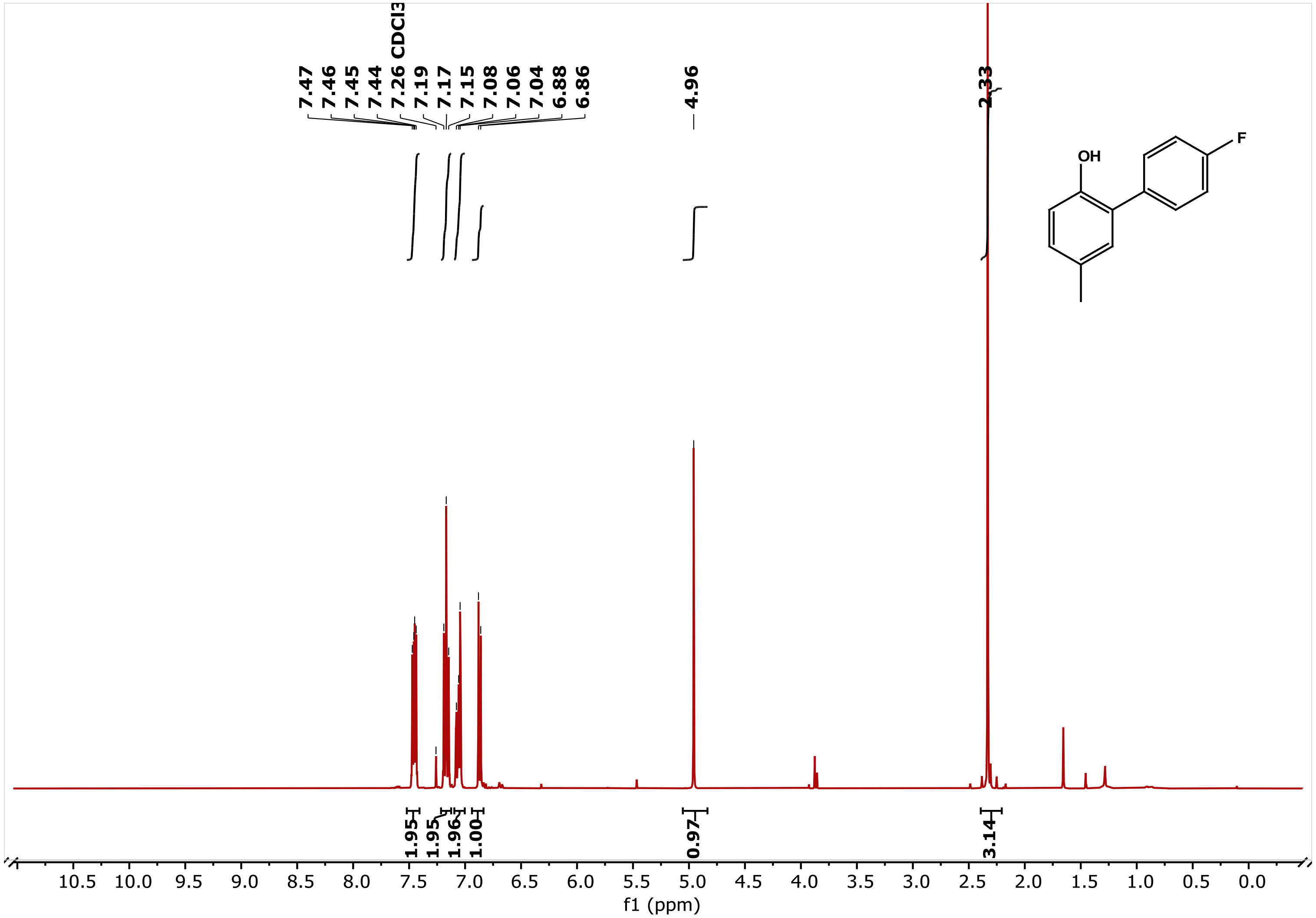

^{13}C{^{1}H} NMR (101 MHz, $CDCl_3$):

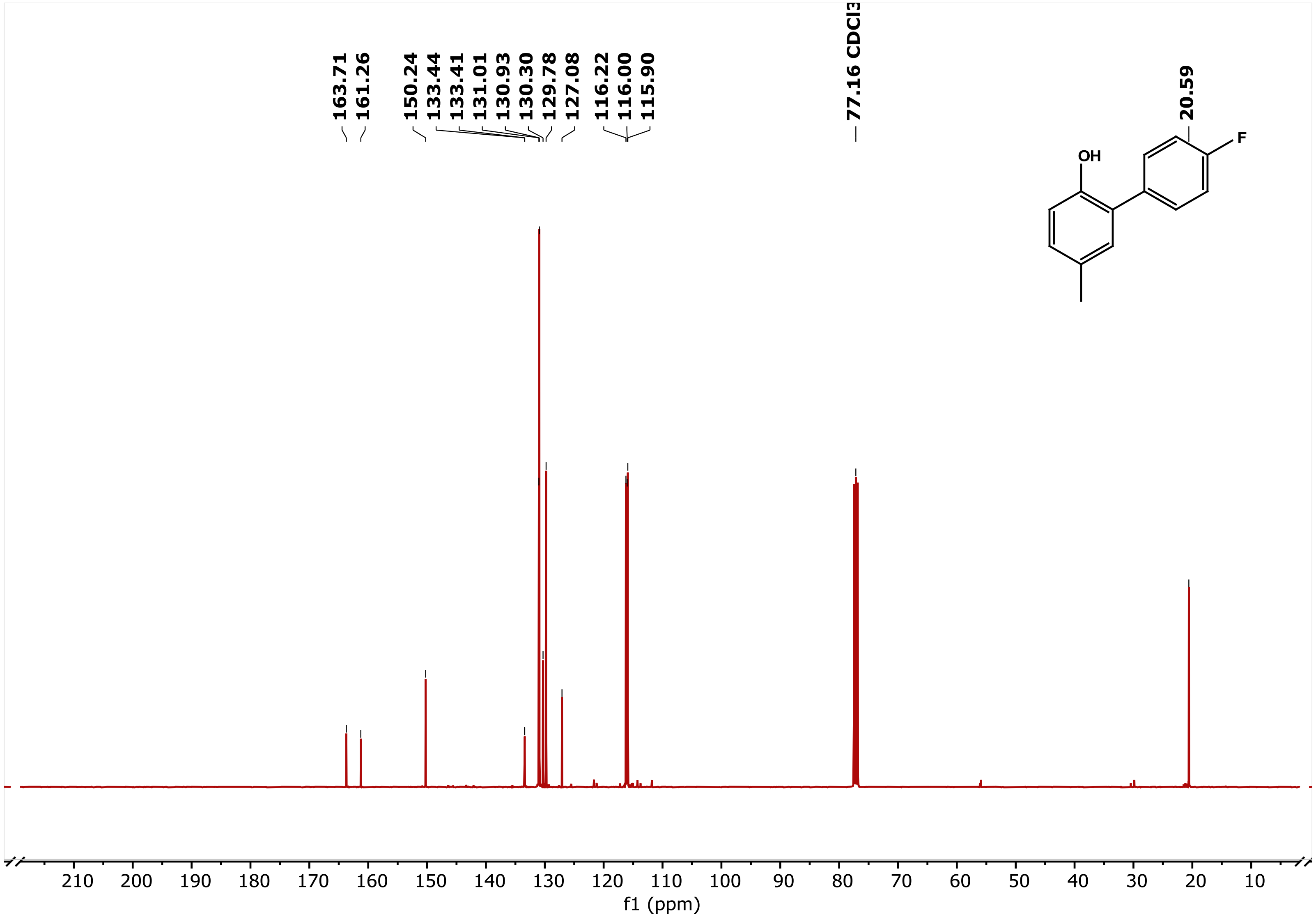

^{19}F NMR (377 MHz, $CDCl_3$):

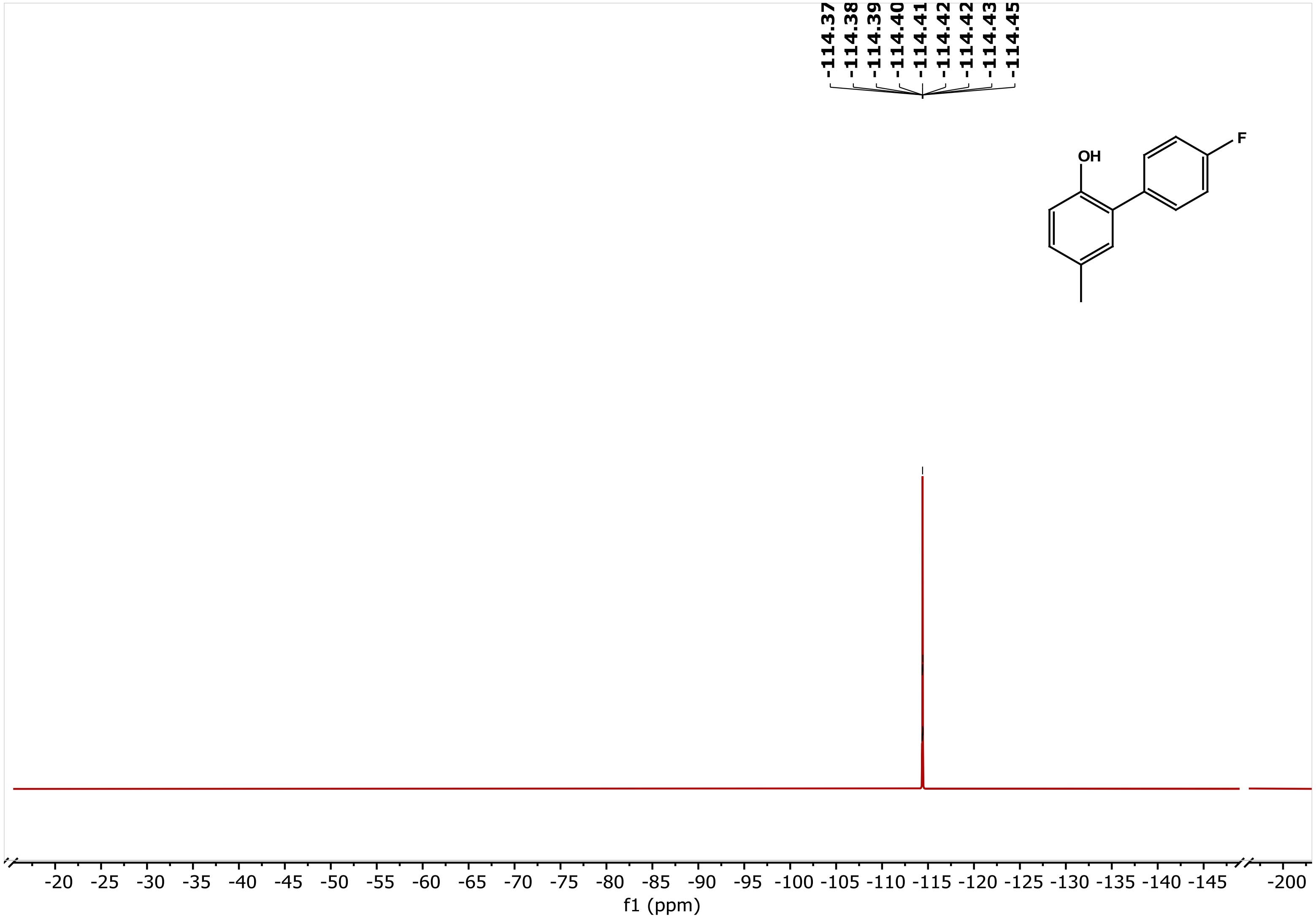

5-Bromo-4'-fluoro-[1,1'-biphenyl]-2-ol (22)

^{1}H NMR (400 MHz, $CDCl_3$):

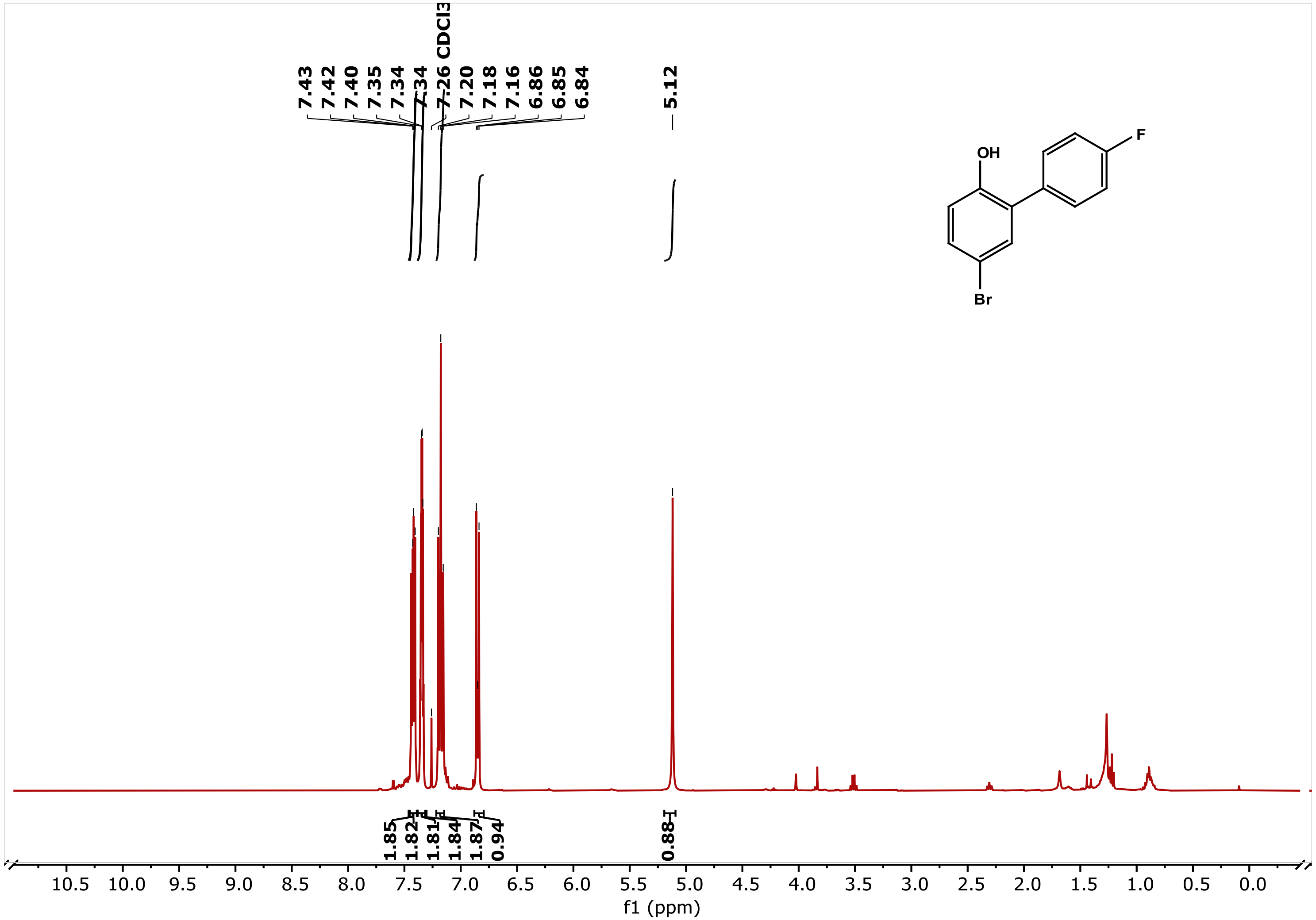

^{13}C{^{1}H} NMR (CDCl$_3$, 101 MHz)

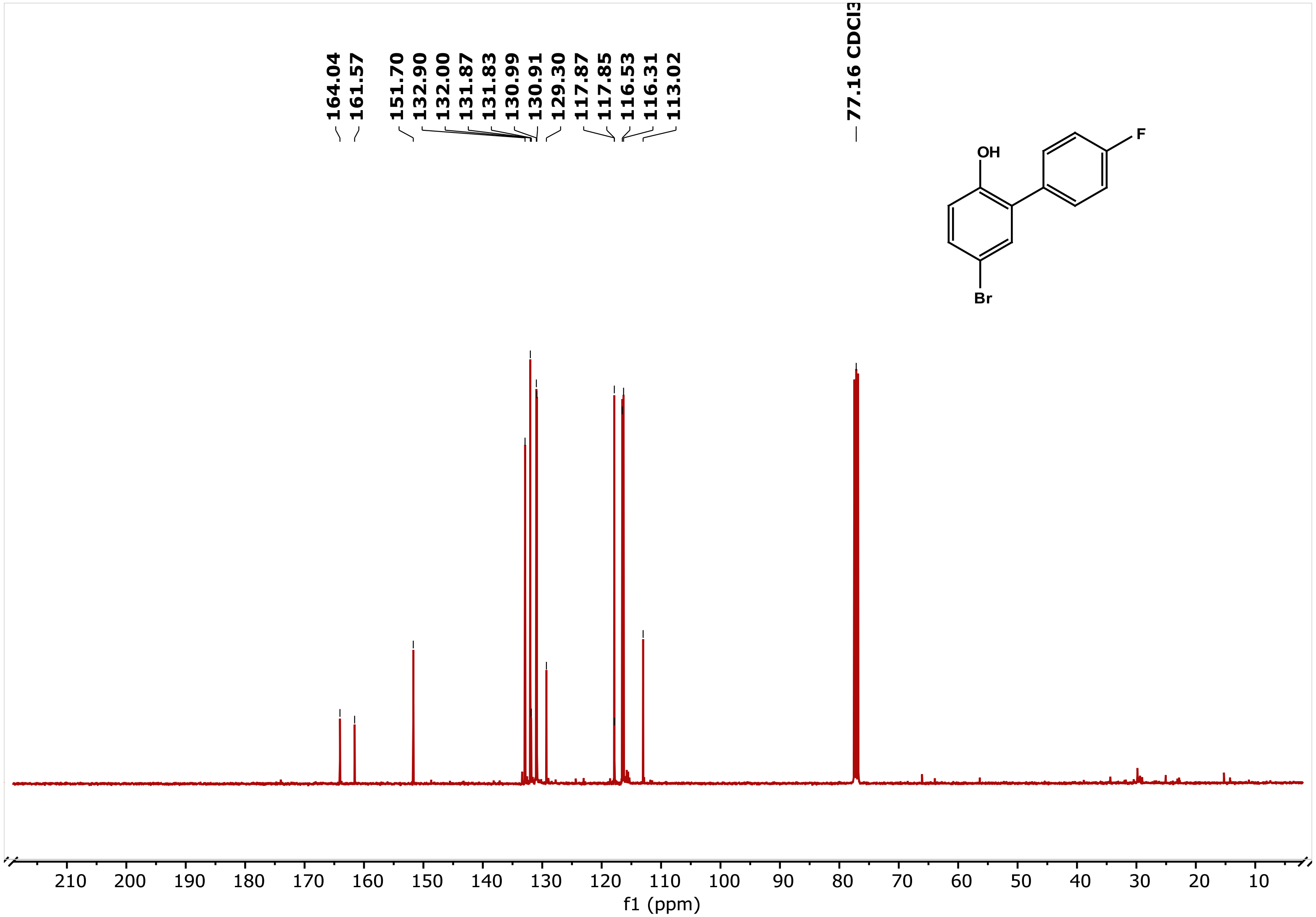

^{19}F NMR (376 MHz, $CDCl_3$):

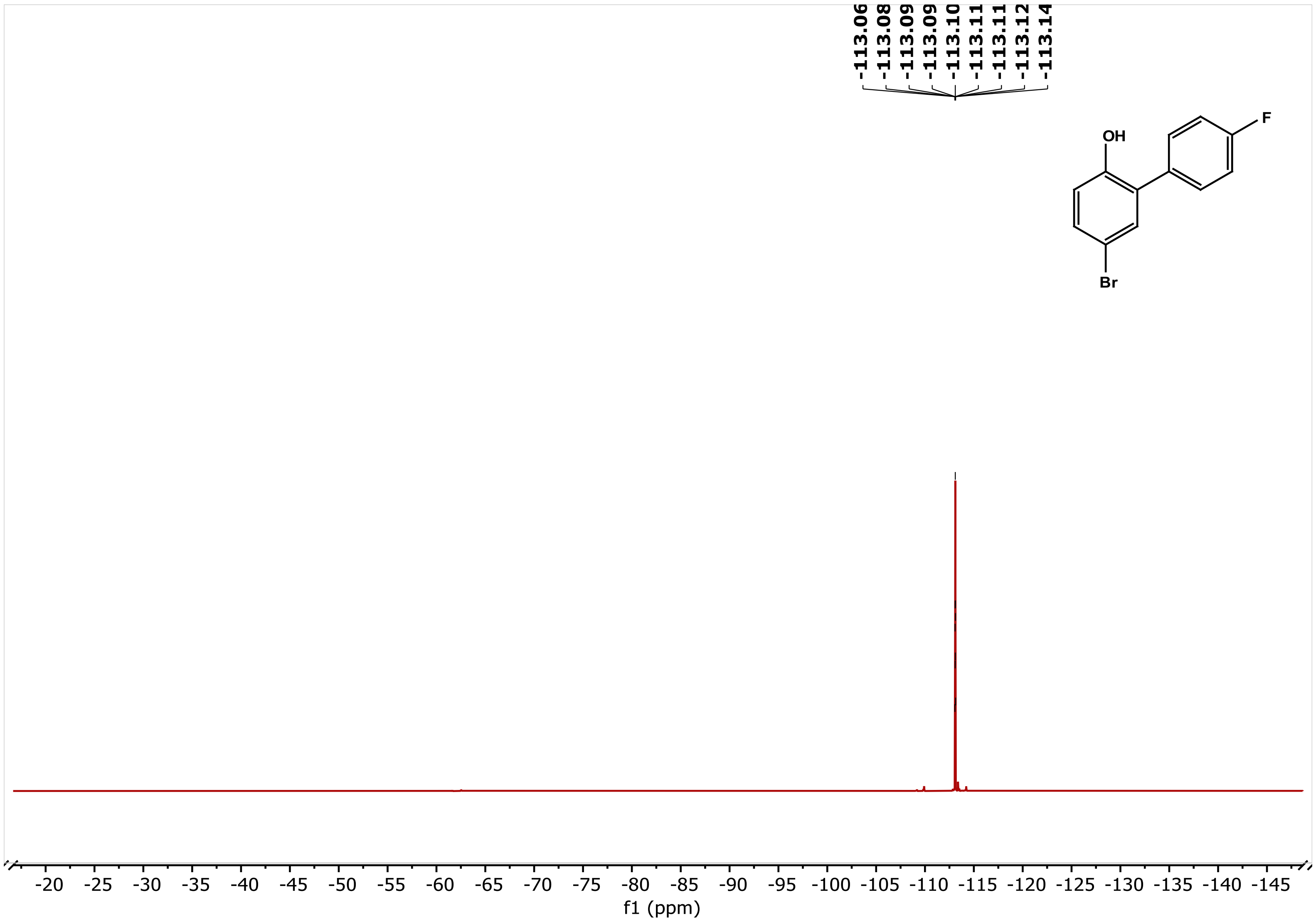

Methyl (*E*)-3-(4'-fluoro-6-hydroxy-[1,1'-biphenyl]-3-yl)acrylate (23)

^{1}H NMR (400 MHz, $CDCl_3$):

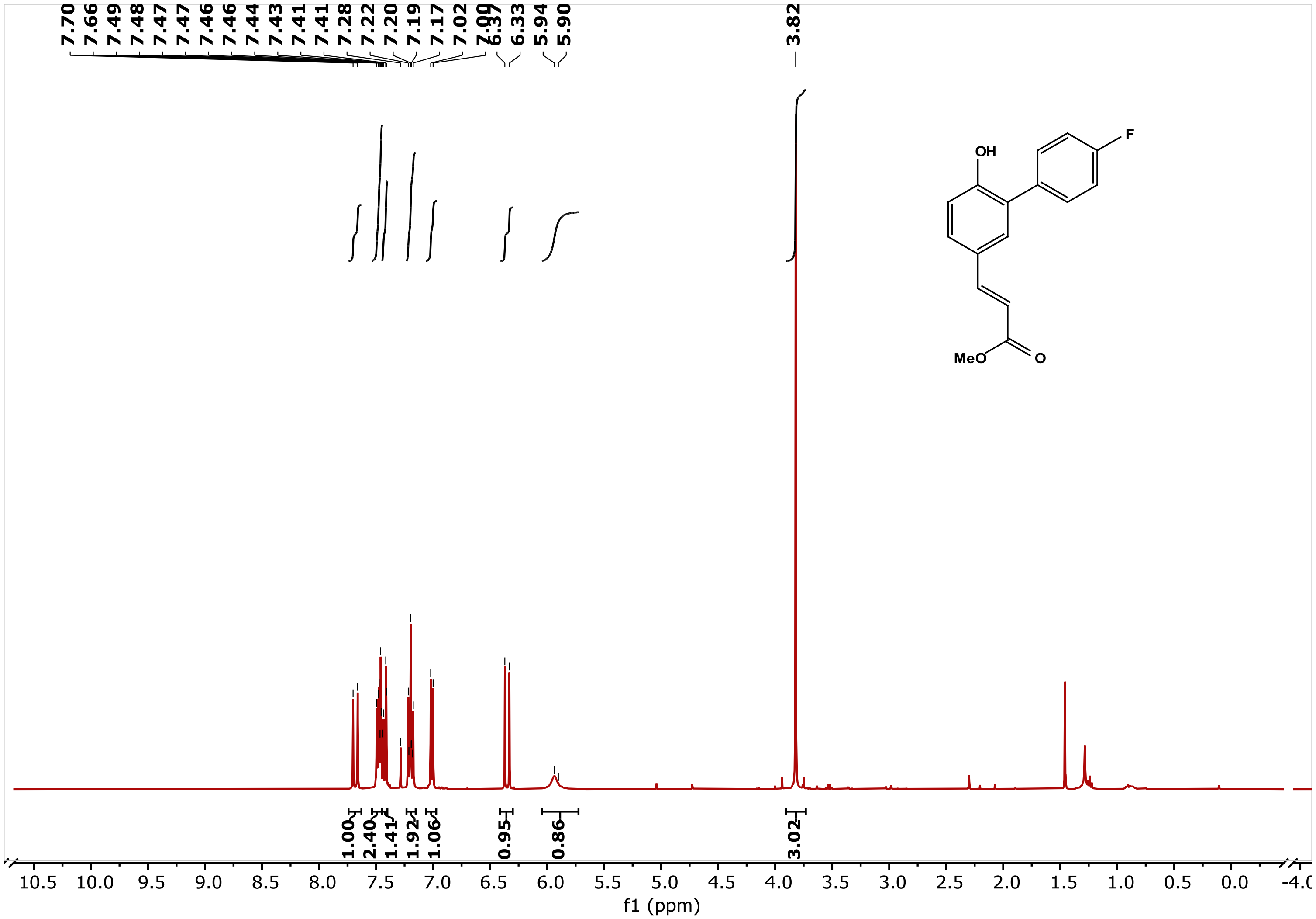

^{13}C{^{1}H} NMR ($CDCl_3$, 101 MHz)

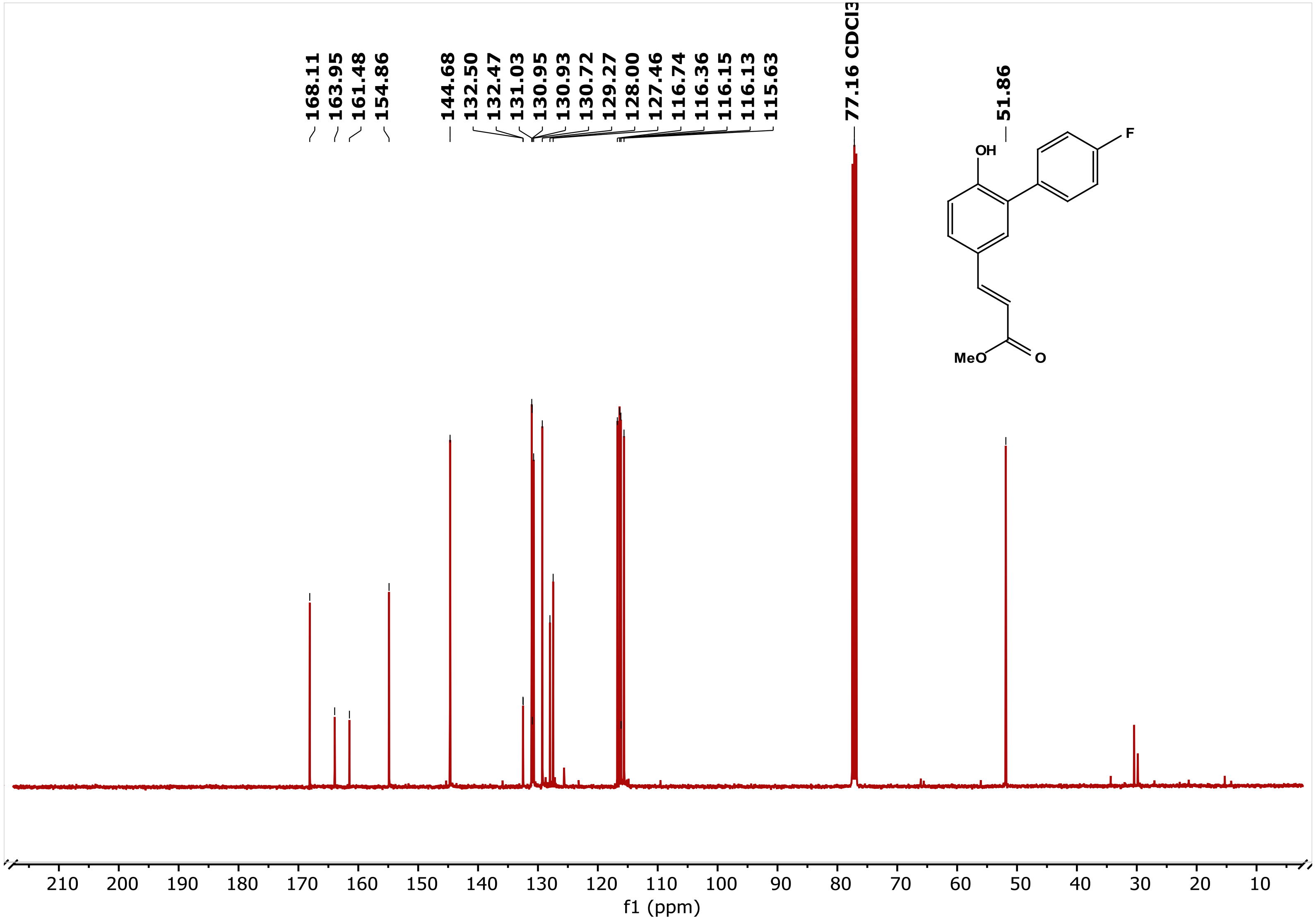

^{19}F NMR (376 MHz, $CDCl_3$):

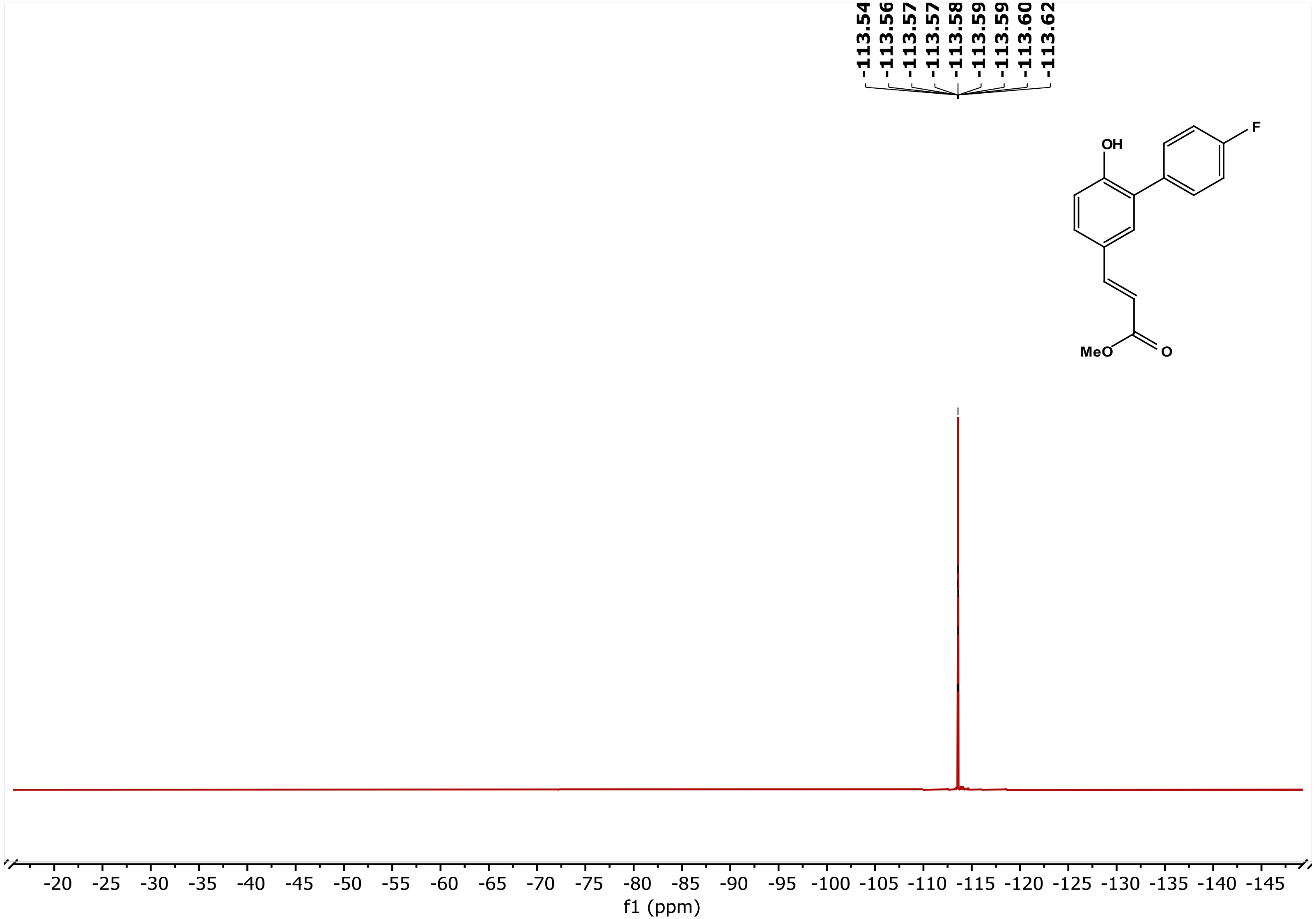

4'-Fluoro-6-methoxy-[1,1'-biphenyl]-2-ol (24)

¹H NMR (400 MHz, $CDCl_3$):

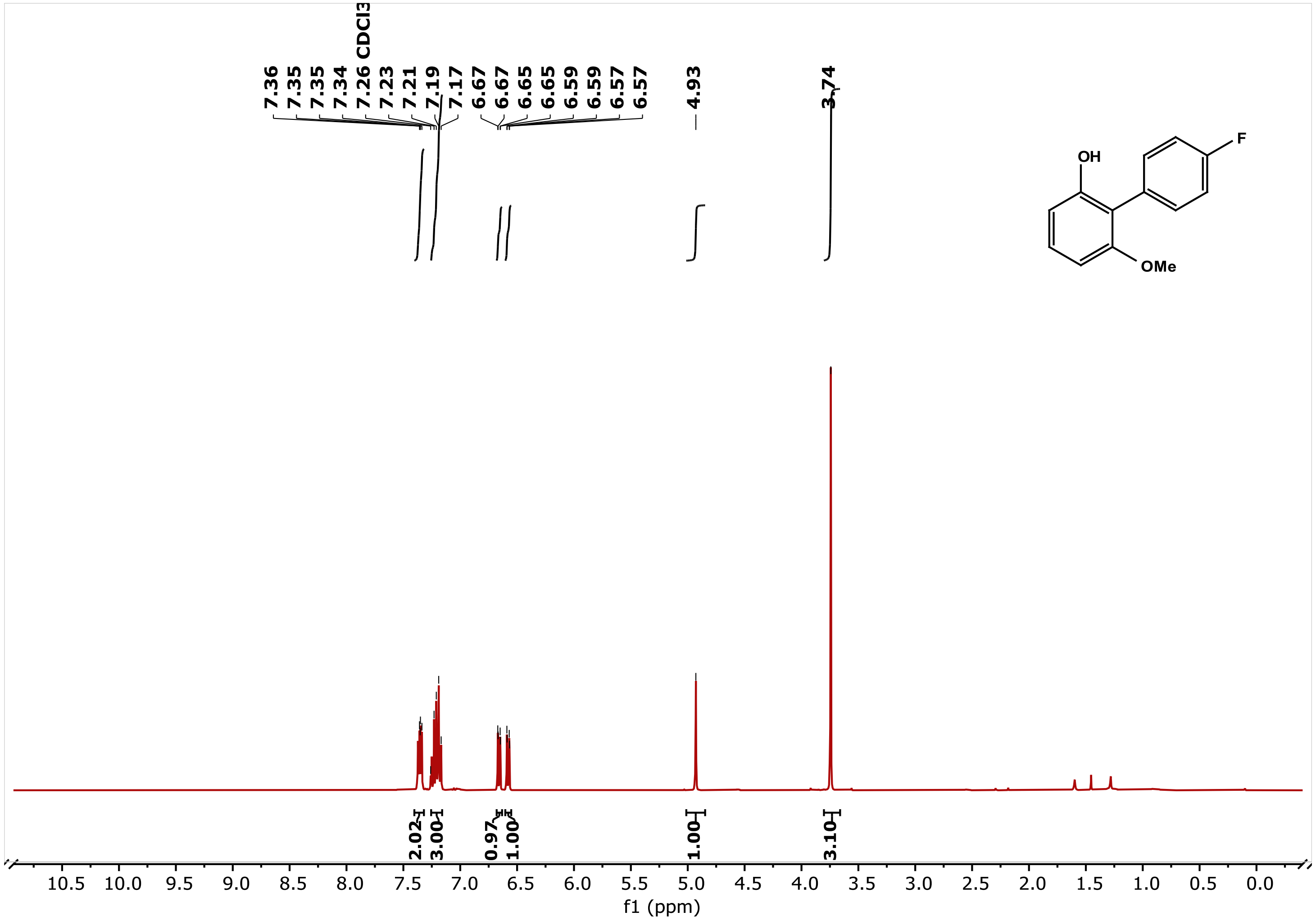

$^{13}C\{^1H\}$ NMR (101 MHz, $CDCl_3$):

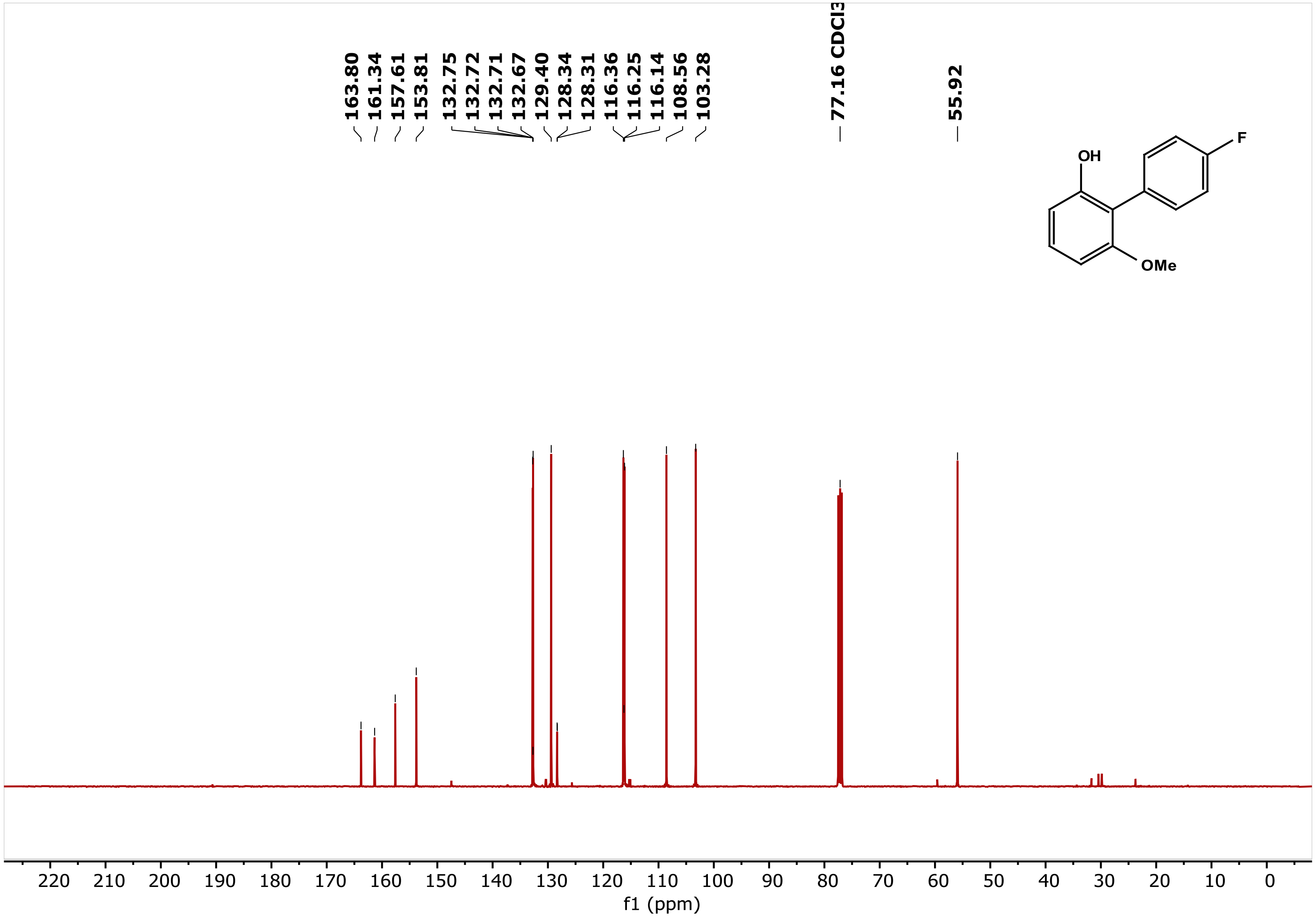

^{19}F NMR (377 MHz, $CDCl_3$):

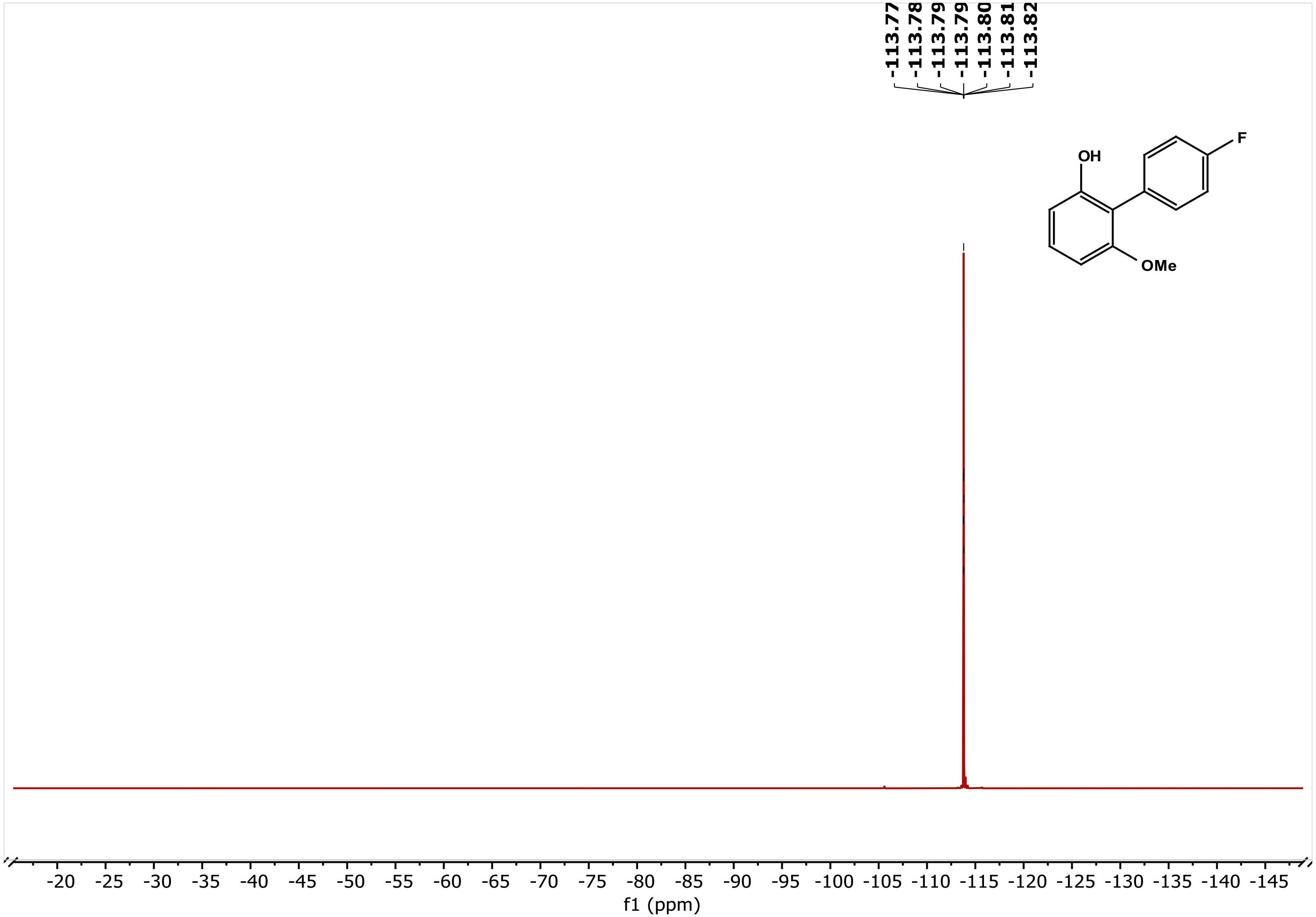

4',6-Difluoro-[1,1'-biphenyl]-2-ol (25)

^{1}H NMR (400 MHz, $CDCl_3$):

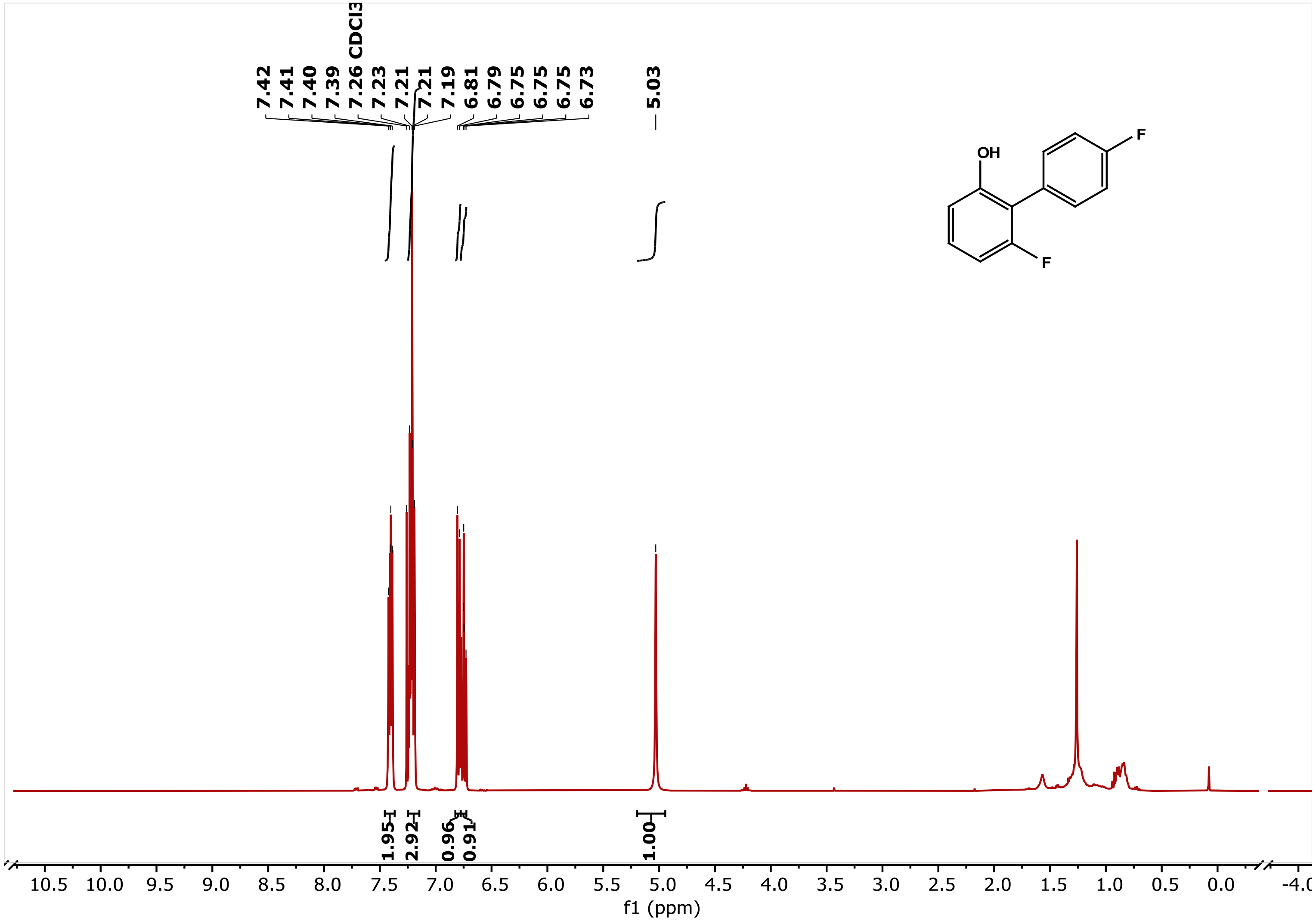

^{13}C{^{1}H} NMR (CDCl$_3$, 101 MHz)

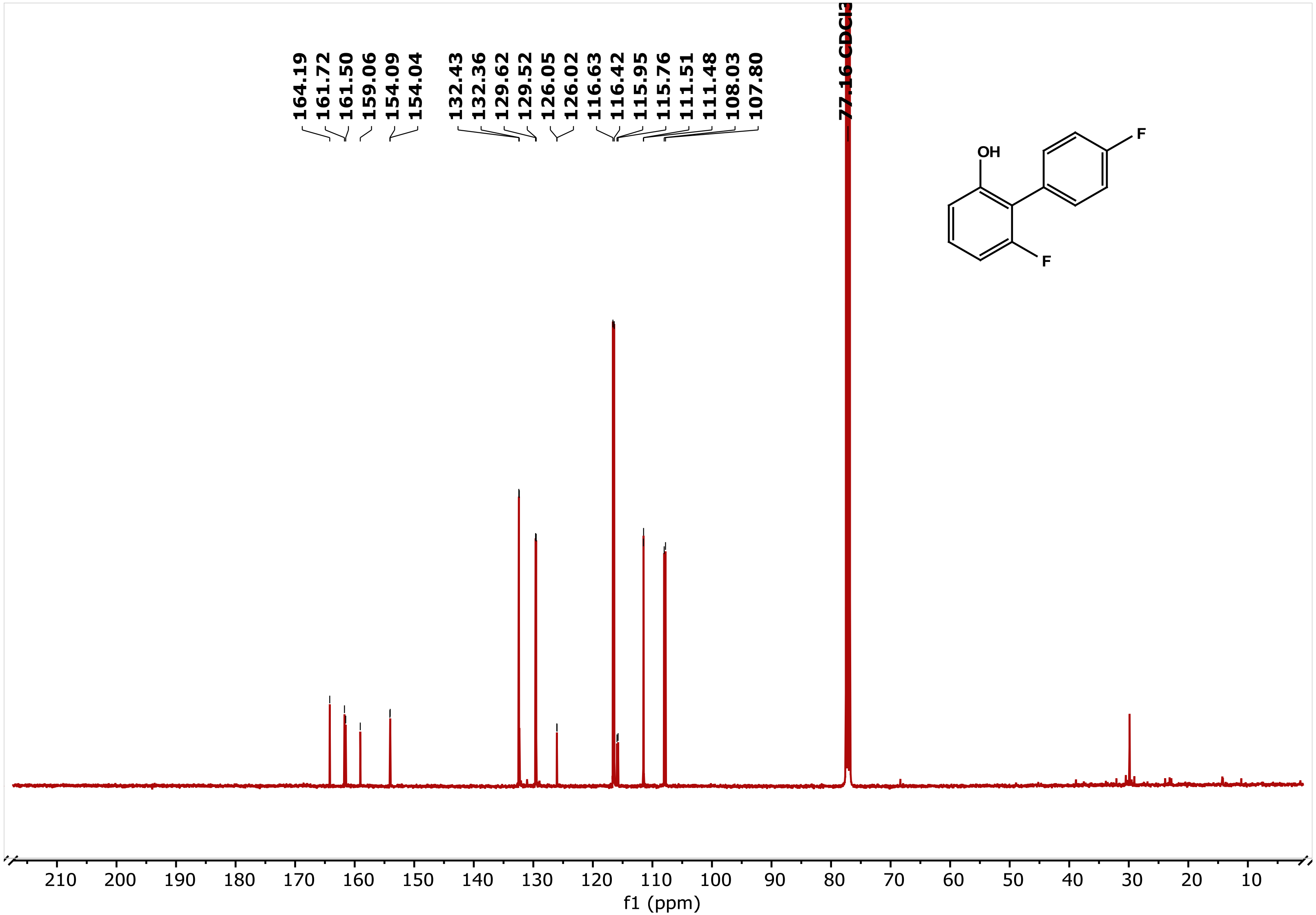

^{19}F NMR (377 MHz, $CDCl_3$):

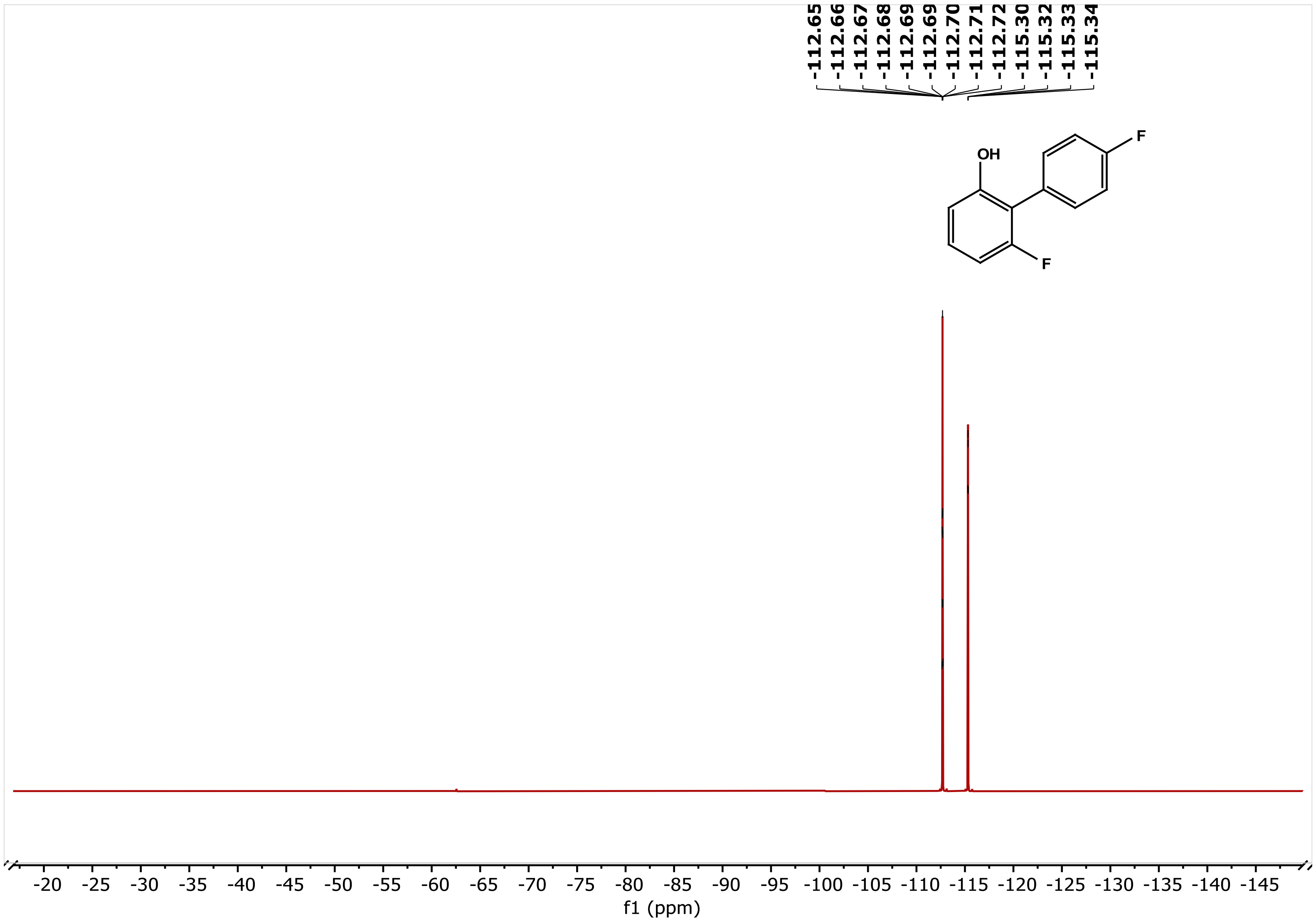

5,5-Dioxido-10H-dibenzo[b,e][1,4] thiabismin-10-yl 3-chlorobenzoate (1-OmCB)

^{1}H NMR (400 MHz, $CDCl_3$):

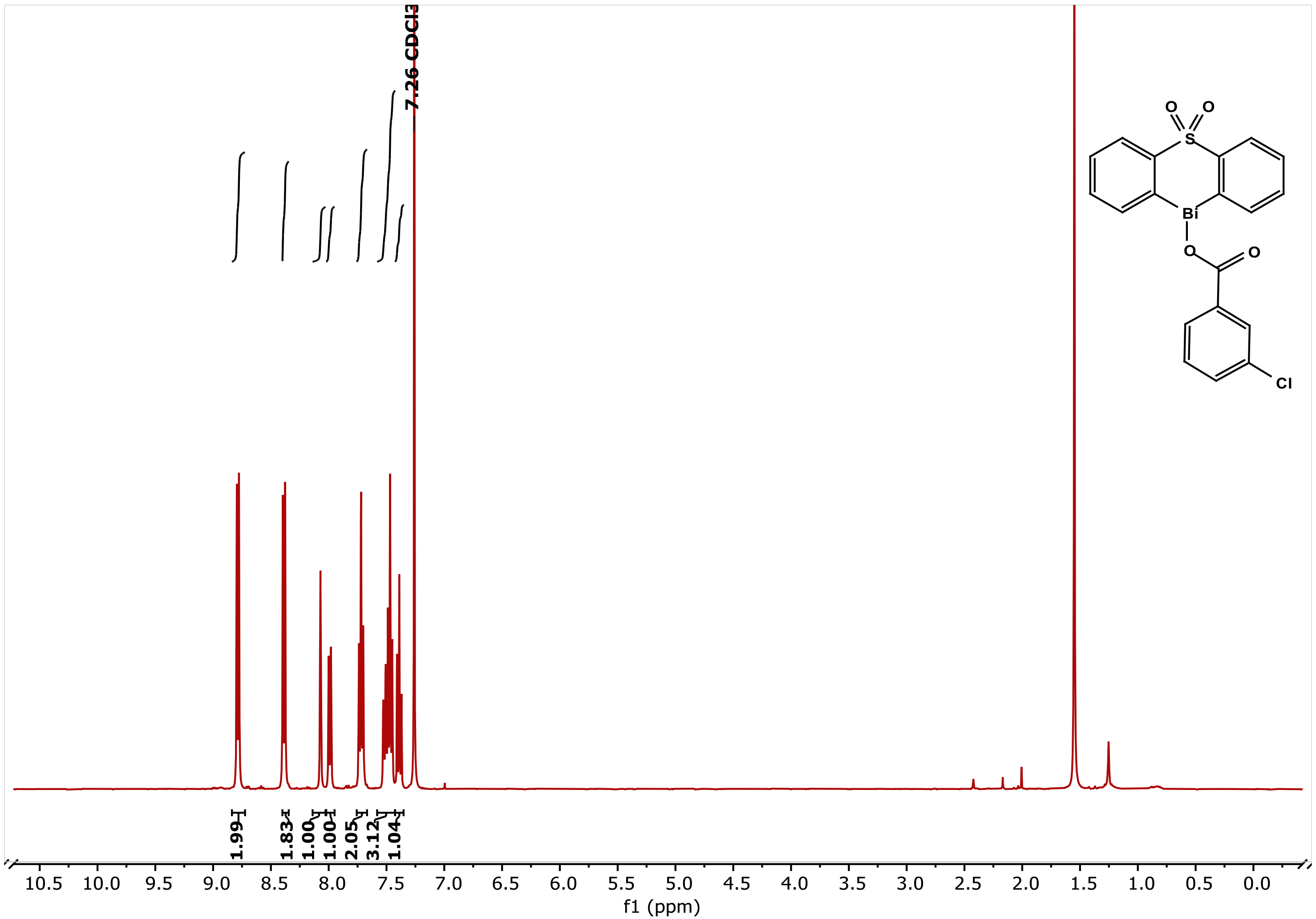

^{13}C{^{1}H} NMR (101 MHz, $CDCl_3$):

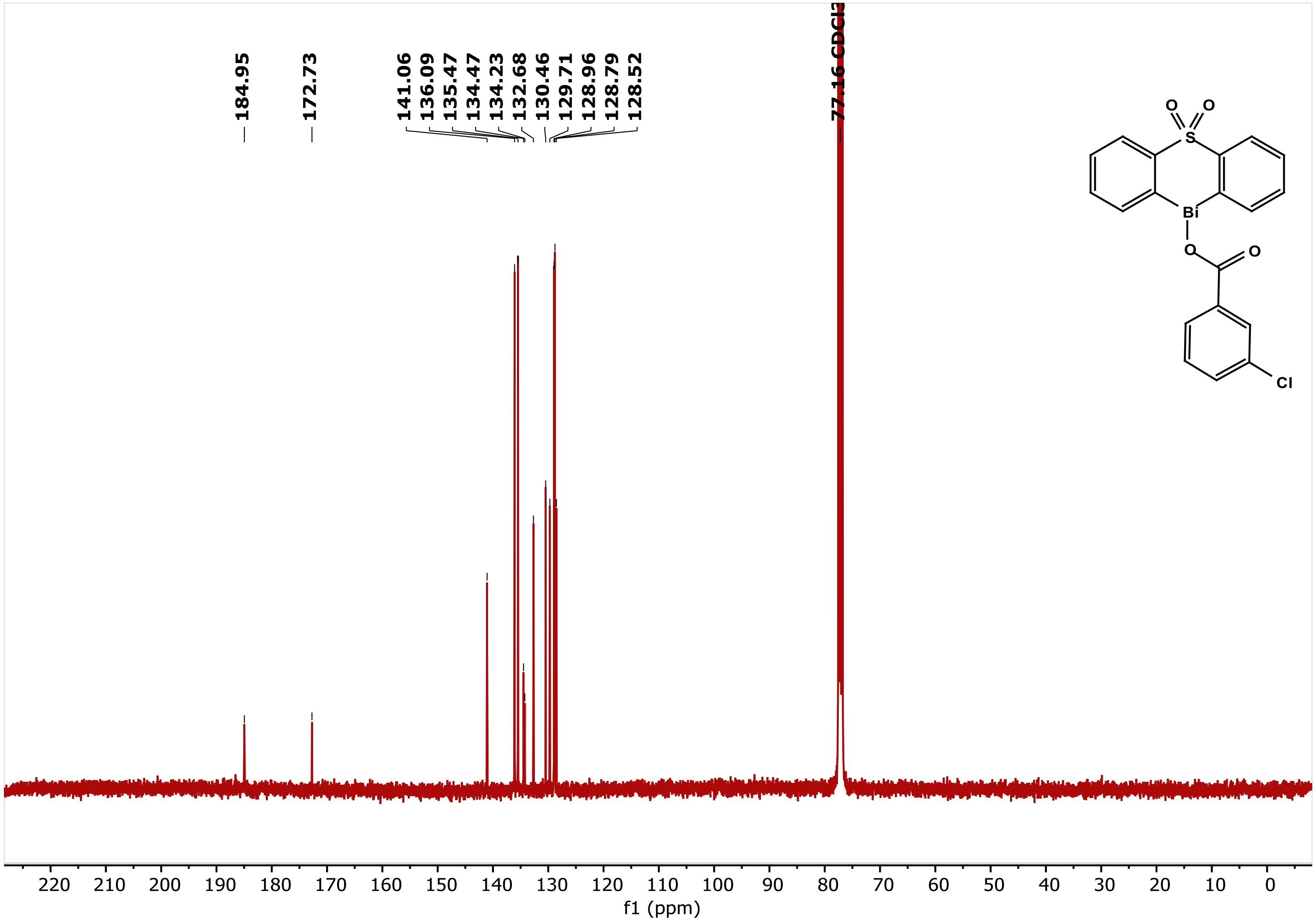

4,4''-Difluoro-[1,1':3',1''-terphenyl]-2'-ol

¹H NMR (400 MHz, $CDCl_3$):

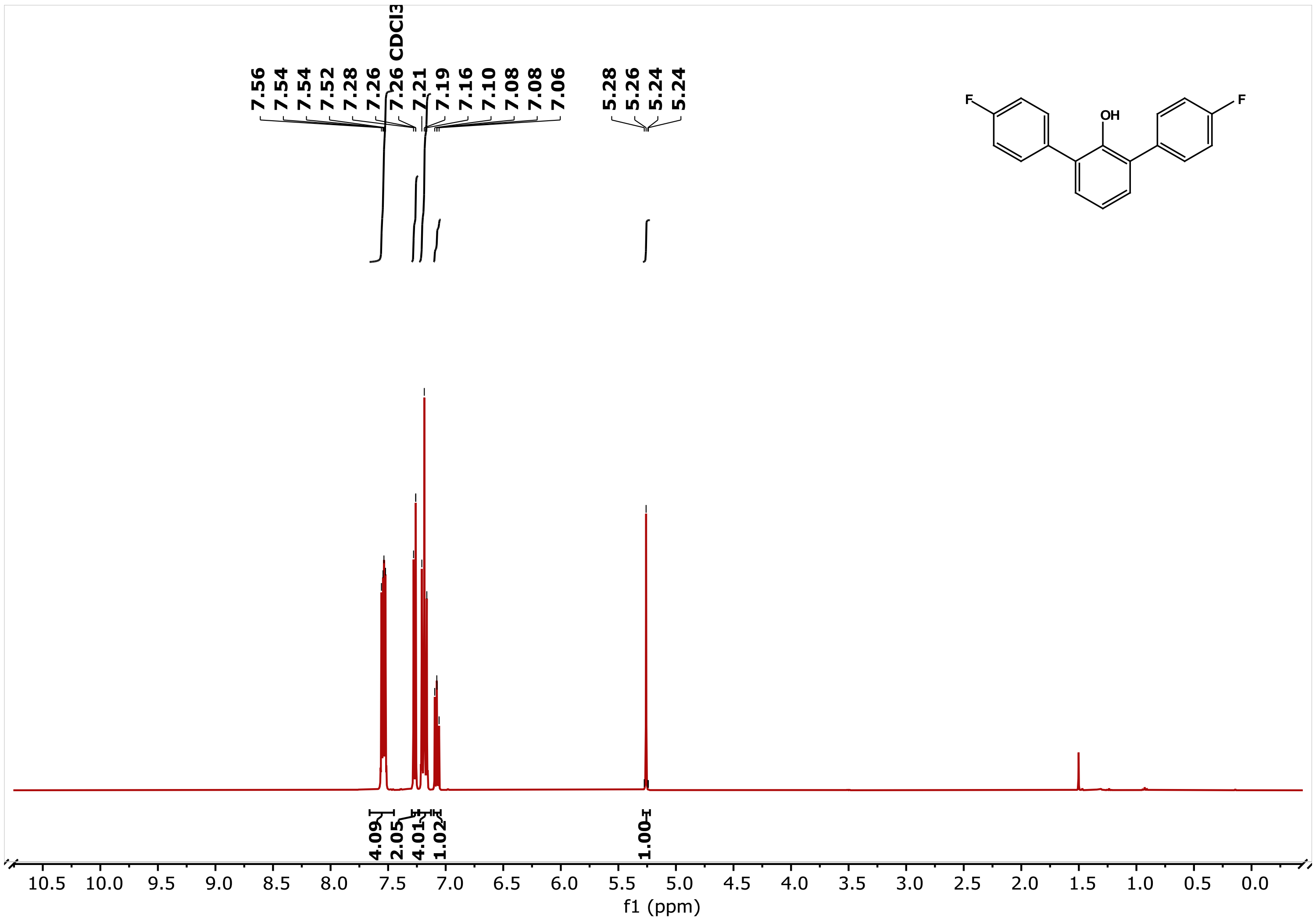

$^{13}C\{^1H\}$ **NMR** (101 MHz, $CDCl_3$):

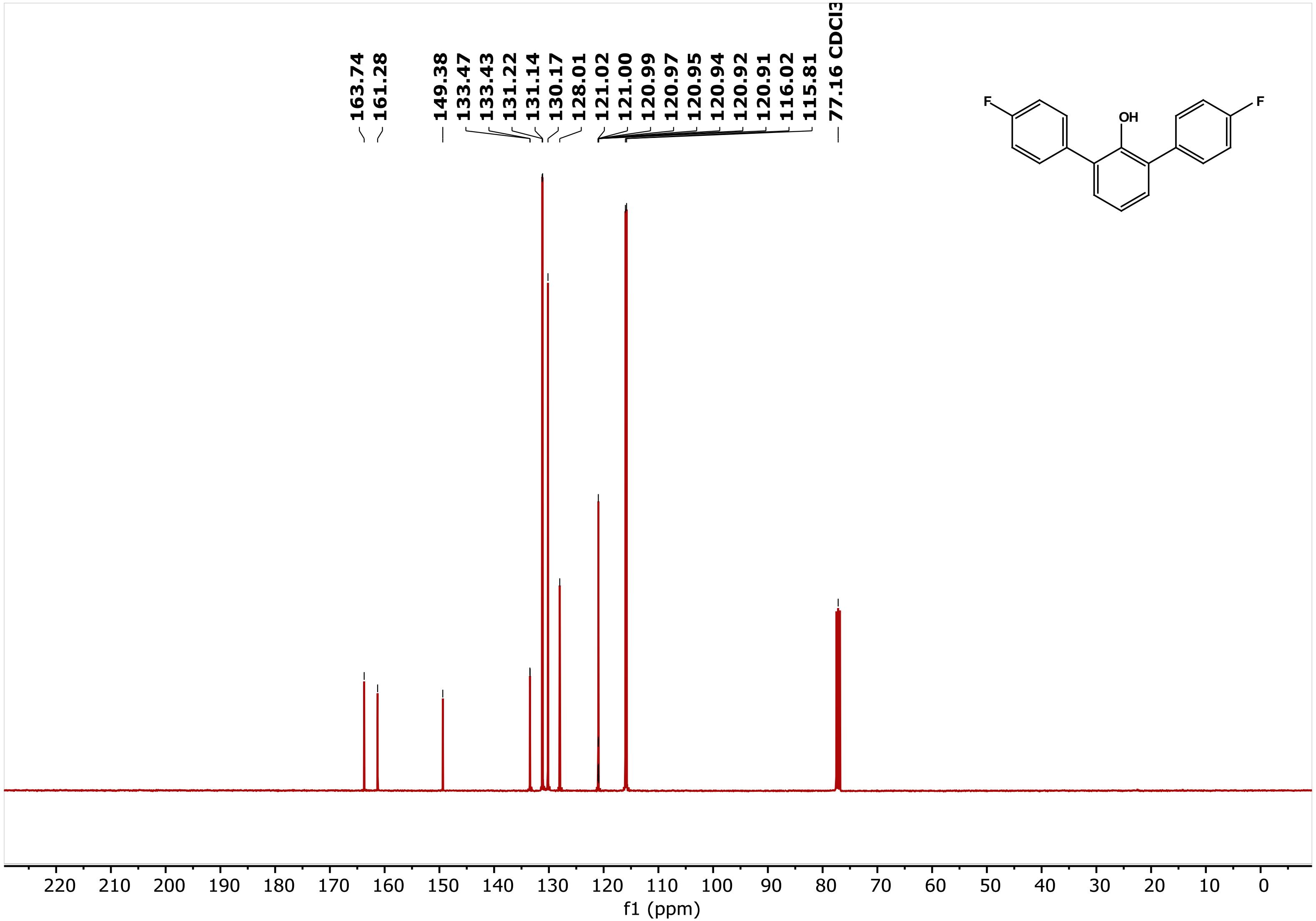

^{19}F NMR (376 MHz, $CDCl_3$):

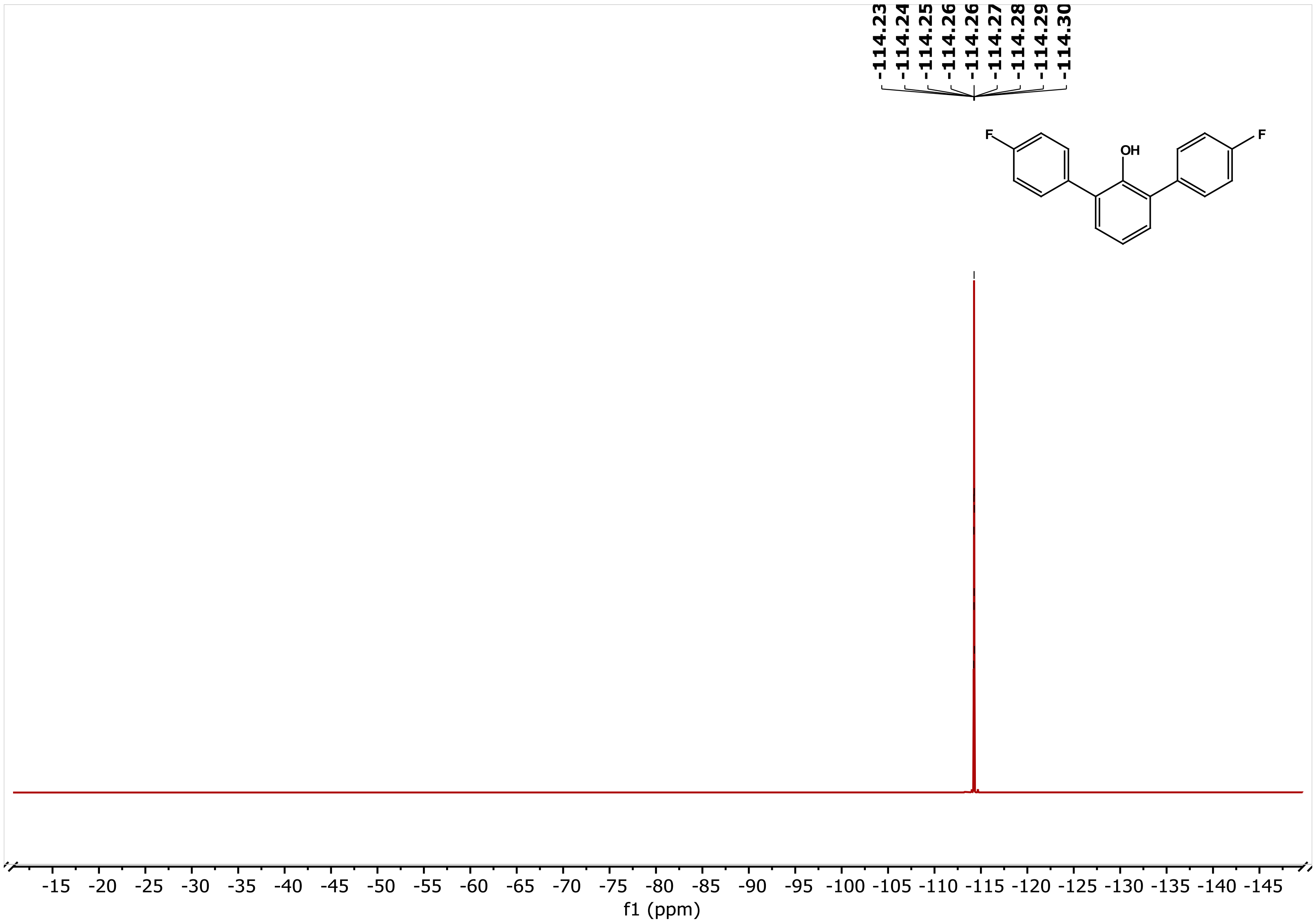

9. References

1 A. Fox and L. T. Ball, *Org Process Res Dev*, 2024, **28**, 632–639.

2 M. Jurrat, L. Maggi, W. Lewis and L. T. Ball, *Nature Chemistry 2020 12:3*, 2020, **12**, 260–269.

3 K. Urgin, C. Aubé, C. Pichon, M. Pipelier, V. Blot, C. Thobie-Gautier, E. Léonel, D. Dubreuil and S. Condon, *Tetrahedron Lett*, 2012, **53**, 1894–1896.

4 R. Zhu, Q. Sun, J. Li, L. Li, Q. Gao, Y. Wang and L. Fang, *Chemical Communications*, 2021, **57**, 13190–13193.

5 M. Nagumo, M. Ninomiya, N. Oshima, T. Itoh, K. Tanaka, A. Nishina and M. Koketsu, *Bioorg Med Chem Lett*, 2019, **29**, 2475–2479.

6 M. Lambert, L. Olsen and J. W. Jaroszewski, *Journal of Organic Chemistry*, 2006, **71**, 9449–9457.

7 S. Duan, Y. Xu, X. Zhang and X. Fan, *Chemical Communications*, 2016, **52**, 10529–10532.

8 M. L. N. Rao and S. Meka, *Tetrahedron Lett*, 2020, **61**, 151512.

9 J. Jian, J. Poater, R. Hammink, P. Tinnemans, C. J. McKenzie, F. M. Bickelhaupt and J. Mecinović, *ChemPhysChem*, 2020, **21**, 1092–1100.

10 K. Kikushima and Y. Nishina, *RSC Adv*, 2013, **3**, 20150–20156.

11 P. Wawrzyniak and J. Heinicke, *Tetrahedron Lett*, 2006, **47**, 8921–8924.

12 K. Wu, L. L. Wu, C. Y. Zhou and C. M. Che, *Angewandte Chemie International Edition*, 2020, **59**, 16202–16208.

13 D. J. Cooke, J. M. Cross, R. V. Fennessy, L. P. Harding, C. R. Rice and C. Slater, *Chemical Communications*, 2013, **49**, 7785–7787.
